# Application of Psychrotolerant Antarctic Bacteria and Their Metabolites as Efficient Plant Growth Promoting Agents

**DOI:** 10.3389/fbioe.2022.772891

**Published:** 2022-02-24

**Authors:** Michal Styczynski, Gabriel Biegniewski, Przemyslaw Decewicz, Bartosz Rewerski, Klaudia Debiec-Andrzejewska, Lukasz Dziewit

**Affiliations:** ^1^ Institute of Microbiology, Department of Environmental Microbiology and Biotechnology, Faculty of Biology, University of Warsaw, Warsaw, Poland; ^2^ Institute of Microbiology, Department of Geomicrobiology, Faculty of Biology, University of Warsaw, Warsaw, Poland

**Keywords:** Antarctica, biosurfactant, plant growth promoting bacteria, psychrotolerant bacteria, siderophore

## Abstract

Iron is the fourth most abundant element on earth. However, its low bioavailability is a key plant-growth limiting factor. Bacteria play an important role in plant growth promotion since they produce specific secondary metabolites that may increase macro- and micronutrient accessibility in soil. Therefore, bacterial-derived iron chelators, as well as surface-active compounds, are recognised as essential to plant welfare. In this study, three cold-active Antarctic bacterial strains, i.e. *Pseudomonas* sp. ANT_H12B, *Psychrobacter* sp. ANT_H59 and *Bacillus* sp. ANT_WA51, were analysed. The physiological and genomic characterisation of these strains revealed their potential for plant growth promotion, reflected in the production of various biomolecules, including biosurfactants (that may lower the medium surface tension of even up to 53%) and siderophores (including ANT_H12B-produced mixed-type siderophore that demonstrated the highest production, reaching the concentration of up to 1.065 mM), increasing the availability of nutrients in the environment and neutralising fungal pathogens. Tested bacteria demonstrated an ability to promote the growth of a model plant, alfalfa, increasing shoots’ length and fresh biomass even up to 26 and 46% respectively; while their metabolites increased the bioavailability of iron in soil up to 40%. It was also revealed that the introduced strains did not disrupt physicochemical conditions and indigenous soil microbial composition, which suggests that they are promising amendments preserving the natural biodiversity of soil and increasing its fertility.

## Introduction

Many microorganisms that are found in the rhizosphere may facilitate plant growth and, hence, are called plant growth-promoting bacteria (PGPB) or plant growth promoting rhizobacteria (PGPR) (Olanrewaju et al., 2017). Amongst well characterised rhizospheric PGPB are representatives of the following genera: *Agrobacterium*, *Azospirillum*, *Azotobacter*, *Bacillus*, *Pseudomonas*, *Streptomyces* (Glick, 2012), as well as *Psychrobacter* (Ma et al., 2011; García-López et al., 2014).

PGPB can affect plants, both through indirect and direct mechanisms. Indirect mechanisms mainly concern the inhibition of plant pathogens, i.e., fungi (e.g., *Fusarium tricinctum* (Chen T. et al., 2018)) and bacteria (e.g., *Pseudomonas syringae* (Morris et al., 2008)). This is a consequence of the production of hydrogen cyanide, antibiotics or fungistatic agents (Haas and Keel, 2003; Prashar et al., 2013). Direct mechanisms include: the production of plant hormones; the facilitation of nitrogen uptake; an increase in the bioavailability of phosphorus; cellulolytic and ligninolytic activity; the ability to break down complex compounds and xenobiotics; and the production of a wide variety of secondary metabolites, like biosurfactants (crucial in the mobilization of hydrophobic carbon sources) and iron-chelating agents (Antonella Di Benedetto et al., 2017; Olanrewaju et al., 2017).

Iron is essential for the growth and metabolism of living organisms. In the environment it occurs usually as Fe(III) in the form of oxides and hydroxides and has very low solubility in a pH above 3–4 (Crowley, 2006). Since, iron is a component of many vital enzymes, contributes to the biosynthesis of chlorophyll and is essential for the maintenance of chloroplast structure and function, its low bioavailability makes it a key plant growth-limiting element in nature (Rout and Sahoo, 2015). One of the most common and efficient mechanism of iron chelation is the production of siderophores. These are small compounds showing a high affinity mainly for iron (III) (Hider and Kong, 2010) and are produced in response to iron deficiency in the environment (Carroll and Moore, 2018). Based on the chemical structure of siderophores, they have been divided into four classes: hydroxamates, catecholates (also defined as phenolate), carboxylates and mixed ones (Carroll and Moore, 2018). Interestingly, plants have developed receptors enabling the uptake of bacterial siderophores. Thus, they may ‘steal’ these biomolecules using them as iron carriers, which increases the overall fitness of plants (Chu et al., 2010; Dimkpa, 2016). The beneficial influence of bacterial siderophores on plants’ growth relies also on their ability to limit the development of some pathogenic fungi and bacteria, by entailing iron deficiency in soil (Scavino and Pedraza, 2013). One of the best-studied siderophores, with direct antifungal properties, is pyoverdine produced by *Pseudomonas* spp., which contributes to the suppression of pathogens’ development, through increased iron competition—the fungal siderophores have a generally lower Fe(III) affinity (Sharma and Johri, 2003; Selvakumar et al., 2009; Scavino and Pedraza, 2013). In addition, many bacterial siderophores, like pyochelin, are capable of inducing systemic resistance in plants (Höfte and Bakker, 2007; Aznar and Dellagi, 2015).

Another secondary metabolite that may promote the plant growth are biosurfactants. These are surface active compounds of various chemical nature produced by microorganisms. They facilitate microbial adaptation in rhizosphere through stimulating biofilm formation on the plant roots surface, as well as improving bacterial motility (Sachdev and Cameotra, 2013; Alsohim et al., 2014). Moreover, bacterial surfactants increase the bioavailability of hydrophobic compounds in the soil and therefore increase its overall fertility and wettability. Importantly, biosurfactants are considered as effective antifungal agents, e.g. surfactin exhibits antagonistic activity against pathogenic fungi including *Fusarium* spp. and *Aspergillus* spp. (Joshi et al., 2008; Velho et al., 2011; Sachdev and Cameotra, 2013).

The crop vegetation cycles in the temperate climatic zone in the Northern hemisphere begin usually in early spring or even late winter, which are characterised by low temperatures and occasional frost. It is deleterious to the growth of mesophilic bacteria used as biofertilizers and their plant growth-promoting (PGP) activities. Under low temperatures, the majority of microbial activities are inhibited or slowed down (Mishra et al., 2010). Antarctic, cold-active PGPB are an excellent alternative to mesophilic strains in this situation. So far, several cold-active bacterial strains with plant growth promotion properties have been described, e.g. *Pantoea dispersa* and *Serratia marcescens*, which were able to promote wheat growth even at 5°C (Selvakumar et al., 2008a; Selvakumar et al., 2008b). However, nowadays, novel nature-based solutions for agriculture are still needed; thus, scientists are constantly searching for novel candidates for biofertilizers.

Here, we present a complex characterisation of three bacterial species, that is: *Pseudomonas* sp. ANT_H12B, *Psychrobacter* sp. ANT_H59 and *Bacillus* sp. ANT_WA51, originating from Antarctic soils. We reveal their plant growth-promoting activities such as: increasing the availability of phosphorus; utilizing of various complex compounds in the soil and production of plant hormones. However, we particularly focused on their ability to produce siderophores and biosurfactants, recognised as beneficial factors for plants (Amin et al., 2009; Pahari et al., 2018). All plant growth-promoting features were demonstrated in accordance with the genomic backgrounds of investigated bacteria. Moreover, the influence of bacteria and their metabolites on soil chemistry and the structure of soil microbial (both bacterial and fungal) communities was presented.

## Materials and Methods

### Bacterial Strains and Plant

The bacterial strains including *Pseudomonas* sp. ANT_H12B, *Psychrobacter* sp. ANT_H59 and *Bacillus* sp. ANT_WA51 were obtained from a collection of bacterial cultures that were previously isolated from the soil samples taken in 2012 from King George Island (Antarctica; GPS coordinates: 62 09.6010 S, 58 28.4640 W) (Romaniuk et al., 2018). Alfalfa (*Medicago sativa* L., TANGO type) was selected as a plant representing the Leguminosae family (legumes) of high agricultural importance (Kulkarni et al., 2018) and bioremediation potential (Debiec-Andrzejewska et al., 2020).

### Analysis of Siderophore Production

To determine the amount of produced siderophores, bacteria were cultivated for 7 days in GASN medium at 20°C with rotary shaking set to 150 rpm. The initial optical density at 600 nm (OD_600_) was 0.1. After 7 days of cultivation, bacteria were centrifuged (6,000 rpm for 5 min) and supernatants were added in a 1:1 ratio to the CAS (chrome azurol S) reagent (Schwyn and Neilands, 1987). GASN medium was used as a negative control, while deferoxamine mesylate salt (Sigma-Aldrich), at a concentration of 0.025 mM, was used as a positive control. All experiments were performed in triplicates. After an hour of incubation, the absorbance at 630 nm was measured using an automated microplate reader. Furthermore, Neilands (Neilands, 1981), Arnow (Arnow, 1937) and Shenker (Shenker et al., 1992) biochemical assays were used to determine hydroxamate, catecholate and carboxylate functional groups of siderophores, respectively.

### Analysis of Surfactant Production

To determine the presence of biosurfactants, bacterial cultures were cultivated for 7 days in LB medium at 20°C with rotary shaking set to 150 rpm in two variants: 1) with 1% (w/v) sunflower oil and 2) without this supplementation. The initial optical density at 600 nm (OD_600_) was 0.1. Additionally, the ability to produce surfactants was tested on GASN medium (without the addition of oil). The controls were uninoculated variants. The experiments were performed in triplicates. After 7 days of cultivation, bacteria were centrifuged (6,000 rpm for 5 min) and supernatants were tested through the ring method (du Noüy method) using a Kruss Tensiometer K20 (Kruss GmbH, Hamburg, Germany) (Malavenda et al., 2015).

### Analysis of Indole Acetic Acid Production

The production of indole acetic acid (IAA) was estimated in inoculated LB medium, supplemented with 5 mM tryptophan, at 20°C with rotary shaking set to 150 rpm in the dark. After 7 days of incubation, cultures were centrifuged (6,000 rpm for 5 min) and supernatants were used for the quantification of IAA. Salkowski reagent (0.5 M FeCl_3_ with 35% perchloric acid) was added to the culture supernatant at a 4:1 ratio and the absorbance (OD) was measured at 530 nm. As a control indole-3-acetic acid (Sigma-Aldrich) and uninoculated media were used. Experiments were performed in triplicate (Mohite, 2013; Pranaw et al., 2020).

### Ureolytic Activity

To determine the ureolytic activity of tested bacterial strains, the LB medium was supplemented with 1% (w/v) urea and 0.001% (w/v) phenol red (Sigma-Aldrich). The inoculated media were then cultivated for 7 days at 20°C with rotary shaking set to 150 rpm. The initial optical density at 600 nm (OD_600_) was 0.1. The supplemented medium, due to the presence of ammonia and higher pH turned pink. pH changes were additionally confirmed with a pH meter. The following controls were used: 1) inoculated LB supplemented with 0.001% phenol red; 2) inoculated LB supplemented with 1% urea; 3) non-inoculated LB supplemented with 1% urea and 0.001% phenol red (Sigurdarson et al., 2020). The experiments were performed in triplicate.

### Inorganic Phosphate Solubilisation

The phosphate solubilisation potential of bacterial strains was estimated using NBRIP medium (National Botanical Research institute’s phosphate growth medium) (Nautiyal, 1999). Cultures were grown overnight (OD_600_ ∼0.5) and spot-inoculated in triplicates of 5 µL on plates. Bacteria were then incubated at 20°C for 7 days. The presence of a halo zone around the areas of bacterial growth indicated the inorganic phosphate solubilisation potential (Rana et al., 2011).

### Cellulolytic Activity

To determine the cellulolytic activities of the tested bacteria, cultures were grown overnight (OD_600_ ∼0.5) and spot-inoculated in triplicates of 5 µL on plates with solid Mandels and Reese medium (Mandels and Reese, 1957) containing carboxymethyl cellulose (CMC) sodium salt (Sigma-Aldrich) (Liang et al., 2014). Bacteria were then incubated at 20°C for 7 days. After the incubation time, plates were flooded with an aqueous solution of Congo red (1 mg/ml) for 15 min. The Congo red solution was then poured off and visualised zones of hydrolysis were stabilised by flooding the agar with 1 M HCl (Teather and Wood, 1982).

### Proteolytic Activity

To examine the proteolytic activity of strains, bacteria were grown overnight (OD_600_ ∼0.5) and spot-inoculated in triplicates on LB agar plates supplemented with 1% (w/v) skimmed milk. The presence of a halo zone around the areas of bacterial growth indicated proteolytic activity (Jones et al., 2007).

### Lipolytic Activity

The lipolytic activity of the bacterial strains was tested on Egg-Yolk Agar on which bacteria were spot-inoculated in triplicates of 5 µL overnight (OD_600_ ∼0.5) cultures. After 7 days of incubation, saturated CuSO_4_ solution was poured into the plates and left 20 min for staining. Then, the excess solution was removed and a greenish-blue color confirmed lipolysis (Faiz, 2021).

### Preparation of Bacterial Cultures and Secondary Metabolites

To reach the maximum number of siderophores, bacterial strains were cultivated overnight in lysogeny broth (LB) medium at 20°C with rotary shaking set to 150 rpm. Cultures were then centrifuged (6,000 rpm for 5 min) and washed three times with 0.85% saline solution. Next, bacteria were added in triplicates into the fresh GASN medium (Bultreys and Gheysen, 2000) and cultivated at 20°C with rotary shaking set to 150 rpm. In each case, the initial optical density at 600 nm (OD_600_) was 0.1. The growth kinetics were assessed by measuring the changes in the optical density of cultures in comparison with the non-inoculated controls, using an automated microplate reader (Sunrise TECAN, Tecan Trading AG, Männedorf, Switzerland). The OD_600_ of the respective cultures was measured every 24 h for 7 days. Bacteria were then centrifuged (6,000 rpm for 5 min) and supernatants containing metabolites for the experiments were obtained through 0.22-µm pore size filtering. To obtain bacteria cells, overnight cultures in lysogeny broth were centrifuged (6,000 rpm for 5 min), washed three times and diluted in 0.85% saline solution to reach a final concentration of 10^8^ CFU (colony forming unit) per 1 g of soil.

### Soil Parameters

Potted garden soil, the properties of which were examined and described in the previous work (Debiec-Andrzejewska et al., 2020), was used for the experiments. The initial pH and redox potential were 6.37 (+/− 0.6) and 32.7 (+/− 0.58), respectively. Under such conditions iron bioavailability is limited, which refers to the conditions occuring naturally when cultivating alfalfa (optimal growth at pH of 6.5–7.5). Soil parameters were measured after 7 (T1), 14 (T2) and 21 (T3) days of experiment.

### Experimental Setup

Alfalfa was first pre-cultivated. The seeds of alfalfa plants were planted into soil without the addition of bacteria or their metabolites and were incubated for 7 days. The pre-culture was carried out in pots (600 mm long/180 mm width/140 mm depth), containing 2 kg of soil. After this time, young plants were transplanted into previously prepared soil, which was bioaugmented with ANT_H12B, ANT_H59 or ANT_WA51 strains and supplemented with their metabolites. Strains and metabolites were used separately. The experiment was carried out in identical pots (dimensions: 600 × 180 × 140 mm in length, width and height, respectively) containing 2 kg of soil for each of the eight variants, i.e.,: (i) control with a saline solution (0.85% NaCl); (ii–iv) soil bioaugmented with particular strains: ANT_H12B, ANT_H59 and ANT_WA51, respectively; (v) control with GASN medium and (vi-viii) soil containing supernatants with metabolites from: ANT_H12B (siderophores), ANT_H59 (siderophores) and ANT_WA51 (siderophores and biosurfactants), respectively. Each variant was performed in three replicates (pots) containing 25 plants per pot, equal to a total of 75 plants per variant (Supplementary Figure S1). The results were observed at the beginning of the experiment and after 7, 14 and 21 days, respectively. At every time point five plants from each of three replicates of a given variant were harvested for further analyses.

### Morphological Analysis of Plants

Length, growth rate, fresh weight (FW) and dry weight (DW) of roots and shoots of alfalfa were appointed at: the beginning (T0) and after 7 (T1), 14 (T2) and 21 (T3) days of experimentation. For DW determination, samples were dried at 30°C for 72 h (Michalska-Kacymirow et al., 2014).

### Analysis of Total Iron Concentration in Plants and Soil

To determine the total iron concentration in plant tissues, 0.1 g of sample was digested in 5 ml of the oxidizing mixture, i.e.,: 4.5 ml HNO_3_ (69%) and 0.5 ml H_2_O_2_ (35%) at 180°C for 30 min using the closed microwave system (Milestone Ethos Plus, Sorisole, Italy). Mineralisation of soil samples (0.3 g) was performed with the use of the same mixture of 69% HNO_3_ and 35% H_2_O_2_ (ratio 9:1) under the same conditions. Digested samples were transferred to plastic tubes and stored at 4°C. The amount of iron was measured by Flame Atomic Absorption Spectroscopy (FAAS) and Graphite Furnace Atomic Absorption Spectroscopy (GFAAS) using a Thermo Scientific SOLAAR M Series (TJA Solution, SOLAAR M, Cambridge, United Kingdom). In FAAS, the gas mixture was air and acetylene. The calibration curve range was 0–10 mg L^−1^ and the lower limit of quantification was 0.01 mg L^−1^. In GFAAS, graphite cuvette duty cycle was as follows: evaporation −100°C/30 s; incineration - 1,100°C/20 s; atomization - 2,100°C/3 s; cleaning - 2,500°C/3 s and the calibration curve range was 0–20 μg L^−1^ with a lower limit of quantification of 0.1 μg L^−1^. The deuterium lamp (TJA Solution, SOLAAR M, Cambridge, United Kingdom) was used for background correction. Iron standard solutions (Merck, Darmstadt, Germany) were prepared in 3% HNO_3_.

### Analysis of Bioavailable Iron Concentration in Soil

In order to determine the content of bioavailable iron in the soil, the extraction procedure was performed in accordance with Draft International Standard ISO/DIS 14870. The extraction solution consisted of 0.005 M diethylenetriaminepentaacetic acid, 0.01 M CaCl_2_ and 0.1 M triethylamine, buffered at pH 7.3. All reagents were purchased from Sigma-Aldrich (St. Louis, MO, United States). 5 g of air-dried soil was added to 10 ml of extraction solution and shaken for 2 hours (Barteková et al., 2006). The extracts were then filtered through 0.22-µm filters and submitted for further analysis. Distilled water was used as a control extraction solution. All extractions were performed in triplicates.

### Determination of the Quantity of Heterotrophic Bacteria in the Soil

To determine the number of heterotrophs in soil, bacteria were rinsed from soil by adding 2 g of fresh soil (1 g of dry weight) to 50 ml of sterile 0.85% NaCl solution and shaken for 24 h. The obtained extracts were spread on the LB solid medium and after 7 days of cultivation at 20°C, the quantity of bacteria (CFU, colony-forming units/mL) was determined.

### Activity Against Plant Pathogenic Fungi

Nine plant pathogenic fungi, i.e.,: *Thamnidium elegans* WA18081, *Aspergillus niger* WA50716, *Aspergillus ochraceus* WA72081, *Penicillium expansum* WA72083, *Botrytis cinerea* WA72082, *Alternaria* sp. WA67128, *Cladosporium* sp. WA72809, *Fusarium tricinctum* WA67200 and *Fusarium sporotrichioides* WA67190 were obtained from the Herbarium Universitatis Varsoviensis, Botanic Garden, University of Warsaw (WABG) (Poland). The antifungal activity of bacteria was assessed against all investigated plant pathogenic fungi on potato dextrose agar (PDA) medium (Dennis and Webster, 1971). Fungi were grown on PDA medium for 7 days at 20°C. After incubation, 1 cm plugs of mycelia of actively growing fungi were placed in the centers of new PDA plates. In the first variant, bacteria were spot-inoculated in sterile cylinders using 10 µL of bacterial culture. In the second variant, 10 µL of the respective bacterial metabolites were spot-injected into sterile cylinders. Bacteria, metabolites and controls (i.e., GASN medium and 0.85% (w/v) saline solution) were prepared in the same way as for the plant experiment. Fungi along with the appropriate bacteria or metabolites were cultured for 7 days at 20°C. The experiments were performed in triplicate.

### Draft Genome Sequencing

Genomic DNA of the *Pseudomonas* sp. ANT_H12B, *Psychrobacter* sp. ANT_H59 and *Bacillus* sp. ANT_WA51 was isolated using the cetyl trimethylammonium bromide (CTAB)/lysozyme method (Sambrook and Russell, 2001). Illumina TruSeq libraries for each strain were constructed following the manufacturer’s instructions. The genomic libraries were sequenced on an Illumina MiSeq instrument (using the v3 chemistry kit) (Illumina, San Diego, CA, United States) in the DNA Sequencing and Oligonucleotide Synthesis Laboratory (oligo.pl) at the Institute of Biochemistry and Biophysics, Polish Academy of Sciences, Warsaw. Raw reads were processed with the fastp (Chen S. et al., 2018) version 0.19.5 with the following flags: *-cut_by_quality3 --cut_window_size 10 --cut_mean_quality 25 --trim_poly_x --poly_x_min_len 15 --length_required 100*. Filtered reads were used for assembly with SPAdes version 3.11.1 (Bankevich et al., 2012) with *--careful* flag.

### Bioinformatic Analysis

Analysed bacterial genomes were automatically annotated using RAST (Aziz et al., 2008) on the PATRIC 3.6.8 (Wattam et al., 2017) web service and manually curated. Similarity searches were performed using BLAST programs (Altschul et al., 1997). The metabolic features were identified with the SEED viewer webserver (Overbeek et al., 2014), KEGG (Kyoto Encyclopedia of Genes and Genomes) Automatic Annotation System (KAAS) database (Moriya et al., 2007) and the bacterial version of the antiSMASH webserver (Blin et al., 2019). All options were selected with the default parameters. Additionally, for deeper metabolic investigation, the amino acid sequences were subjected to analysis with BLAST-KOALA (Kanehisa et al., 2016). The KO (KEGG Orthology) assignments were performed using a modified version of the internally used KOALA (KEGG Orthology And Links Annotation) algorithm (BLAST-KOALA) after the BLAST search against a non-redundant dataset of pangenome sequences (Kanehisa et al., 2016). To investigate the virulence factors of the tested strains, the VFDB database (Virulence Factors database) was used (Liu et al., 2019). To identify putative antibiotic resistance genes, we used the Resistance Gene Identifier (RGI) in the Comprehensive Antibiotic Resistance database (CARD) (Jia et al., 2017). Hits showing at least 50% identity with the reference protein were considered significant. Each hit was verified manually using BLASTp analysis.

For the analysis of plant-growth promoting genes distribution, amino acid sequences of 68 reference proteins involved in the following plant growth promoting processes were used: 2,4-diacetylphloroglucinol synthesis (genes: *phlA*, *phlB*, *phlC*, *phlD*); ACC deamination (*acdS*); acetoin and 2,3-butanediol synthesis (*budA*, *budB*, *budC*, *ilvI*); auxin synthesis (*ipdC*); GABA synthesis (*gabD*, *gabT*); hydrogen cyanide synthesis (*hcnA*, *hcnB*, *hcnC*); nitric oxide synthesis (*nirK*); nitrogen fixation (*nifA*, *nifD*, *nifE*, *nifF*, *nifH*, *nifK*); phosphate solubilization (*pqqB*, *pqqC*, *pqqE*, *pqqF*, *pqqG*, *pstA*, *pstB*, *pstC*); urease synthesis (*ureA*, *ureB*); achromobactin synthesis (*acsA*, *acsB*, *acsC*, *acsD*, *acsE*, *acsF*); bacillibactin synthesis (*dhbA*, *dhbB*, *dhbC*, *dhbE*, *dhbF*); pyoverdine synthesis (*pvdA*, *pvdE*, *pvdH*, *pvdL*, *pvdM*, *pvdN*, *pvdO*, *pvdP*, *pvdQ*, *pvdS*, *pvdY*); vibrioferrin synthesis (*pvsA*, *pvsB*, *pvsC*, *pvsD*, *pvsE*); surfactin synthesis (*srfAA*, *srfAB*, *srfAC*, *srfAD*) and fengycin synthesis (*fenA*, *fenB*, *fenC*, *fenD*, *fenE*) (Supplementary Data 1). The following strains (with the following assembly accession numbers presented in parentheses) were used for the comparative proteomic analysis: *Bacillus* sp. ANT_WA51 (GCF_008,369,185.1), *Bacillus* sp. 916 Contig1 (GCF_000,275,,785.1), *Bacillus* sp. JS (GCF_000,259,,365.1), *Bacillus* sp. RZ2MS9 (GCF_001,816,185.2), *B. amyloliquefaciens* BS006 (GCF_001,278,635.1), *B. amyloliquefaciens* Y2 (GCF_000,262,,385.1), *B. atrophaeus* GQJK17 (GCF_002,243,495.1), *B. atrophaeus* UCMB-5137 (GCF_000,385,,965.2), *B. cereus* T4S (GCF_017,356,065.1), *B. pumilus* TUAT1 (GCF_001,548,215.1), *B. subtilis* PTS-394 (GCF_000,507,,005.1), *B. velezensis* FZB42 (GCF_000,015,785.2), *B. velezensis* GQJK49 (GCF_002,192,235.1), *B. velezensis* LDO2 (GCF_003,073,455.1), *B. velezensis* W2 (GCF_000,732,,055.1), *B. velezensis* YAU B9601-Y2 (GCF_000,284,,395.1), *Pseudomonas* sp. ANT_H12B (GCF_008,369,325.1), *Pseudomonas* sp. B10 (GCF_900,156,235.1), *Pseudomonas* sp. UW4 (GCF_000,316,,175.1), *Pseudomonas* sp. VI4.1 (GCF_002,029,345.1), *P. chlororaphis* HT66 (GCF_000,597,,925.1), *P. fluorescens* SBW25 (GCF_000,009,225.2), *P. fluorescens* UM270 (GCF_000,836,,415.1), *P. protegens* CHA0 (GCF_900,560,965.1), *P. psychrotolerans* CS51 (GCF_006,384,975.1), *P. putida* BIRD-1 (GCF_000,183,,645.1), *P. putida* MTCC 5279 (GCF_000,411,,615.1), *P. seleniipraecipitans* D1-6 (GCF_001,839,645.1), *P. simiae* WCS417 (GCF_000,698,,265.1), *P. syringae* GR12-2 (GCF_001,698,815.1), *P. veronii* VI4T1 (GCF_002,029,325.1), *Psychrobacter* sp. ANT_H59 (GCF_008,369,225.1), *P. arcticus* 273–4 (GCF_000,012,305.1), *P. cryohalolentis* K5 (GCF_000,013,905.1). Proteomes were compared against reference protein sequences using BLASTp and only the first hit for each of them was retained for further analysis if following thresholds were passed: *e*-value not higher than 1e-5, sequence identity and sequence query coverage per HSP of at least 50 and 70%, respectively.

### Analysis of the Changes in Soil Microbiome Composition

To determine changes in the soil microbial composition after bioaugmentation with selected bacteria or their metabolites, the DNA from control and supplemented soil was extracted from three pooled replicate 500-mg samples with the use of a FastDNA^®^ SPIN Kit for Feces DNA Extraction Kit and bead beater (MP Biomedica, Santa Ana, CA, United States) according to the manufacturer’s recommendations. The concentration and the quality of the extracted DNA were estimated using NanoDrop 2000 (Thermo Scientific, Waltham, MA, United States) and gel electrophoresis.

Used PCR primers covered: 1) V3–V4 regions of bacterial 16S rDNA gene (16S–V3–F: 5′-TCGTCGGCAGCGTCAGATGTGTATAAGAGACAGCCTACGGCWGCAG-3′ and 16S–V4-R: 5′-GTCTCGTGGGCTCGGAGATGTGTATAAGAGACAGGACTACHVGGGTATCTAATCC-3′) and 2) ITS1-ITS2 regions of eukaryotic internal transcribed spacers (ITS-ITS1-F: 5′-TCG​TCG​GCA​GCG​TCA​GAT​GTG​TAT​AAG​AGA​CAG​GCA​TCG​ATG​AAG​AAC​GCA​G-3′ and ITS-ITS2-R: 5′-GTC​TCG​TGG​GCT​CGG​AGA​TGT​GTA​TAA​GAG​ACA​GTC​CTC​CGC​TTA​TTG​ATA​TGC-3′). The metagenomics DNA was used as a template for amplification. The PCR was carried out in triplicate for each sample in a Mastercycler Nexus GX2 thermocycler (Eppendorf, Hamburg, Germany). The PCR amplification for 16S rDNA regions was performed according to the following process: 1) initial denaturation at 95°C for 5 min, 2) 25 cycles of the denaturation at 95°C for 30 s, primer attachment at 60°C for 30 s, DNA strand synthesis at 72°C for 30 s and 3) the final DNA strand extension at 72°C for 5 min. The PCR conditions for amplification of ITS regions were as follows: 1) initial denaturation at 95°C for 5 min, 2) 25 cycles of the denaturation at 95°C for 30 s, primer attachment at 60°C for 30 s, DNA strand synthesis at 72°C for 90 s and 3) the final DNA strand extension at 72°C for 5 min. The reaction mixture (25 μL) contained template DNA (20 ng), dNTP mix (400 nM of each the deoxynucleotides), MgCl_2_ (2 mM), Kapa High Fidelity polymerase (0.5 U), specific primers (400 nM of each primer) and 5× Fidelity buffer (1×) for 16S rDNA amplification or 5× GC buffer (1×) for ITS region.

Approximately 250 ng of each amplicon (pooled from replicate PCRs) were used for the library preparation. Libraries were verified using the 2,100 Bioanalyzer (Agilent) High-Sensitivity DNA Assay and KAPA Library Quantification Kits for the Illumina. Amplicon libraries were prepared with Illumina Nextera NT adapters (Illumina) and the sequencing of those DNA amplicons was performed with the Illumina MiSeq Platform (Illumina) using MiSeq Illumina Kit in the paired-end in the DNA Sequencing and Oligonucleotide Synthesis Laboratory—oligo. pl (Institute of Biochemistry and Biophysics, Polish Academy of Science). The sequencing provided from 108,027 to 214,779 paired reads for bacterial amplicons and from 96,605 to 251,871 reads for fungal amplicons.

Complete analysis of bacterial and fungal diversity was carried out using the QIIME2 framework version 2020.08 (Bolyen et al., 2019). Briefly, raw reads were imported to QIIME2, primers were removed with the CutAdapt plugin (Martin, 2011) and trimmed reads were processed with the DADA2 plugin without any further trimming, in independent pooling denoising mode and with the application of consensus chimera detection method (Callahan et al., 2016). The number of obtained amplicon variant sequences (ASVs) was sufficient to cover the whole biodiversity for both bacterial and fungal analysis as revealed by diversity alpha-rarefaction plugin analysis. Taxonomic classification of ASVs was performed with the application of a pretrained naïve Scikit-learn classifier based on SILVA SSU NR99 reference database version 138 (Quast et al., 2013) for bacterial samples and VSEARCH (Rognes et al., 2016) using 85% sequence identity and 90% sequence coverage as thresholds based on UNITE dynamic database version 8.2 for fungal samples (Nilsson et al., 2019). Alfa and beta diversity metrics were generated using the following QIIME2 plugins: phylogeny, diversity and emperor.

### Statistical Analysis

The significance of the differences between the mean values of the control and treated plant and soil samples was statistically evaluated by a two-tailed *t*-test at *p* ≤ 0.05. The Mann–Whitney–Wilcoxon test was applied whenever data failed to present a normal distribution or had different variances. The statistical analysis was carried out using the XLSTAT (version 2021.1) program. Principal component analysis (PCA) analysis was computed in R v4.1.2 using *prcomp* function with scaling and centering. The results were presented with ggplot2 v3.3.5 (Wickham, 2016) and ggbiplot v0.55 (Vu, 2011) packages.

## Results

### General Physiological Characterisation of *Pseudomonas* sp. ANT_H12B, *Psychrobacter* sp. ANT_H59 and *Bacillus* sp. ANT_WA51

All tested bacteria, i.e., *Pseudomonas* sp. ANT_H12B, *Psychrobacter* sp. ANT_H59 and *Bacillus* sp. ANT_WA51, were isolated from the extreme Antarctic environment (Romaniuk et al., 2018). These strains exhibited features characteristic for extremophiles. The ANT_H12B and ANT_H59 strains were able to grow in a wide range of temperatures, i.e., 4–30°C and 4–37°C, respectively, while ANT_WA51 in the range of 10–37°C. All tested strains exhibited halophilic properties, i.e., ANT_H12B could survive in 4% NaCl solution, ANT_H59 in 6%, while ANT_WA51, due to its ability to form spores, was able to survive in up to 10% NaCl solution. The tested strains were active in a wide range of pH, i.e., ANT_H12B was able to grow in a pH from 4 to 12, ANT_H59 in a pH from 6 to 11 and ANT_WA51 in a pH from 5 to 11 (Romaniuk et al., 2018).

All tested strains indicated potential useful features in plant growth promotion. Analyses revealed that two strains, i.e. *Pseudomonas* sp. ANT_H12B and *Bacillus* sp. ANT_WA51, were able to dissolve inorganic phosphorus compounds (e.g. Ca_3_(PO_4_)_2_), degrade cellulose and produce plant hormones such as indole acetic acid (IAA). Moreover, ANT_H12B and ANT_WA51 exhibited proteolytic abilities. What is important, *Psychrobacter* ANT_H59 and *Bacillus* ANT_WA51 were able to break down lipids and produce surfactants. In the case of ANT_H59, the interfacial tension (IFT) analysed on oil-supplemented LB medium was reduced from 55 (+/− 1) to 32 (+/− 1). ANT_WA51 produced surfactants constitutively (i.e., without induction with oil) on LB medium and lowered the surface tension from 55 (+/− 1) to 26 (+/− 1). Furthermore, only *Bacillus* sp. ANT_WA51 produced surface-active agents on GASN medium and lowered the IFT from 55 (+/− 2) to 33 (+/− 1) mN/m. Additionally, all three tested strains produced various types of siderophores, which were identified biochemically and measured spectrophotometrically in CAS reagent using deferoxamine as a reference agent. The ANT_H12B produced a mixed-type siderophore and demonstrated the highest production—1.065 mM (+/− 0.09), while ANT_H59 and ANT_WA51 produced respectively 0.188 mM (+/− 0.005) of carboxylate and 0.305 mM (+/− 0.028) of catecholate siderophores.

### Experimental Evaluation of Antifungal Properties of Analyzed Strains and Their Metabolites

**FIGURE 1:**
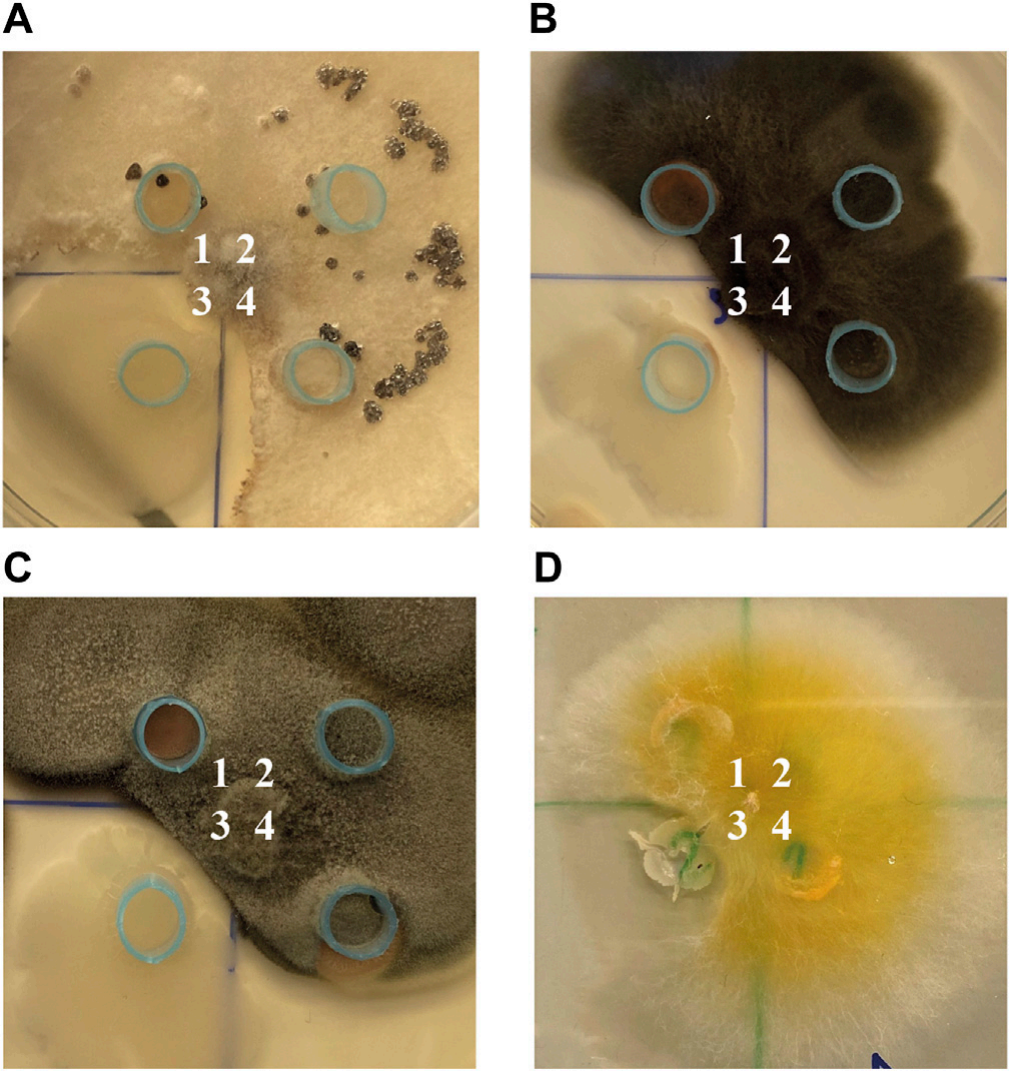
The antifungal activity of: ANT_H59 strain (1), GASN medium, as control (2), ANT_WA51 strain (3) and ANT_H12B strain (4) against: **(A)** *B. cinerea* WA72082, **(B)** *Alternaria* sp. WA67128, **(C)** *Cladosporium* sp. WA72809 and **(D)** *F. tricinctum* WA67200.

One of the main attributes of plant growth-promoting microorganisms is their ability to inhibit the growth of pathogens. It was shown, that only *Bacillus* sp. ANT_WA51 exhibited fungistatic activity and was effective against *B. cinerea* WA72082, *Alternaria* sp. WA67128, *Cladosporium* sp. WA72809 and *F. tricinctum* WA67200 (Figure 1).

### Overall Genomic Characterisation

Sequencing of the *Pseudomonas* sp. ANT_H12B, *Psychrobacter* sp. ANT_H59 and *Bacillus* sp. ANT_WA51 genomes generated 1,914,430 paired-reads and 513.705,394 nucleotides, 2,013,190 paired-reads and 601,632,051 nucleotides and 2,183,916 paired-reads and 654,484,729 nucleotides, respectively. Assembled genomes were deposited in the GenBank database under the following organism accession numbers: VOBI00000000—*Pseudomonas* sp. ANT_H12B, VOBE00000000—*Psychrobacter* sp. ANT_H59 and VOBB00000000—*Bacillus* sp. ANT_WA51. The genomic features for the three strains are presented in Table 1.

**TABLE 1:** General features of the *Pseudomonas* sp. ANT_H12B, *Psychrobacter* sp. ANT_H59 and *Bacillus* sp. ANT_WA51 draft genomes.

Feature	Calculation		
Strain	*Pseudomonas* sp. ANT_H12B	*Psychrobacter* sp. ANT_H59	*Bacillus* sp. ANT_WA51
Number of contigs	57	17	19
Estimated genome size (bp)	6,276,261	3,005,898	4,260,578
GC content (%)	58.57%	42.29%	43.28%
Number of genes	6,168	2,620	4,616
Number of proteins with functional assignments	4,835	2,053	3,745
Number of proteins with Enzyme Commission (EC) number assignments	1,338	783	1,076
Number of transfer RNAs (tRNAs)	59	42	83
Number of regulatory RNAs	6	3	27

### Genome-Based Insight Into General Metabolic Potential

The analysis of the metabolic potential of the *Pseudomonas* sp. ANT_H12B strain revealed various genetic pathways of the carbohydrate metabolism, including: glycolysis (Embden-Meyerhof pathway), gluconeogenesis, the citrate cycle (Krebs cycle), oxidative and non-oxidative phase of pentose phosphate cycle, Entner-Doudoroff glucose catabolism, d-galactonate degradation (De Ley-Doudoroff pathway) and glycogen degradation. Moreover, ANT_H12B possesses genes responsible for beta-oxidation of fatty acids and degradation of acylglycerols. In the genome of ANT_H12B, the following enzymes involved in nitrate metabolism are present: 1) large and small subunit of nitrite reductase (NADH) (EC: 1.7.1.15) (GenBank accession numbers: KAA0957090, KAA0957091, respectively); 2) alpha, beta and gamma subunits of nitrate reductase (EC: 1.7.5.1) (GenBank accession numbers: KAA0967878, KAA0967879, KAA0967881, respectively); 3) ferrocytochrome:nitrate oxidoreductase (EC: 1.9.6.1) along with its electron transfer subunit (GenBank accession numbers: KAA0980329, KAA0980330, respectively); 4) nitrous-oxide reductase (EC: 1.7.2.4) (GenBank accession numbers: KAA0967836, KAA0954707); 5) nitric oxide reductase (EC: 1.7.2.5) subunit B (GenBank accession numbers: KAA0973007, KAA0967859, KAA0951541) and subunit C (GenBank accession numbers: KAA0967858, KAA0951542); and 6) NO-forming nitrite reductase (EC:1.7.2.1) (GenBank accession number: KAA0967854). Furthermore, ANT_H12B encodes pathways responsible for assimilatory sulfates reduction into sulphides, i.e. 1) alpha and beta components of sulfite reductase (NADPH) (EC: 1.8.1.2) (GenBank accession numbers: KAA0964218 and KAA0968679, respectively); 2) thioredoxin (EC: 1.8.4.8) (GenBank accession number: KAA0957116); 3) CysN/CysC bifunctional enzyme (EC: 2.7.7.4) (GenBank accession number: KAA0971378); and 4) subunit two of sulfate adenylyltransferase (EC: 2.7.7.4) (GenBank accession number: KAA0971377).

The ANT_H12B strain possesses also genes encoding enzymes of metabolic pathways crucial for the breakdown of various xenobiotics like: 1) benzoates, i.e.: alpha and beta subunit of benzoate 1,2-dioxygenase (EC: 1.3.1.25) (GenBank accession numbers: KAA0968753, KAA0968752, respectively); dihydroxycyclohexadiene carboxylate dehydrogenase (EC: 1.3.1.25) (GenBank accession number: KAA0968750) and benzoate 1,2-dioxygenase reductase (EC: 1.18.1.-) (GenBank accession number: KAA0968751), 2) anthranilates, i.e.: small, large and reductase subunits of anthranilate 1,2-dioxygenase (EC:1.14.12.1) (GenBank accession numbers: KAA0975371, KAA0975370, KAA0975372, respectively), 3) catechols (through ortho-cleavage), i.e.: 3-oxoadipate enol-lactonase (EC: 3.1.1.24) (GenBank accession numbers: KAA0973994, KAA0953176, respectively); muconate cycloisomerase (EC: 5.5.1.1) (GenBank accession number: KAA0968757); catechol 1,2-dioxygenase (EC: 1.13.11.1) (GenBank accession number: KAA0968755); and muconolactone d-isomerase (EC: 5.3.3.4) (GenBank accession number: KAA0968756), as well as 4) halide impurities, i.e.,: 2-haloacid dehalogenase (EC:3.8.1.2) (GenBank accession number: KAA0975607), haloacetate dehalogenase (EC:3.8.1.3) (GenBank accession numbers: KAA0980549, KAA0969117, respectively), tetrachlorobenzoquinone reductase (EC:1.1.1.404) (GenBank accession number: KAA0968799) and carboxymethylenebutenolidase (EC:3.1.1.45) (GenBank accession numbers: KAA0980307, KAA0980364, KAA0975367, respectively).

Within the genome of the next analysed Antarctic strain, *Psychrobacter* sp. ANT_H59, several pathways of carbohydrate degradation were found, i.e.: the citrate cycle (Krebs cycle), non-oxidative phase of pentose phosphate cycle, Entner-Doudoroff glucose catabolism and the glyoxylate cycle. The ANT_H59 also encodes complete metabolic pathways responsible for beta-oxidation of fatty acids, degradation of acylglycerols and digestion of amino acids like: histidine, methionine and leucine. Furthermore, similarly to *Pseudomonas* sp. ANT_H12B, the ANT_H59 strain possesses genes encoding enzymes potentially contributing to: 1) benzoate degradation, i.e.: alpha and beta subunit of benzoate 1,2-dioxygenase (EC:1.3.1.25) (GenBank accession numbers: KAA0939514, KAA0939515, respectively); dihydroxycyclohexadiene carboxylate dehydrogenase (EC:1.3.1.25) (GenBank accession number: KAA0939517) and benzoate 1,2-dioxygenase reductase (EC:1.18.1.-) (GenBank accession number: KAA0939516), 2) catechol ortho-cleavage (i.e. 3-oxoadipate enol-lactonase (EC:3.1.1.24) (GenBank accession numbers: KAA0939522, KAA0930838, respectively); muconate cycloisomerase (EC:5.5.1.1) (GenBank accession number: KAA0939511); catechol 1,2-dioxygenase (EC:1.13.11.1) (GenBank accession number: KAA0939513); and muconolactone d-isomerase (EC:5.3.3.4) (GenBank accession number: KAA0939512) and 3) dehalogenation, i.e.: 2-haloacid dehalogenase (EC:3.8.1.2) (GenBank accession number: KAA0939827) and haloacetate dehalogenase (EC:3.8.1.3) (GenBank accession number: KAA0939528).

In the genome of *Bacillus* sp. ANT_WA51, similar metabolic pathways of carbohydrate degradation as in the ANT_H12B were identified, i.e.: glycolysis (Embden-Meyerhof pathway), gluconeogenesis, the citrate cycle (Krebs cycle), oxidative and non-oxidative phase of pentose phosphate cycle and Entner-Doudoroff glucose catabolism. Moreover, metabolic pathways of d-galacturonate, d-glucuronate, galactose, glycogen and histidine degradation were also identified in this strain. Deeper investigation of the ANT_WA51 genome revealed the presence of genes involved in: 1) dissimilatory nitrate reduction, i.e.: large (GenBank accession numbers: KAA0933206, KAA0933208) and small (GenBank accession number: KAA0933209) subunits of nitrite reductase (NADH) (EC: 1.7.1.15); alpha, beta and gamma subunits of nitrate reductase (EC: 1.7.5.1) (GenBank accession numbers: KAA0934458, KAA0934457 and KAA0934455, respectively) and 2) assimilatory sulfate reduction (i.e.: alpha and beta components of sulfite reductase (NADPH) (EC: 1.8.1.2) (GenBank accession numbers: KAA0934095 and KAA0934094, respectively); thioredoxin (EC: 1.8.4.8) (GenBank accession numbers: KAA0936206, KAA0936678); adenylylsulfate kinase (EC: 2.7.1.25) (GenBank accession number: KAA0936204, KAA0936681); and sulfate adenylyltransferase (EC: 2.7.7.4) (GenBank accession numbers: KAA0936205, KAA0936680). Furthermore, *Bacillus* sp. ANT_WA51 contains an encoded ribulose monophosphate pathway (i.e.: 6-phosphofructokinase 1 (EC: 2.7.1.11), fructose-bisphosphate aldolase, class II (EC: 4.1.2.13). 3-hexulose-6-phosphate synthase (EC: 4.1.2.43) and 6-phospho-3-hexuloisomerase (EC: 5.3.1.27) (GenBank accession numbers: KAA0938057, KAA0934442, KAA0933193 and KAA0933194, respectively), which are associated with the metabolism of C1 compounds.

Additionally*, Bacillus* sp. ANT_WA51 carries genes whose products are potentially involved in the decomposition of xenobiotics, such as: 1) benzoate compounds, i.e.: carboxymuconolactone decarboxylase (EC: 4.1.1.44) (GenBank accession numbers: KAA0937823, KAA0932560), 4-oxalocrotonate tautomerase (EC: 5.3.2.6) (GenBank accession number: KAA0934489) and catechol 2,3-dioxygenase (EC: 1.13.11.2); 2) aminobenzoates (vanillin dehydrogenase (EC: 1.2.1.67) (GenBank accession number: KAA0930986), C and D subunit 4-hydroxybenzoate decarboxylase (EC: 4.1.1.61) (GenBank accession numbers respectively: KAA0933177, KAA0933176), 4-nitrophenyl phosphatase (EC: 3.1.3.41) (GenBank accession number: KAA0933977) and 3) halides (i.e., 2-haloacid dehalogenase (EC: 3.8.1.20) (GenBank accession number: KAA0930988).

### Genome-Based Insight Into Plant Growth Promoting Traits

Each of the analysed strains possesses different repertoire of genes and thus specific metabolic features, which also refers to plant growth promoting traits.

**FIGURE 2:**
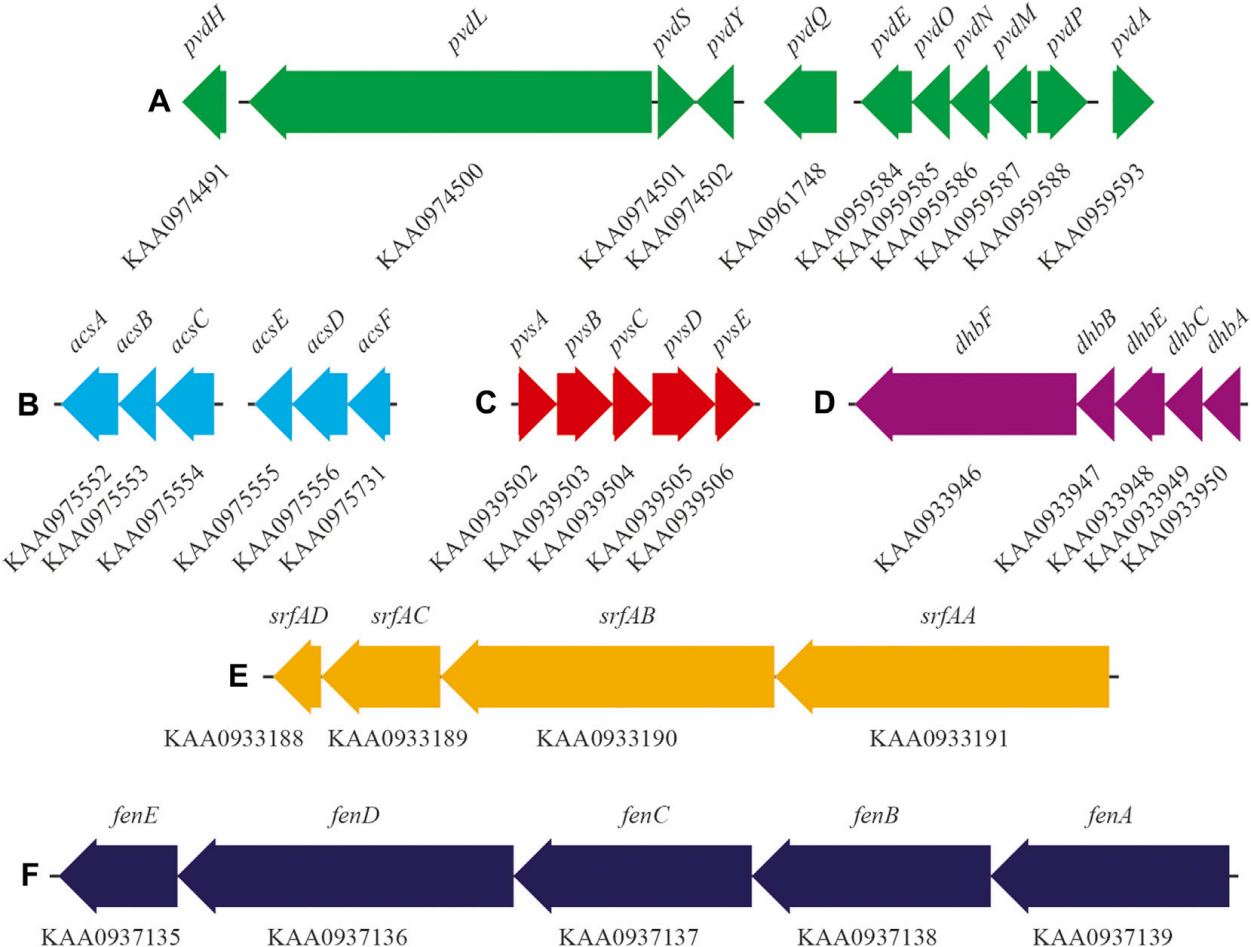
Gene clusters responsible for the biosynthesis of siderophores, i.e.,: **(A)** pyoverdine and **(B)** achromobactin of *Pseudomonas* sp. ANT_H12B; **(C)** vibrioferrin of *Psychrobacter* sp. ANT_H59; **(D)** bacillibactin of *Bacillus* sp. ANT_WA51 and biosurfactants, i.e.,: **(E)** surfactin and **(F)** fengycin of ANT_WA51. Gene names and thair GenBank accession numbers are displayed above and under the arrows, respectively.

In the genome of *Pseudomonas* sp. ANT_H12B, we found various genes encoding enzymes responsible for the production of plant growth promotion agents. There are genes encoding alpha and beta subunits of urease (EC: 3.5.1.5) (GenBank accession numbers: KAA0967691 and KAA0967692, respectively) and genes encoding enzymes related to the production of indole acetic acid (IAA), i.e., indolepyruvate ferredoxin oxidoreductase (EC: 1.2.7.8) (GenBank accession number: KAA0975498) and cellulose degradation, i.e., beta-glucosidase (EC: 3.2.1.21) (GenBank accession number: KAA0973975). Importantly, ANT_H12B encodes the *pvd* genes, which are responsible for the biosynthesis of pyoverdine siderophore (Figure 2; Table 2). Moreover, the clustered *acs* genes involved in biosynthesis of the secondary siderophore, achromobactin, were also identified, as well genes encoding achromobactin permease (GenBank accession number: KAA0975730) and ABC transporters (GenBank accession numbers: KAA0975548, KAA0975549, KAA0975550, KAA0975551 and KAA0975552) (Figure 2; Table 2).

**TABLE 2:** Siderophore and biosurfactant biosynthesis genes encoded by ANT_H12B, ANT_H59 and ANT_WA51. NCBI—National Center for Biotechnology Information; AA—amino acids.

Strain	Metabolite	GenBank accession number	Gene	Encoded protein	NCBI Reference protein	AA similarity (%)
ANT_H12B	pyoverdine	KAA0974491	*pvdH*	L-2,4-diaminobutyrate:2-ketoglutarate 4-aminotransferase	WP_063341054	96.60
		KAA0974500	*pvdL*	Chromophore precursor synthetase	WP_056742390	94.48
		KAA0974501	*pvdS*	Sigma factor	WP_008145913	99.45
		KAA0974502	*pvdY*	Acetyltransferase	WP_059402349	88.72
		KAA0961748	*pvdQ*	Acyl-homoserine lactone acylase	WP_046047187	93.21
		KAA0959584	*pvdE*	Synthetase	WP_095131071	98.18
		KAA0959585	*pvdO*	Chromophore maturation protein	WP_095131073	97.26
		KAA0959586	*pvdN*	Aminotransferase	WP_134103203	92.96
		KAA0959587	*pvdM*	Dipeptidase	WP_008152233	98.44
		KAA0959588	*pvdP*	Synthetase	WP_056741954	95.92
		KAA0959593	*pvdA*	l-ornithine 5-monooxygenase	WP_150656455	98.88
ANT_H12B	achromobactin	KAA0975552	*acsA*	Synthetase	WP_123512555	92.48
		KAA0975553	*acsB*	Aldolase	WP_185066905	94.94
		KAA0975554	*acsC*	Synthetase	WP_059404423	95.17
		KAA0975555	*acsE*	Decarboxylase	WP_054052959	95.29
		KAA0975556	*acsD*	Synthetase	WP_213936767	96.46
		KAA0975731	*acsF*	Aminotransferase	WP_046054252	97.16
ANT_H59	vibrioferrin	KAA0939502	*pvsA*	Ligase/carboxylase	WP_011513612	98.30
		KAA0939503	*pvsB*	Amide bond forming protein	ABE75059	92.69
		KAA0939504	*pvsC*	Membrane-spanning transport protein	WP_149491587	97.37
		KAA0939505	*pvsD*	Amide bond forming protein	WP_201551309	96.33
		KAA0939506	*pvsE*	Decarboxylase	WP_011513608	95.49
ANT_WA51	bacillibactin	KAA0933946	*dhbF*	2,3-dihydroxybenzoate-glycine-threonine trimeric ester synthetase	WP_213418887	99.66
		KAA0933947	*dhbB*	Isochorismatase	WP_070547710	99.36
		KAA0933948	*dhbE*	2,3-dihydroxybenzoate-AMP ligase	WP_029725883	99.81
		KAA0933949	*dhbC*	Isochorismate synthase	WP_213413020	99.75
		KAA0933950	*dhbA*	2,3-dihydro-2,3-dihydroxybenzoate dehydrogenase	WP_031315322	99.62
ANT_WA51	fengycin	KAA0937135	*fenE*	Synthetase E	CAF1785475	99.92
		KAA0937136	*fenD*	Synthetase D	WP_213413123	99.94
		KAA0937137	*fenC*	Synthetase C	WP_195727836	99.92
		KAA0937138	*fenB*	Synthetase B	WP_015714035	99.96
		KAA0937139	*fenA*	Synthetase A	WP_160214989	98.40
ANT_WA51	surfactin	KAA0933188	*srfAD*	Synthase subunit 4	WP_003234568	99.59
		KAA0933189	*srfAC*	Synthase subunit 3	WP_213418956	99.92
		KAA0933190	*srfAB*	Synthase subunit 2	WP_182932206	99.97
		KAA0933191	*srfAA*	Synthase subunit 1	WP_182932205	99.97

In the genome of *Psychrobacter* sp. ANT_H59, we identified clustered genes encoding three main subunits of urease (EC 3.5.1.5), i.e.: alpha (GenBank accession numbers: KAA0939809, KAA0939890), beta (GenBank accession number: KAA0939891) and gamma (GenBank accession numbers: KAA0939892, KAA0939808), as well as the cluster of *pvs* genes involved in vibrioferrin siderophore biosynthesis (Figure 2; Table 2).

In-depth analysis of the ANT_WA51 genome revealed the presence of genes related to cellulolytic—i.e. beta-1,4-glucanase (EC 3.2.1.4) (GenBank accession number: KAA0937117), 6-phospho-beta-glucosidase (EC 3.2.1.86) (GenBank accession numbers: KAA0932637, KAA0932987, KAA0933198) and ureolytic—i.e., alpha, beta and gamma urease subunits (GenBank accession numbers: KAA0934394, KAA0934395, KAA0934396, respectively) activities. Moreover, ANT_WA51 encodes genes responsible for synthesis of bacilysin, i.e.,: *bacE*, *bacD*, *bacC*, *bacB*, *bacA* (GenBank accession numbers: KAA0932482, KAA0932483, KAA0932484, KAA0932485 and KAA0932486, respectively), as well as many other biotechnologically important enzymes such as levanase (EC 3.2.1.65) (GenBank accession number: KAA0937826), pullulanase (EC 3.2.1.41) (GenBank accession number: KAA0938134), arabinogalactan endo-1,4-beta-galactanase (EC 3.2.1.89) (GenBank accession number: KAA0934156), neopullulanase (EC 3.2.1.135) (GenBank accession number: KAA0934202), endo-beta-1.3–1.4 glucanase (licheninase) (EC 3.2.1.73) (GenBank accession number: KAA0932620), glucuronoarabinoxylan endo-1,4-beta-xylanase (EC 3.2.1.136) (GenBank accession number: KAA0937119), arabinoxylan arabinofuranohydrolase (EC 3.2.1.55) (GenBank accession number: KAA0937120), endo-1,4-beta-xylanase (EC 3.2.1.8) (GenBank accession number: KAA0937190) and chitosanase (EC 3.2.1.132) (GenBank accession number: KAA0937810).

Importantly, in the genome of ANT_WA51, there are clusters of genes responsible for the formation of two different biosurfactants, i.e. fengycin and surfactin (Figure 2; Table 2). Moreover, in this genome, genes involved in catecholate siderophore—bacillibactin—biosynthesis were found (Figure 2; Table 2).

**FIGURE 3:**
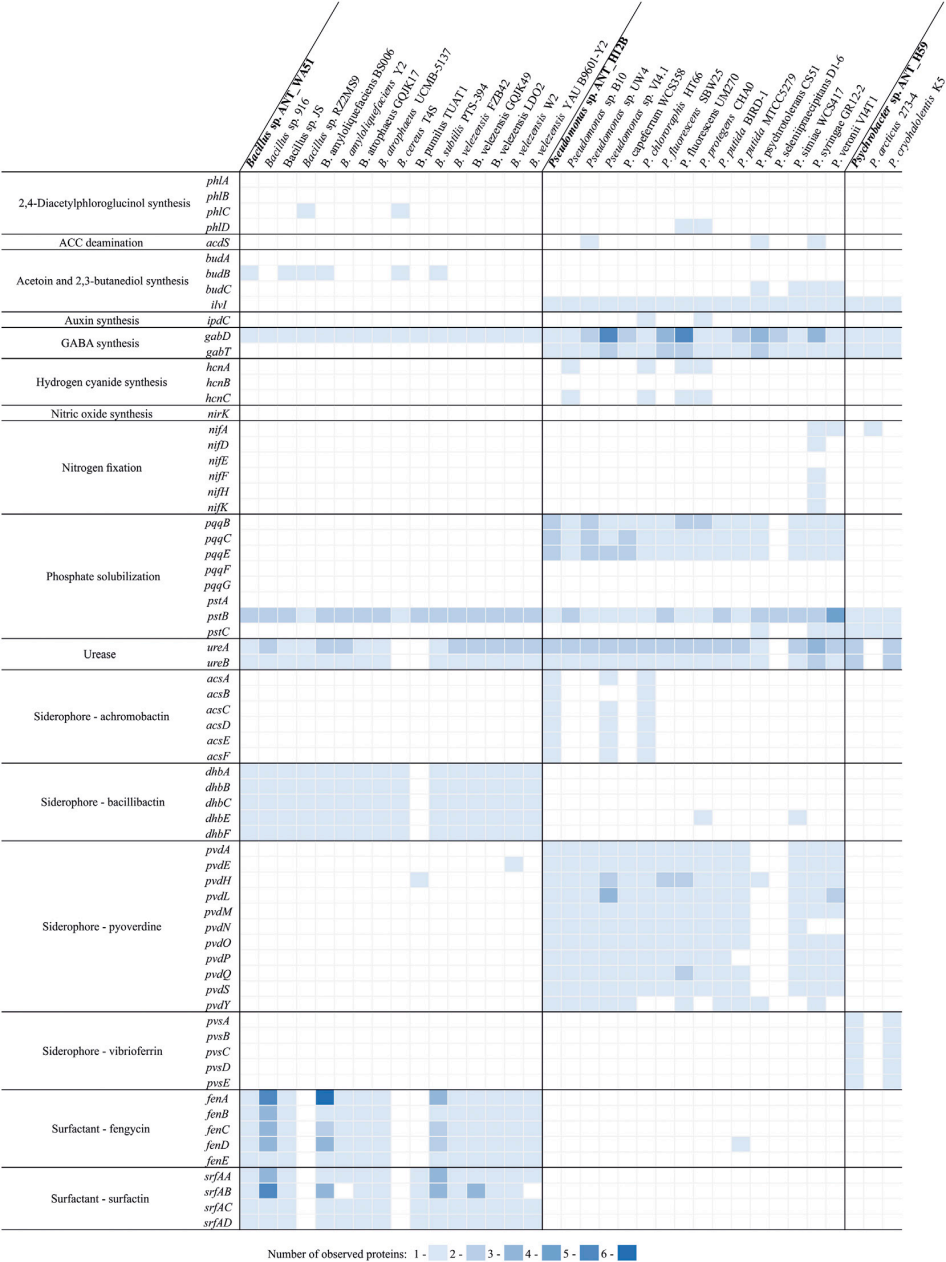
Distribution of various plant growth promoting traits in the proteomes of efficient plant growth promoters of the genus *Bacillus* and *Pseudomonas*, as well as the reference *Psychrobacter* strains.

For the analysis of the distribution of plant growth promoting traits in various efficient plant growth promoters belonging to the same genera as strains investigated in this study, a comparative analysis of proteomes of ANT_H12B and ANT_WA51 with 15 strains belonging to respective taxa was performed. In case of *Psychrobacter* sp. ANT_H59 there was no genome/proteome for a plant growth promoter available, thus, for the analysis two reference type-strains were used, i.e. *Psychrobacter arcticus* 273–4 and *Psychrobacter cryohalolentis* K5 (Figure 3). Analysis revealed that only two *Pseudomonas* strains, i.e. ANT_H12B and HT66 have complete sets of genes needed for achromobactin (secondary siderophore) production, however ANT_H12B have more copies of genes (namely *pqqB*, *pqqC* and *pqqE*) encoding proteins involved in phosphate solubilization (Figure 3). In case of compared *Bacillus* spp., no unique features of the ANT_WA51 strain were observed. Interestingly, it was also revealed that the content of plant growth promoting traits in *Psychrobacter* sp. ANT_H59 and *P. cryohalolentis* K5 is identical (Figure 3), but to our best knowledge the later strain was not tested for its ability to promote plant growth.

### Effect of Bacteria and Their Metabolites on Plant Growth

The main aim of the research was to test the effect of soil bioaugmentation with Antarctic strains, as well as the influence of their metabolites (including siderophores and biosurfactants) on plant (alfalfa) growth promotion. For this purpose, a complex experiment was performed with eight of the tested experimental variants including: 1) bioaugmentation with ANT_H12B, ANT_H59 or ANT_WA51 strains; 2) soil supplementation with bacterial metabolites originated from each strain; and 3) two adequate controls (i.e., GASN medium and 0.85% (w/v) saline solution). The measurements were performed every 7 days for 3 weeks.

**FIGURE 4:**
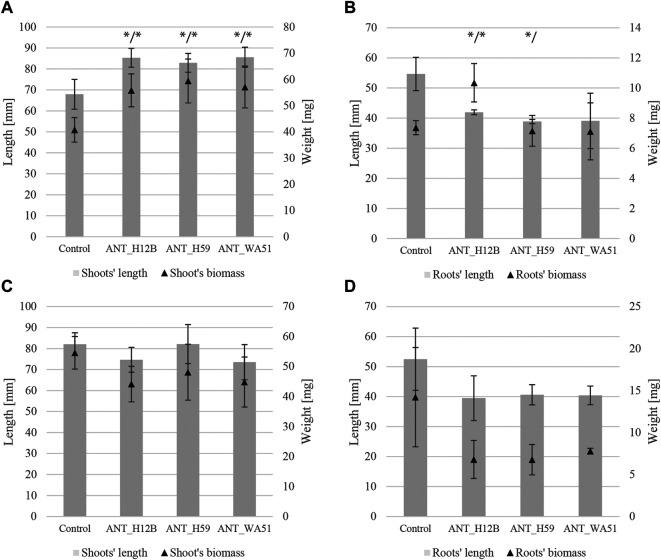
Length and biomass of shoots and roots of plants after 21 days of the experiment. Panels **(A,B)** refer to soil bioaugmentation with bacteria; **(C,D)** to the application of their metabolites. Error bars represent standard deviations. */* - means statistical significance (*p* < 0.05) for length and biomass; */means statistical significance (*p* < 0.05) for length only.

**FIGURE 5:**
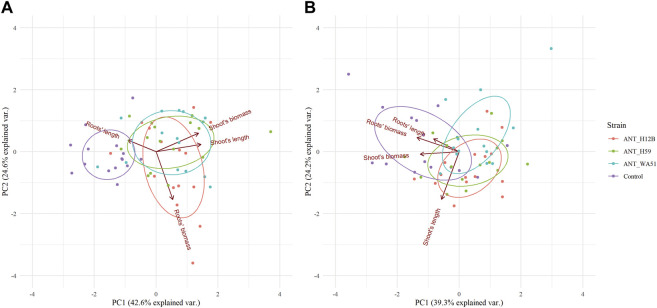
Principal component analysis (PCA) of plant morphological features in reponse to: **(A)** inoculation with various tested bacteria; **(B)** supplementation with bacterial metabolites after 21 days of the experiment. Particular bacterial strains used for inoculation (panel **(A)**) or strain-specific metabolites (panel **(B)**) are represented by unified color code. PCA eigenvectors on both panels indicate the influence of each variable on principal components.

Bioaugmentation with bacteria after 21 days of experimentation resulted in significant changes in the length of plants shoots and fresh root biomass. It was shown that treating soil with ANT_H12B, ANT_H59 and ANT_WA51 increased shoots’ length by 26, 22 and 26% and shoots’ fresh biomass by 37, 46 and 40%, respectively, compared to plants grown in non-inoculated soil (Figure 4). The increase in roots’ fresh biomass was observed only in plants cultivated in soil bioaugmented with the ANT_H12B strain, which resulted in a 40% increase compared to the control (Figure 4). However, both ANT_H12B and ANT_H59 decreased the roots’ length by 23 and 29%, respectively. Observed results were also reflected in principal component analysis (PCA) (Figure 5A). Two main components accounted for 67.2% of the variability observed in the data, with PC1 accounting for 42.6% of the total variation and PC2 accounting for 24.6%. PCA indicated positive correlations between inoculation with all tested strains and shoots’ biomass and length as well as roots’ biomass, especially for ANT_H12B (Figure 5A). In case of application of metabolites produced by tested strains, no transparent and statistically significant effect was observed (Figures 4, 5B).

All analyzed strains were efficient producers of siderophores. Thus, to investigate the potential effect of siderophores on plant growth promotion, the iron content of alfalfa’s roots and shoots was measured. It was shown that inoculation of soil with the ANT_H12B strain, after 21 days, increased the amount of iron in the roots by 38%. No statistically significant changes were observed when the sole metabolites produced by the strains were used (Table 3).

**TABLE 3:** Iron content (µg/mg) in shoots and roots of plants after bioaugmentation with bacteria or application of metabolites. Presented results refer to the 21st day of the experiment. * — means statistical significance *p* < 0.05 compared to control.

	Bacteria				Metabolites			
	Control	ANT_H12B	ANT_H59	ANT_WA51	Control	ANT_H12B	ANT_H59	ANT_WA51
**Shoots**	0.136	0.109	0.123	0.135	0.146	0.101	0.104	0.132
**Roots**	0.989	1.137*	0.903	1.366	1.300	1.073	0.954	0.790

### The Influence of Bacteria and Their Metabolites on the Physical and Chemical Properties of Soil and Soil Microbiome Composition

**FIGURE 6:**
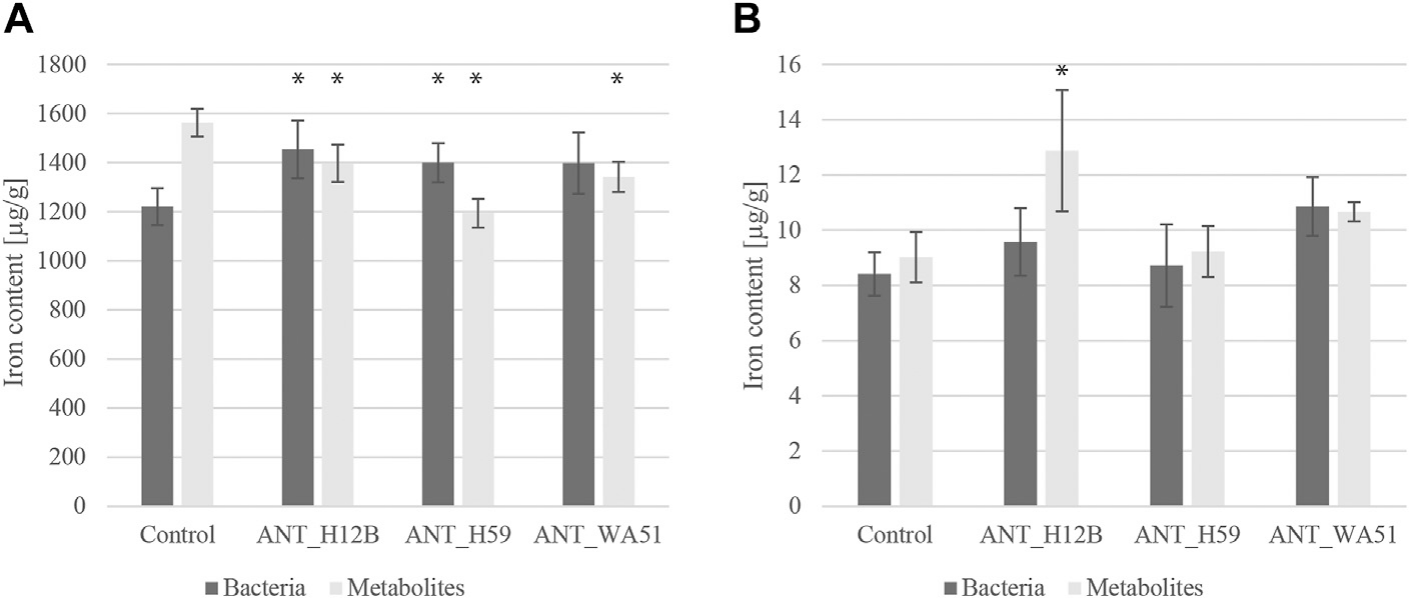
Total **(A)** and bioavailable **(B)** iron content in soil after 21 days of the experiment. Error bars represent standard deviations. * —means statistical significance *p* < 0.05 compared to control.

Measuring soil pH and redox potential revealed that both bioaugmentation with bacteria and supplementation with their metabolites had a negligible effect on the general physicochemical conditions of the soil since no statistically significant changes were observed (Supplementary Table S1). Further soil analyses concerned the influence of bacteria and their metabolites on the abundance and bioavailability of iron in the soil. The measurement of the total iron content showed that the addition of the metabolites produced by ANT_H12B, ANT_H59 and ANT_WA51 reduced the total iron content in the soil after 21 days by 11, 24 and 14%, respectively. However, the analysis of iron bioavailability revealed a beneficial effect of supplementation with the metabolites of the ANT_H12B and ANT_WA51 strains. After 7, 14 and 21 days of the experiment, the addition of metabolites of ANT_H12B increased the bioavailability of iron in soil by 19, 30 and 40%, respectively, while the addition of metabolites of the ANT_WA51 increased it by 30, 36 and 18%, respectively. Moreover, bioaugmentation with *Psychrobacter* sp. ANT_H59, as well as with *Pseudomonas* sp. ANT_H12B, increased total iron content by 15 and 19%, respectively, after 21 days. The ANT_H12B strain also increased iron bioavailability by: 46% after 7 days, 28% after 14 days and 29% after 21 days of the experiment (Figure 6).

**FIGURE 7:**
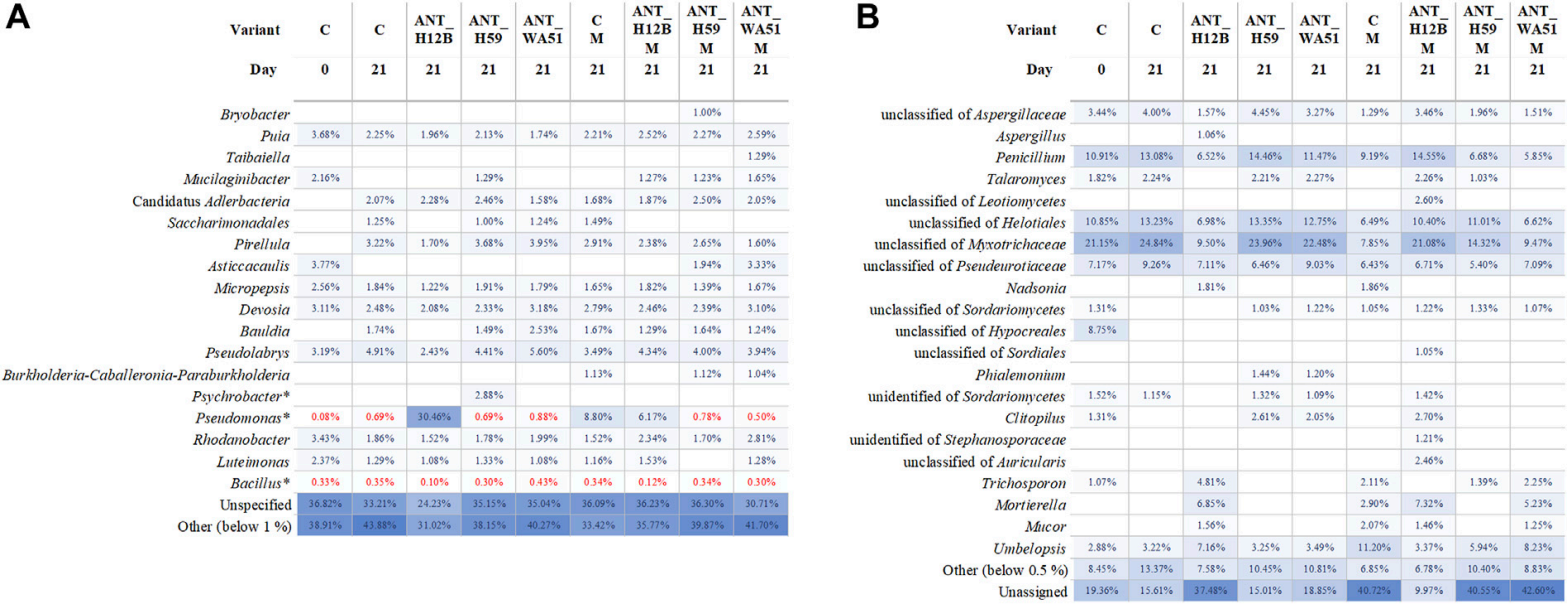
Bacterial **(A)** and fungal **(B)** diversity based on V3-V4 of 16S rRNA and ITS2 amplicons analysis. The diversity was presented on the genus level when possible, otherwise, the last specific taxon level name was used. For the clarity of the figure, only taxa with abundance over 1% or 0.5% for *Bacteria* and *Fungi*, respectively, are shown. Taxa with abundances below thresholds are presented as the “Other” group. ASVs that were not classified or were classified with not specific taxonomic names are presented as “Unassigned” or “Unspecified” groups, respectively. In the case of *Psychrobacter*, *Pseudomonas* and *Bacillus* genera, also the abundance below the threshold was shown and marked in red. In the variant description, “C” and “M” are abbreviations of control samples and metabolites used for supplementation, respectively.

The number of heterotrophic bacteria in the soil did not show significant differences after 7, 14 and 21 days of the experiment, both in soil treated with metabolites and bioaugmented with bacteria (Supplementary Figure S2). However, metagenomic analyses confirmed the presence of the tested strains in bioaugmented soils after 21 days of the experiment. What is important, it was established that bioaugmentation with the ANT_H12B, ANT_H59 and ANT_WA51 strains did not significantly change the composition of the bacterial and fungal microbiome in the soil but influenced the relative abundance of microorganisms (Figure 7). The most significant result is an increase in the content of pseudomonads in soil by 30%, which was coupled with bioaugmentation with the ANT_H12B strain (Figure 7).

## Discussion

Bacteria living in the soil play a fundamental role in the proper development of plants. Many PGP microorganisms can compensate nutrient deficiencies by producing multiple enzymes, secondary metabolites or performing biotransformations of other compounds into desired ones (Jabborova et al., 2020; Kalam et al., 2020; Akhtar et al., 2021; Hamid et al., 2021). In addition, some PGPB may also protect plants against fungal pathogens (Glick, 2012). However, common mesophilic bacteria indicate low activity during the late autumn, winter and early spring periods of temperate climatic zones (Mishra et al., 2010). In this study, we demonstrated the ability of Antarctic cold-active bacterial strains, that is, *Pseudomonas* ANT_H12B, *Psychrobacter* ANT_H59 and *Bacillus* sp. ANT_WA51, to promote plant growth.

Cold-active strains that promote plant growth are microorganisms especially desired in agriculture; thus, recently many scientists have been seeking out psychrotolerant PGPB (Verma et al., 2015). Strains analysed in this study exhibited these desired features. It was shown that ANT_H12B and ANT_WA51 were able to dissolve inorganic forms of phosphorus (thus increasing its bioavailability) and produce plant hormones (auxins). Moreover, both strains showed proteolytic and cellulolytic activities, which may significantly increase the bioavailability of organic carbon in soils (Utobo and Tewari, 2015).

The ability to break down lipids and produce biosurfactants is a desirable feature in agriculture. The presence of biosurfactants significantly improves the quality of soil by: facilitating bioremediation processes—including degradation of hydrophobic petroleum compounds and pesticides (Bustamante et al., 2012; Ahmad et al., 2018); improving wettability (Ahmad et al., 2018) and facilitating the colonisation of the rhizosphere by bacteria (Sachdev and Cameotra, 2013). Moreover, the hydrophobic compounds released from the soil through biosurfactant activity may serve as a source of nutrients for microorganisms and plants (Sachdev and Cameotra, 2013). Investigated Antarctic strains, namely ANT_H59 and ANT_WA51, decomposed lipids as well as efficiently produced biosurfactants. While little is known about the chemical nature of the biosurfactants produced by *Psychrobacter* species, the ability to produce lipopeptide biosurfactants is a common feature of *Bacillus* spp. (Ongena et al., 2007). *Bacillus* sp. ANT_WA51 constitutively produced at least two lipopeptide biosurfactants, i.e. surfactin and fengicin, which may contribute to the induced systemic resistance of plants (Ongena et al., 2007) and protection against fungal pathogens such as *B. cinerea* (Kefi et al., 2015), *Aspergillus* spp. (Farzaneh et al., 2016; Liu et al., 2020) or *Fusarium* spp. (Hu et al., 2007; Jiang et al., 2016).

An important feature of analysed strains is their ability to produce siderophores. Numerous studies (Verma et al., 2011; Meena et al., 2017; Pradesh et al., 2019) indicate the key role of such iron chelators in plant growth promotion. The siderophores produced by bacteria analysed in this study represented distinct types; for example, catechol bacillibactin produced by ANT_WA51, carboxylic vibrioferrin produced by ANT_H59 and mixed pyoverdine secreted by ANT_H12B. Genomic and biochemical analyses confirmed the presence of these groups of siderophores. In the genome of ANT_H12B, there were also genes conferring the production of achromobactin. However, as a secondary siderophore, its production occurs under very specific environmental conditions. Moreover, it has a lower affinity for iron and its quantity is usually negligible compared to pyoverdine (Owen and Ackerley, 2011; Schalk et al., 2020). Vibrioferrin is a siderophore often associated with the marine environment from where it was first isolated (Amin et al., 2009). However, the PGP properties of this siderophore produced by the soil bacterium *Azotobacter* sp. were also confirmed (Sumbul et al., 2020). One of the most specific features of vibrioferrin is its high photosensitivity and susceptibility to photolysis. Interestingly, after photolysis, this iron-complexed siderophore is imported faster and more efficiently by bacteria (Amin et al., 2009). The production of vibrioferrin by *Psychrobacter* sp. ANT_H59 may be related to high UV radiation in the Antarctic region and thus be a result of adaptation to extreme environments (Staehelin et al., 2001). While there are only single mentions of the plant growth-promoting abilities of vibrioferrin, bacillibactin and pyoverdin are much better recognised in this regard. Bacillibactin is a common siderophore among *Bacillus* species and have the strongest affinity for iron compared to any other natural siderophore. Such iron chelator has been reported to be an efficient plant growth-promoting and antifungal agent (Li et al., 2014; Nithyapriya et al., 2021). Many reports also indicate the plant beneficial properties of pyoverdine, such as: facilitating plant growth (Vansuyt et al., 2007), increasing chlorophyll production and total iron content in plants, enhancing the production of various pigments and anthocyanins in plants (Nagata et al., 2013) and fungicidal activity (Bakker, 2005; Cornelis and Matthijs, 2007; Sass et al., 2018).

One of the PGP features of siderophores is the ability to limit the growth of pathogens due to competition for iron in the environment (Scavino and Pedraza, 2013). Siderophores produced by mesophilic strains lose their activity at lower temperatures, whereas many fungal phytopathogens are still active. Thus, the application of siderophores as fungicidal agents faces some limitations. A possible solution is using thermostable siderophores produced by cold-active bacteria. So far, evidence exists of the successful use of cold-active, siderophore-producing strains against various fungal pathogens (Yadav et al., 2015; Yarzábal et al., 2018). In this study, it was revealed that Antarctic *Bacillus* sp. ANT_WA51 was effective against a wide spectrum of fungal pathogens, which suggests its huge potential in agriculture.

The primary function of siderophores is to increase the bioavailability of iron. Here, it was shown that supplementation with metabolites originating from all three strains resulted in a reduction of the total iron content in the soil, but only metabolites from ANT_H12B and ANT_WA51 significantly increased iron bioavailability. Lack of an increase in the case of ANT_H59 may be a consequence of the properties of the produced siderophore since vibrioferrin is a much weaker chelator than the other two (Amin et al., 2009). None of the supplementation variants increased the iron content in alfalfa’s roots. This phenomenon can be explained by the reduction of iron retention due to the action of siderophores, which could have resulted in its rinsing out of the pots. On the other hand, the increase in the total iron in the soil by bioaugmentation with *Psychrobacter* sp. ANT_H59 and *Pseudomonas* sp. ANT_H12B may be the result of bacterial activity and, thus, increased iron retention.

In the case of bacterial bioaugmentation, all investigated strains facilitated the growth of alfalfa’s shoots. It should be noted that only the ANT_H12B strain increased iron bioavailability in soil as well as biomass and iron content in the roots. Interestingly, the amount of bioavailable iron, as a result of bioaugmentation with ANT_H12B, decreased by almost two times after the first week and remained stable during subsequent weeks. The result correlates with the iron content in alfalfa’s roots, as plants bioaugmented with ANT_H12B had as much as 38% more iron content in their roots compared to the control. Furthermore, similarly to the application of ANT_H12B, bioaugmentation with *Psychrobacter* sp. ANT_H59, significantly reduced the length of alfalfa roots, but in this case, biomass remained similar to the control. This may mean that the plants bioaugmented with ANT_H59 or ANT_H12B strain did not have to invest in root length to search for nutrients (Atkinson et al., 2014).

In this study, the negligible effect of supplementation with metabolites and bioaugmentation with bacteria on the general physicochemical conditions of the soil was observed. It may be beneficial especially for plants with strict soil requirements. Similarly, metagenomic analyses confirmed that supplementation had only a limited effect on the structure of autochthonous microbiota in soil and, therefore, did not disturb the natural ecosystem. This is in line with the principles of sustainable agriculture, which assumes limiting the impact on soil biodiversity and maintaining natural and intact homeostasis in soil systems (Dobrovol’skaya et al., 2015).

## Conclusion

Three investigated psychrotolerant bacteria, that is, *Pseudomonas* sp. ANT_H12B, *Psychrobacter* sp. ANT_H59 and *Bacillus* sp. ANT_WA51, revealed plant growth-promoting properties. Functional analyses confirmed their abilities to: produce siderophores and biosurfactants; decompose lipids, proteins and cellulose; increase the availability of phosphorus; and production of plant hormones. *Bacillus* sp. ANT_WA51 also inhibited the growth of fungal pathogens. Genomic analyses revealed the presence of complete gene clusters responsible for synthesis of three different siderophores, that is, pyoverdine (*pvd*), vibrioferrin (*pvs*) and bacillibactin (*dhb*) in ANT_H12B, ANT_H59 and ANT_WA51, respectively. In the genome of *Bacillus* sp. ANT_WA51, genes involved in synthesis of surface-active compounds, including surfactin (*srf*), were also found. It was shown that all tested strains stimulated alfalfa growth, but ANT_H12B also increased iron content in roots as well as their biomass. What is more, the application of the ANT_H12B strain, its metabolites and metabolites from ANT_WA51 increased iron bioavailability in soil. Bioaugmentation with Antarctic bacteria did not affect the physicochemical conditions and had a negligible effect on the structure of the bacterial and fungal community in the soil.

## Data Availability

The datasets presented in this study can be found in online repositories. The names of the repository/repositories and accession number(s) can be found below: https://www.ncbi.nlm.nih.gov/, NZ_VOBI00000000 https://www.ncbi.nlm.nih.gov/, NZ_VOBE00000000 https://www.ncbi.nlm.nih.gov/, NZ_VOBB00000000.
