# Supplementary material for: Application of Psychrotolerant Antarctic Bacteria and Their Metabolites as Efficient Plant Growth Promoting Agents
Source: Front Bioeng Biotechnol. 2022 Feb 24;10:772891. doi: 10.3389/fbioe.2022.772891 (PMC8907978; doi:10.3389/fbioe.2022.772891)
Supplement: Supplementary file 1 [file DataSheet1.PDF]

Supplementary Material

1 Supplementary Figures

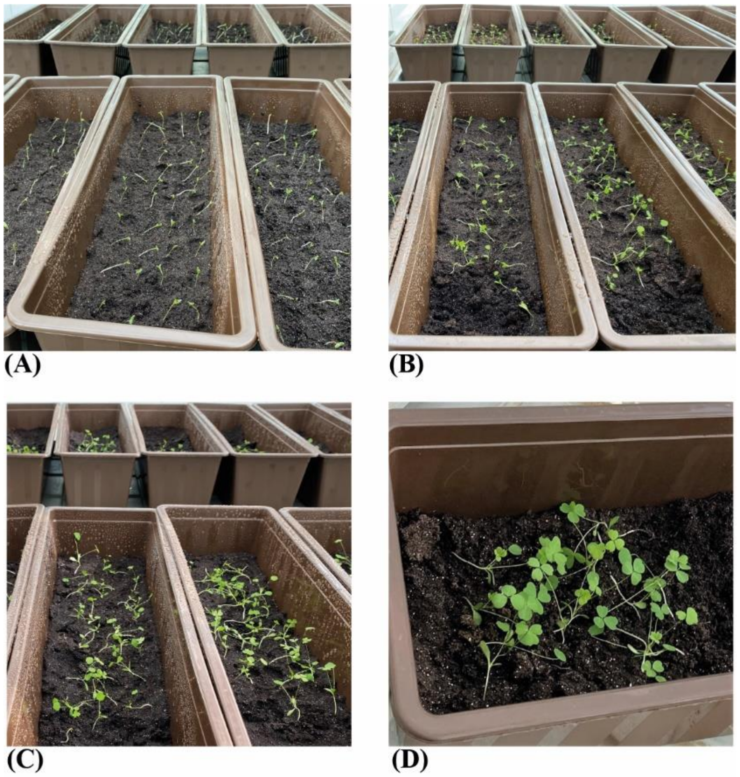

Supplementary Figure 1. Growth of alfalfa after: A) pre-cultivation and transplantation; B) 7 days; C) 14 days; D) 21 days of the experiment.

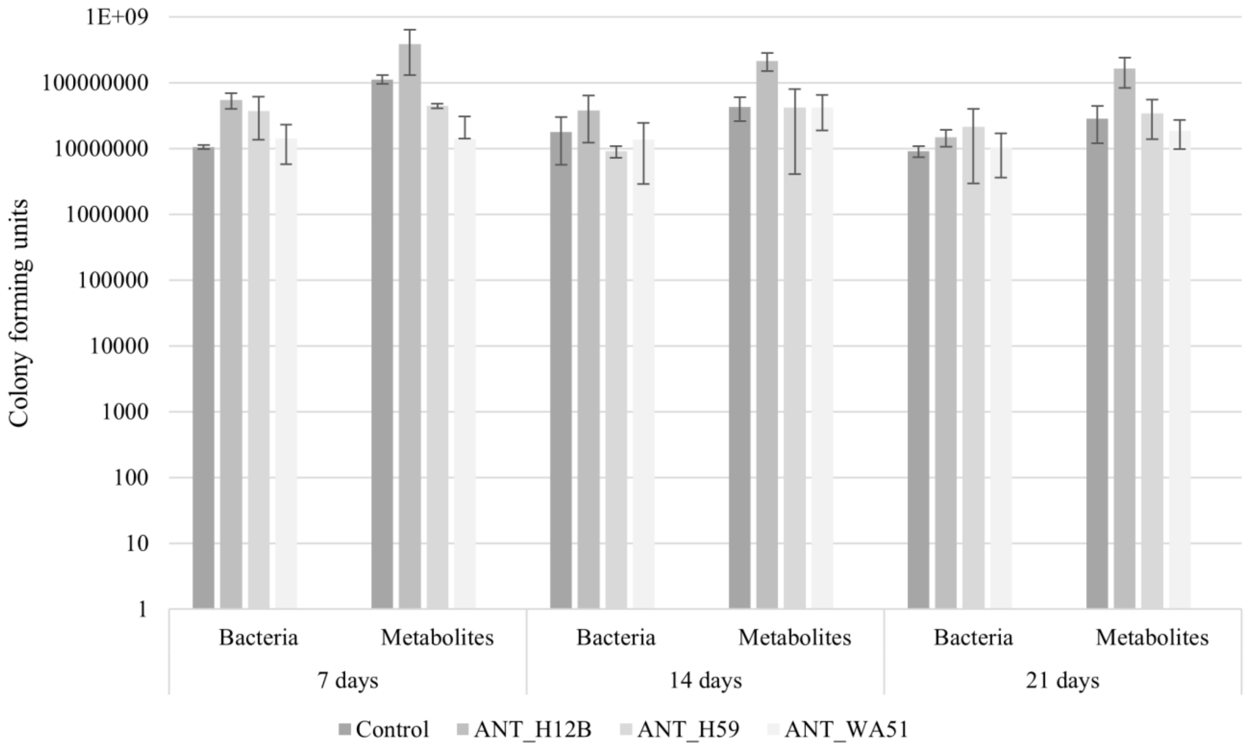

Supplementary Figure 2. Changes of the content of heterotrophic bacteria after consecutive weeks of experiment. Error bars represent standard deviations.

2 Supplementary Table

Supplementary Table 1. Changes of soil pH and redox potential after three consecutive weeks of experiment. Bacteria mean bioaugmentation with appropriate bacterial strains; metabolites mean application of their metabolites.

		pH			
		Control	ANT_H12B	ANT_H59	ANT_WA51
7 days	Bacteria	6.72	6.553333	6.676667	6.736667
	Metabolites	6.553333	6.406667	6.56	6.436667
14 days	Bacteria	6.746667	6.553333	6.623333	6.746667
	Metabolites	6.55	6.493333	6.6	6.366667
21 days	Bacteria	6.76	6.533333	6.556667	6.77
	Metabolites	6.606667	6.406667	6.543333	6.33
		Redox potential			
		Control	ANT_H12B	ANT_H59	ANT_WA51
7 days	Bacteria	19	29.33333	21.66667	18.33333
	Metabolites	27.66667	38	28.33333	36
14 days	Bacteria	18	28.66667	24.66667	18
	Metabolites	29	32.66667	26.33333	39.66667
21 days	Bacteria	11.33333	24.33333	23.66667	10.66667
	Metabolites	20	31.33333	23.66667	35.66667

3 Supplementary Data

Supplementary Data 1. Sequences of the reference bacterial enzymes/proteins involved in plant growth promotion used for the comparative proteomic analysis.

```
>Urease~ureA~WP_000779223
MKLTPKELDKLMLHYAGELAKKRKEKGIKLNYVEAVALISAHIMEEARAGKKTAAELMQE
GRTLLKPDDVMDGVASMIHEVGIEAMFPDGTKLVTVHTPIEANGKLVPGELFLKNEDITI
NEGKKAVSVKVKNVGDRPVQIGSHFHFFEVNRCLDFDREKTFGKRLDIASGTAVRFEPGE
EKSVELIDIGGNRRIFGFNALVDRQADNESKKIALHRAKERGFHGAKSDDNYVKTIKE
>Urease~ureB~WP_000724295
MKKISRKEYVSMYGPTTGDKVRLGDTDLIAEVEHDYTIYGEELKFGGGKTLREGMSQSNN
PSKEELDLIITNALIVDYTGIYKADIGIKDGKIAGIGKGGNKDMQDGVKNNLSVGPATEA
LAGEGLIVTAGGIDTHIHFISPQQIPTAFASGVTTMIGGGTGPADGTNATTITPGRRNLK
WMLRAAEEYSMNLGFLAKGNASNDASLADQIEAGAIGFKIHEDWGTTPSAINHALDVADK
YDVQVAIHTDTLNEAGCVEDTMAAIAGRTMHTFHTEGAGGGHAPDIIKVAGEHNILPAST
NPTIPFTVNTEAEHMDMLMVCHHLDKSIKEDVQFADSRIRPQTIAAEDTLHDMGIFSITS
SDSQAMGRVGEVITRTWQTADKNKKEFGRLKEEKGDNDNFRIKRYLSKYTINPAIAHGIS
EYVGSVEVGKVADLVLWSPAFFGVKPNMIIKGGFIALSQMGDANASIPTPQPVYYREMFA
HHGKAKYDANITFVSQAAYDKGIKEELGLERQVLPVKNCRNITKKDMQFNDTTAHIEVNP
ETYHVFVDGKEVTSKPANKVSLAQLFSIF
>Phosphate_solubilization~pqqB~WP_002113742
MYIYVLGSAAGGGFPQWNCNCPNCHGVRTGTIQAKARTQSSIAVSENGTDWVLLNASPDI
RQQLFEFKAAQPARKLRDTGIISVILMDSQLDHTTGLLTLREGCPMNVWCTEMVHQDLTN
GFPVFNMLKHWNGGLQYHEINPKQAFKIDGFENLEFLPLIIKSAAPPYSPHRNNPHDGDN
IALIIKDHKTQKQLFYAPGLGKIDDQIMQIMQSSDCVMIDGTLWTDDEMQQTGVGTKTGR
EMGHLYISGEGGSLSYLNKLSTPKKVLIHINNTNPILNENSSQFAELKANGVEVAYDGMQ
IEL
>Phosphate_solubilization~pqqC~WP_004225008
MLITDTLSPQAFEEALRAKGAFYHIHHPYHIAMHNGDATRKQIQGWVANRFYYQTTIPLK
DAAIMANCPDAQTRRKWVQRILDHDGSHGEDGGIEAWLRLGEAVGLSRDDLLSERHVLPG
VRFAVDAYLNFARRACWQEAACSSLTELFAPQIHQSRLDSWPQHYPWIKEEGYFYFRSRL
SQANRDVEHGLALAKAYCDSAEKQNRMLEILQFKLDILWSMLDAMTMAYALQRPPYHTVT
DKAAWHTTRLV
>Phosphate_solubilization~pqqE~WP_002113586
MTEGVGLPLWLLAELTYRCPLQCPYCSNPLDYAQHKNELTTQEWFDVFDQARQMGAVQLG
FSGGEPLVRQDLEQLVAHAHQQGFYTNLITSGMGLTEQRIADLKQAGLDHIQVSFQASDP
VVNDALAGSKHAFEQKYEMCRLVKKYDYPMVLNFVIHRHNIDQIEQIIELCLELNADTVE
LAICQFYGWAFLNRQGLLPTQEQLTRAERITNEYREKLKAQNHPCKLIFVVPDYYEERPK
ACMNGWGKIFFTVAPDGMALPCHAARQLPISFPNVREHKLSDIWYKSTGFNHFRGDAWMP
EGCRSCPDKDRDFGGCRCQAYMLTGDAANADPVCGKSPYHQMIEQARAESQLVAPLQNLV
FRNSRNSKSLSATQNIPVHTITDI
>Phosphate_solubilization~pqqF~WP_004225013
MTLATRTVTLPGGLQATLVHQPQADRAAALARVAAGSHHEPSRFPGLAHLLEHLLFYGGE
RYQDDDRLMGWVQRQGGSVNATTLARHSAFFFEVAADALADGVARLQEMLQAPLLLREDI
QREVAVIDAEYRLIQQHEPSRREAAVRHAASAPAAFRRFQVGSADALAGDLAALQAALGD
FHRTHYVARRMQLWLQGPQSLEALGELAARFAAGLAAGEAPPPAPPLRLGEFTALQLAVS
SQPALWRCPLIALSDNVTLLREFLLDEAPGSLMAGLRQRRLAGDVALNWLYQDRHLGWLA
LVFASDRPEEVDRQITHWLQALQQTTPEQQQHYYQLSRRRFQALSPLDQLRQRAFGFAPG
APPAGFADFCAALQVAPSVSLACQTVSPGEPVATQGFSLPLSRWRRRPESDPALAFAFYP
QAAGDLVAKCPEKAAPLLHLPSPGDPPRLLLRPPFYCSPDQAEGLARGEQLRPLLAALRH
AGGHGEWHLFDGSWQLTLQLPEPGRRPEAILQAILRQLALPVASLTPSPESIAIRHLMAQ
LPERLGTSGHQKGWLAALAGGSAEDAQWVARQLSLITAPVNPPMPAPAPCRRGVERLVYP
GGDTALLVFIPLPDGASLAALRLLAQHCEPLFFQRLRVEQQIGYVVSCRYQRVADRDGLL
MALQSPDRRAGELLRCGKDFLRQLAPMDEATFRPLQQRLAAQIRASRPPEARALSALRQE
YGLPELTPQAVDALRVAEVADLAREMTRRRRRWQVLFTTGD
>Phosphate_solubilization~pqqG~WP_003597599
```

MHVVILGSAAGGGVPQWNCRCSICSLAWAGDSRVRPRTQSSIAVSPDGERWLLLNASPDI
RQQIQANPQMHPREGLRHSPIHAVLLTNGDVDHVAGLLTLREGQPFTLYATPGILASVSD
NRVFDVMAADVVKRQTIALNETFEPVPGLSVTLFSVPGKVPLWLEDASMEIGAETETTVG
TMIEAGGKRLAYIPGCARVTEDLKARIAGADALLFDGTVLEDDDMIRAGVGTKTGWRMGH
IQMNGETGSIASLADIEIGRRVFVHINNTNPVLIEDSYERASVEARGWTVAHDGLTLDL
>Phosphate_solubilization~pstA~WP_001251998
MAMVEMQTTAALAESRRKMQARRRLKNRIALTLSMATMAFGLFWLIWILMSTITRGIDGM
SLALFTEMTPPPNTEGGGLANALAGSGLLILWATVFGTPLGIMAGIYLAEYGRKSWLAEV
IRFINDILLSAPSIVVGLFVYTIVVAQMEHFSGWAGVIALALLQVPIVIRTTENMLKLVP
YSLREAAYALGTPKWKMISAITLKASVSGIMTGILLAIARIAGETAPLLFTALSNQFWST
DMMQPIANLPVTIFKFAMSPFAEWQQLAWAGVLIITLCVLLLNILARVVFAKNKHG
>Phosphate_solubilization~pstB~EIG5157684
MSMVETAPSKIQVRNLNFYYGKFHALKNINLDIAKNQVTAFIGPSGCGKSTLLRTFNKMF
ELYPEQRAEGEILLDGDNILTNSQDIALLRAKVGMVFQKPTPFPMSIYDNIAFGVRLFEK
LSRADMDERVQWALTKAALWNETKDKLHQSGYSLSGGQQQRLCIARGIAIRPEVLLLDEP
CSALDPISTGRIEELITELKQDYTVVIVTHNMQQAARCSDHTAFMYLGELIEFSNTDDLF
TKPAKKQTEDYITGRYG
>Phosphate_solubilization~pstC~WP_000741620
MAATKPAFNPPGKKGDIIFSVLVKLAALIVLLMLGGIIVSLIISSWPSIQKFGLAFLWTK
EWDAPNDIYGALVPIYGTLVTSFIALLIAVPVSFGIALFLTELAPGWLKRPLGIAIELLA
AIPSIVYGMWGLFIFAPLFAVYFQEPVGNIMSNIPIVGALFSGPAFGIGILAAGVILAIM
IIPYIAAVMRDVFEQTPVMMKESAYGIGCTTWEVIWRIVLPFTKNGVIGGIMLGLGRALG
ETMAVTFIIGNTYQLDSASLYMPGNSITSALANEFAEAESGLHVAALMELGLILFVITFI
VLAASKFMIMRLAKNEGAR
>2,4-Diacetylphloroglucinol_synthesis~phlA~P18952
MSMPLSFTSAVSPVAAIPTPRAAAETRTAASLRHAGKSGPVASPSQNTLNAQNLLNTLVG
DISAAAPTAAAAPGVTRGQQSQEGDYALALLAKDVYSLNGQGAAGFNRLSDSALLGFGID
PASLHDAGSGFQAGIYSNDKQYVLAFAGTNDWRDWLSNVRQATGYDDVQYNQAVAAAKSA
KAAFGDALVIAGHSLGGGLAATAALATGTVAVTFNAAGVSDYTLNRLGIDPAAAKKDAEA
GGIRRYSEQYDMLTSTQESTSLIPDAIGHNITLANNDTLTGIDDWRPSKHLDRSLTAHGI
DKVISSMAEQKPWEAKANA
>2,4-Diacetylphloroglucinol_synthesis~phlB~P18954
MPEGRRLRRALAIALLALVAVTGLLMMAKEQQMGQEISPFDGHSNLALAQAVARGDTQGI
HAQATQDRLRERGDRQVTLLQWAVLSQQPDSVQALLDLGADPAAAGLDGNSALHTAAMLQ
DAQYLRLLLAEGAQMNVRNAVTGATPLAAAVLAGREEQLRLLLAAGADTTLSDRLGDTPL
HLAAKINRRTWRCCCCRPGPMPGRATSRASRSSFTSRKRRRICRMTN
>2,4-Diacetylphloroglucinol_synthesis~phlC~WP_000731014
MKKKVLALAAAITVVAPLQSVAFAHENDGGSKIKIVHRWSAEDKHKEGVNSHLWIVNRAI
DIMSRNTTLVKQDRVAQLNEWRTELENGIYAADYENPYYDNSTFASHFYDPDNGKTYIPF
AKQAKETGAKYFKLAGESYKNKDMKQAFFYLGLSLHYLGDVNQPMHAANFTNLSYPQGFH
SKYENFVDTIKDNYKVTDGNGYWNWKGTNPEEWIHGAAVVAKQDYSGIVNDNTKDWFVKA
AVSQEYADKWRAEVTPMTGKRLMDAQRVTAGYIQLWFDTYGDR
>2,4-Diacetylphloroglucinol_synthesis~phlD~WP_011064130
MSTLCLPHVMFPQHKITQQQMVDHLENLHADHPRMALAKRMIANTEVNERHLVLPIDELA
VHTGFTHRSIVYEREARQMSSAAARQAIENAGLQISDIRMVIVTSCTGFMMPSLTAHLIN
DLALPTSTVQLPIAQLGCVAGAAAINRANDFARLDARNHVLIVSLEFSSLCYQPDDTKLH
AFISAALFGDAVSACVLRADDQAGGFKIKKTESYFLPKSEHYIKYDVKDTGFHFTLDKAV
MNSIKDVAPVMERLNYESFEQNCAHNDFFIFHTGGRKILDELVMHLDLASNRVSQSRSSL
SEAGNIASVVVFDVLKRQFDSNLNRGDIGLLAAFGPGFTAEMAVGEWTA
>Hydrogen_cyanide_synthesis~hcnA~WP_003113684
MHLLERQHDIQPLSRADMTIHLNGQPVAAAAGETVLNVLNAVGLRRLARNDHGQASGAFC
GMGVCHCCLVAIDGRPKRRACQTVVRPGMRVETESNRFDQEERP
>Hydrogen_cyanide_synthesis~hcnB~WP_003113685
MNLRPVIVGGGSAGMAAAIELARRGVPCVLFDEASRPGGVVYRGPLRAGVDPAYLGARYT
RMLEKLRRDFSACAGHIDLRLNSRVVGGDGQRLMVLDEAERLHEVEYSHLLLATGCHERS
VPFPGWTLPGVMLLGGLQLQIKSGVVKPLGDTLIAGSGPLLPLVACQLHAAGVRVAGVYE
ACAFGRMARESLALLNKPQLFLDGLSMLGYLKLNGIPLHYGWGVVEASGDGELTEVTVAP
YDEEWRPDLENARPVKASTLAVGYGFIPRTQLSQQLGLEHGFSDDGYLRAECNVWQQSSQ
PHIHLAGDMAGIRGGEAAMIGGRIAALSILLQREAIAPAEAIERRESHLARLEAIKRFRA

GVERYTQRGARQVELARADTVICRCEQVTRGDIERALEQGVQDIAGLKMRTRAGMGDCQG
RMCIGYCSDRLRRATGRHDVGWLRPRFPIDPIPFSAFQNLGTEA
>Hydrogen_cyanide_synthesis~hcnC~WP_003113686
MNRTYDIVIAGGGVIGASCAYQLSRRGNLRIAVVDDKRPGNATRASAGGLWAIGESVGLG
CGVIFFRMMSSRNRREAQGAAVAVDASTPHILPPAFFDLALQSNALYPELHRELIERHGM
DFKFERTGLKYVIQDDEDRQYAEHIVAQIPHLAEQVRWLDREELRRAEPAVSHAAHGALE
FLCDHQVSPFRLADAYLEAARQNGVELLPGTNVTGVLRQGRRISGVRTDNAGVLHCRTLI
NAAGAWAAELSEMATGRRIPVKPVKGQIVLTERMPRLLNGCLTTSDCYMAQKDNGEILIG
STTEDKGFDVSNTFPEIAGLVQGAVRCVPELQQVNLKRTWAGLRPGSPDELPILGPVAEV
EGYLNACGHFRTGILTSAITGVLLDRLVHEETLPLDIAPFLAARFQPEPAAVAVAAC
>Acetoin and 2,3-butanediol_synthesis~ilvI~WP_001295534
MEMLSGAEMVVRSLIDQGVKQVFGYPGGAVLDIYDALHTVGGIDHVLVRHEQAAVHMADG
LARATGEVGVVLVTSGPGATNAITGIATAYMDSIPLVVLSGQVATSLIGYDAFQECDMVG
ISRPVVKHSFLVKQTEDIPQVLKKAFWLAASGRPGPVVVDLPKDILNPANKLPYVWPESV
SMRSYNPTTTGHKGQIKRALQTLVAAKKPVVYVGGGAITAGCHQQLKETVEALNLPVVCS
LMGLGAFPATHRQALGMLGMHGTYEANMTMHNADVIFAVGVRFDDRTTNNLAKYCPNATV
LHIDIDPTSISKTVTADIPIVGDARQVLEQMLELLSQESAHQPLDEIRDWWQQIEQWRAR
QCLKYDTHSEKIKPQAVIETLWRLTKGDAYVTSDVGQHQMFAALYYPFDKPRRWINSGGL
GTMGFGLPAALGVKMALPEETVVCVTGDGSIQMNIQELSTALQYELPVLVVNLNNRYLGM
VKQWQDMIYSGRHSQSYMQSLPDFVRLAEAYGHVGIQISHPHELESKLSEALEQVRNNRL
VFVDVTVDGSEHVYPMQIRGGGMDEMWLSKTERT
>Acetoin and 2,3-butanediol_synthesis ~budA~WP_005311953
METHTQCDCAIEISQQFARWQARQGGGEVYQSSLMSALLAGVYEGETTMADLLRHGDFGL
GTFNRLDGELIAFERQIHQLKADGSARPAQAGQKTPFAVMTHFRPCLERRFDHPLSRDEI
HQWIDQLVGTDNVFVAFRLDGLFEQAQVRTVPCQTPPYKPMLEAIEAQPLFSFSQRRGTL
VGFRCPPFVQGINVAGYHEHFITEDRKGGGHILDYAMGHGQLQLSVVQHLNIELPRNPAF
QQADLNPADLDRAIRAAEG
>Acetoin and 2,3-butanediol_synthesis ~budB~WP_004175750
MDKQYPVRQWAHGADLVVSQLEAQGVRQVFGIPGAKIDKVFDSLLDSSIRIIPVRHEANA
AFMAAAVGRITGKAGVALVTSGPGCSNLITGMATANSEGDPVVALGGAVKRADKAKQVHQ
SMDTVAMFSPVTKYAVEVTAPDALAEVVSNAFRAAEQGRPGSAFVSLPQDVVDGPVSGKV
LPASGAPQMGAAPDDAIDQVAKLIAQAKNPIFLLGLMASQPENSKALRRLLETSHIPVTS
TYQAAGAVNQDNFSRFAGRVGLFNNQAGDRLLQLADLVICIGYSPVEYEPAMWNSGNATL
VHIDVLPAYEERNYTPDVELVGDIAGTLNKLAQNIDHRLVLSPQAAEILRDRQHQRELLD
RRGAQLNQFALHPLRIVRAMQDIVNSDVTLTVDMGSFHIWIARYLYSFRARQVMISNGQQ
TMGVALPWAIGAWLVNPERKVVSVSGDGGFLQSSMELETAVRLKANVLHLIWVDNGYNMV
AIQEEKKYQRLSGVEFGPMDFKAYAESFGAKGFAVESAEALEPTLRAAMDVDGPAVVAIP
VDYRDNPLLMGQLHLSQIL
>Acetoin and 2,3-butanediol_synthesis ~budC~WP_077313273
MSKVAMVTGGAQGIGRGISEKLAADGFDIAVADLPQQEEQAAETIKLIEAADQKAVFVGL
DVTDKANFDSAIDEAAEKLGGFDVLVNNAGIAQIKPLLEVTEEDLKQIYSVNVFSVFFGI
QAASRKFDELGVKGKIINAASIAAIQGFPILSAYSTTKFAVRGLTQAAAQELAPKGHTVN
AYAPGIVGTGMWEQIDAELSKINGKPIGENFKEYSSSIALGRPSVPEDVAGLVSFLASEN
SNYVTGQVMLVDGGMLYN
>Nitric_oxide_synthesis~nirK~WP_010660614
MAEQMQISRRTILAGAALAGALAPVLATTSAWGQGAVRKATAAEIAALPRQKVELVDPPF
VHAHSQVAEGGPKVVEFTMVIEEKKIVIDDAGTEVHAMAFNGTVPGPLMVVHQDDYLELT
LINPETNTLMHNIDFHAATGALGGGGLTEINPGEKTILRFKATKPGVFVYHCAPPGMVPW
HVVSGMNGAIMVLPREGLHDGKGKALTYDKIYYVGEQDFYVPRDENGKYKKYEAPGDAYE
DTVKVMRTLTPTHVVFNGAVGALTGDKAMTAAVGEKVLIVHSQANRDTRPHLIGGHGDYV
WATGKFNTPPDVDQETWFIPGGAAGAAFYTFQQPGIYAYVNHNLIEAFELGAAAHFKVTG
EWNDDLMTSVLAPSGT
>Auxin_synthesis~ipdC~WP_073396207
MRTPYCVADYLLDRLTDCGADHLFGVPGDYNLQFLDHVIDSPDICWVGCANELNASYAAD
GYARCKGFAALLTTFGVGELSAMNGIAGSYAEHVPVLHIVGAPGTAAQQRGELLHHTLGD
GEFRHFYHMSEPITVAQAVLTEQNACYEIDRVLTTMLRERRPGYLMLPADVAKKAATPPV
NALTHKQAHADSACLKAFRDAAENKLAMSKRTALLADFLVLRHGLKHALQKWVKEVPMAH

ATMLMGKGIFDERQAGFYGTYSGSASTGAVKEAIEGADTVLCVGTRFTDTLTAGFTHQLT
PAQTIEVQPHAARVGDVWFTGIPMNQAIETLVELCKQHVHAGLMSSSSGAIPFPQPDGSL
TQENFWRTLQTFIRPGDIILADQGTSAFGAIDLRLPADVNFIVQPLWGSIGYTLAAAFGA
QTACPNRRVIVLTGDGAAQLTIQELGSMLRDKQHPIILVLNNEGYTVERAIHGAEQRYND
IALWNWTHIPQALSLDPQSECWRVSEAEQLADVLEKVAHHERLSLIEVMLPKADIPPLLG
ALTKALEACNNA
>ACC_deamination~acdS~Q00740
MNLQRFPRYPLTFGPTPIQPLARLSKHLGGKVHLYAKREDCNSGLAFGGNKTRKLEYLIP
EALAQGCDTLVSIGGIQSNQTRQVAAVAAHLGMKCVLVQENWVNYSDAVYDRVGNIQMSR
ILGADVRLVPDGFDIGFRRSWEDALESVRAAGGKPYAIPAGCSDHPLGGLGFVGFAEEVR
AQEAELGFKFDYVVVCSVTGSTQAGMVVGFAADGRADRVIGVDASAKPAQTREQITRIAR
QTAEKVGLERDIMRADVVLDERFAGPEYGLPNEGTLEAIRLCARTEGMLTDPVYEGKSMH
GMIEMVRNGEFPEGSRVLYAHLGGVPALNGYSFIFRDG
>Nitrogen_fixation~nifA~P09570
MNATIPQRSAKQNPVELYDLQLQALASIARTLSREQQIDELLEQVLAVLHNDLGLLHGLV
TISDPEHGALQIGAIHTDSEAVAQACEGVRYRSGEGVIGNVLKHGNSVVLGRISADPRFL
DRLALYDLEMPFIAVPIKNPEGNTIGVLAAQPDCRADEHMPARTRFLEIVANLLAQTVRL
VVNIEDGREAADERDELRREVRGKYGFENMVVGHTPTMRRVFDQIRRVAKWNSTVLVLGE
SGTGKELIASAIHYKSPRAHRPFVRLNCAALPETLLESELFGHEKGAFTGAVKQRKGRFE
QADGGTLFLDEIGEISPMFQAKLLRVLQEGEFERVGGNQTVRVNVRIVAATNRDLESEVE
KGKFREDLYYRLNVMAIRIPPLRERTADIPELAEFLLGKIGRQQGRPLTVTDSAIRLLMS
HRWPGNVRELENCLERSAIMSEDGTITRDVVSLTGVDNESPPLAAPLPEVNLADETLDDR
ERVIAALEQAGWVQAKAARLLGMTPRQIAYRIQTLNIHMRKI
>Nitrogen_fixation~nifE~P08293
MKAKDIAELLDEPACSHNKKEKSGCAKPKPGATDGRCSFDGAQIALLPVADVAHIVHGPI
ACAGSSWDNRGTRSSGPDLYRIGMTTDLTENDVIMGRAEKRLFHAIRQAVESYLPPAVFV
YNTCVPALIGDDVDAVCKAAAERFGTPVIPVDSAGFYGTKNLGNRIAGEAMLKYVIGTRE
PDPLPVGSERPGIRVHDVNLIGEYNIAGEFWHVLPLLDELGLRVLCTLAGDARYREVQTM
HRAEVNMMVCSKAMLNVARKLQETYGTPWFEGSFYGITDTSQALRDFARLLDDPDLTART
EALIAREEAKVRAALEPWRARLEGKRVLLYTGGVKSWSVVSPLQDLGMKVVATGTKKSTE
EDKARIRELMGDDVKMLDEGNARVLLKTVDEYQADILIAGGRNMYTALKGRVPFLDINQE
REFGYGGYDRMLELVRHVCITLECPVWEAVRRPAPWDIPASQDARPSGGPFGER
>Nitrogen_fixation~nifF~WP_012698862
MAKIGLFFGSNTGKTRKVAKSIKKRFDDETMSDALNVNRVSAEDFAQYQFLILGTPTLGE
GELPGLSSDCENESWEEFLPKIEGLDFSGKTVALFGLGDQVGYPENYLDALGELYSFFKD
RGAKIVGSWSTDGYEFESSEAVVDGKFVGLALDLDNQSGKTDERVAAWLAQIAPEFGLSL
>Nitrogen_fixation~nifD~P07328
MTGMSREEVESLIQEVLEVYPEKARKDRNKHLAVNDPAVTQSKKCIISNKKSQPGLMTIR
GCAYAGSKGVVWGPIKDMIHISHGPVGCGQYSRAGRRNYYIGTTGVNAFVTMNFTSDFQE
KDIVFGGDKKLAKLIDEVETLFPLNKGISVQSECPIGLIGDDIESVSKVKGAELSKTIVP
VRCEGFRGVSQSLGHHIANDAVRDWVLGKRDEDTTFASTPYDVAIIGDYNIGGDAWSSRI
LLEEMGLRCVAQWSGDGSISEIELTPKVKLNLVHCYRSMNYISRHMEEKYGIPWMEYNFF
GPTKTIESLRAIAAKFDESIQKKCEEVIAKYKPEWEAVVAKYRPRLEGKRVMLYIGGLRP
RHVIGAYEDLGMEVVGTGYEFAHNDDYDRTMKEMGDSTLLYDDVTGYEFEEFVKRIKPDL
IGSGIKEKFIFQKMGIPFREMHSWDYSGPYHGFDGFAIFARDMDMTLNNPCWKKLQAPWE
ASEGAEKVAASA
>Nitrogen_fixation~nifH~WP_012698831
MAMRQCAIYGKGGIGKSTTTQNLVAALAEMGKKVMIVGCDPKADSTRLILHSKAQNTIME
MAAEAGTVEDLELEDVLKAGYGGVKCVESGGPEPGVGCAGRGVITAINFLEEEGAYEDDL
DFVFYDVLGDVVCGGFAMPIRENKAQEIYIVCSGEMMAMYAANNISKGIVKYANSGSVRL
GGLICNSRNTDREDELIIALANKLGTQMIHFVPRDNVVQRAEIRRMTVIEYDPKAKQADE
YRALARKVVDNKLLVIPNPITMDELEELLMEFGIMEVEDESIVGKTAEEV
>Nitrogen_fixation~nifK~WP_012698833
MSQQVDKIKASYPLFLDQDYKDMLAKKRDGFEEKYPQDKIDEVFQWTTTKEYQELNFQRE
ALTVNPAKACQPLGAVLCALGFEKTMPYVHGSQGCVAYFRSYFNRHFREPVSCVSDSMTE
DAAVFGGQQNMKDGLQNCKATYKPDMIAVSTTCMAEVIGDDLNAFINNSKKEGFIPDEFP
VPFAHTPSFVGSHVTGWDNMFEGIARYFTLKSMDDKVVGSNKKINIVPGFETYLGNFRVI
KRMLSEMGVGYSLLSDPEEVLDTPADGQFRMYAGGTTQEEMKDAPNALNTVLLQPWHLEK
TKKFVEGTWKHEVPKLNIPMGLDWTDEFLMKVSEISGQPIPASLTKERGRLVDMMTDSHT

```
WLHGKRFALWGDPDFVMGLVKFLLELGCEPVHILCHNGNKRWKKAVDAILAASPYGKNAT
VYIGKDLWHLRSLVFTDKPDFMIGNSYGKFIQRDTLHKGKEFEVPLIRIGFPIFDRHHLH
RSTTLGYEGAMQILTTLVNSILERLDEETRGMQATDYNHDLVR
>GABA_synthesis~gabT~WP_001087611
MNSNKELMQRRSQAIPRGVGQIHPIFADRAENCRVWDVEGREYLDFAGGIAVLNTGHLHP
KVVAAVEAQLKKLSHTCFQVLAYEPYLELCEIMNQKVPGDFAKKTLLVTTGSEAVENAVK
IARAATKRSGTIAFSGAYHGRTHYTLALTGKVNPYSAGMGLMPGHVYRALYPCPLHGISE
DDAIASIHRIFKNDAAPEDIAAIVIEPVQGEGGFYASSPAFMQRLRALCDEHGIMLIADE
VQSGAGRTGTLFAMEQMGVAPDLTTFAKSIAGGFPLAGVTGRAEVMDAVAPGGLGGTYAG
NPIACVAALEVLKVFEQENLLQKANDLGQKLKDGLLAIAEKHPEIGDVRGLGAMIAIELF
EDGDHNKPDAKLTAEIVARARDKGLILLSCGPYYNVLRILVPLTIEDAQIRQGLEIISQC
FDEAKQ
>GABA_synthesis~gabD~HBD1170345
MKLNDSNLFRQQALINGEWLDANNGEAIDVTNPANGDKLGSVPKMGADETRAAIDAANRA
LPAWRALTAKERATILRNWFNLMMEHQDDLARLMTLEQGKPLAEAKGEISYAASFIEWFA
EEGKRIYGDTIPGHQADKRLIVIKQPIGVTAAITPWNFPAAMITRKAGPALAAGCTMVLK
PASQTPFSALALAELAIRAGVPAGVFNVVTGSAGAVGNELTSNPLVRKLSFTGSTEIGRQ
LMEQCAKDIKKVSLELGGNAPFIVFDDADLDKAVEGALASKFRNAGQTCVCANRLYVQDG
VYDRFAEKLQQAVSKLHIGDGLDNGVTIGPLIDEKAVAKVEEHIADALEKGARVVCGGKA
HERGGNFFQPTILVDVPANAKVSKEETFGPLAPLFRFKDEADVIAQANDTEFGLAAYFYA
RDLSRVFRVGEALEYGIVGINTGIISNEVAPFGGIKASGLGREGSKYGIEDYLEIKYMCI
GL
>Siderophore_-_pyoverdine~pvdH~KAA0974491
MSVAISLIEDQPARIVPTPAETLYQFNESPLLARQSQQESNARSYPRRIPLALKRAKGIYVEDVEGRSFI
DCLAGAGTLALGHNHPVVIEAIQQVLTDELPLHTLDLTTPVKDQFVQDLFGLLPPALAAEAKIQFCGPTG
TDAVEAALKLVRTATGRSTVLSFQGGYHGMSQGALSLMGSLGPKKPLGALLSSGVQFMPYPYDYRCPFGL
GGAQGVQVNLNYLENLLNDPEAGVQLPAAVIVEAVQGEGGVIPADLDWLRGLRRITEKAGVALIVDEIQS
GFARTGKMFAFEHAGIIPDVVVMSKAIGGSLPLAVVVYRDWLDTWLPGAHAGTFRGNQMAMAAGSAVMRY
LTEHKVCEHAAAMGERLSEHLHILQRDFPQLGDIRGRGLMLGVELVDPTGAPDAQGHPPIFARLAPLVQR
ECLKRGLILELGGRHGGVVRFLPPLVITAAEIDRVADIFGRAMAAATASL
>Siderophore_-_pyoverdine~pvdL~KAA0974500
MTDAFELPSTLVQALQRRAALTPDRVALRFLAETEDQAVVLSYRELDQRARTIAGALQAEAAFGDRAVLL
FPSGPDYVAAFFGCLYAGVIAVPAYPPESARRHHQERLLSIISDAEPRLLLTSAGLRDALQQIEGAPPLL
CVDTLDSALAERWVAPNLADDHIAFLQYTSGSTALPKGVQVSHGNLVANELLIRHGFGIDLNPDDVIVSW
LPLYHDMGLIGGLLQPIFSGVPCVLMSPAYFLGRPLRWLEAISEYGGTISGGPDFAYRLCCERVSESALE
RLDLSGWRVAYSGSEPIRLDTLERFAEKFAACGFTQDSFMASYGLAEATLFVAGSPRGQGIGNLRVDDQA
LAQNRAEPGEGSAIMSCGISQPDHAVLIVDPTALNELADNTVGEVWAAGPSIAHGYWRNPEASAKTFVQH
AGRTWLRTGDLGFMRGGELFITGRLKDMLIVRGHNLYPQDIEQTIEREVEVVRKGRVAAFAVNIEGQEGI
GIAAEISRSVQKILPPDALIKAIRQAVAESYQETPSVVVLLNPGALPKTSSGKLQRSACRNRLADGSLDS
YALFPSADIESKSATESGSELQTLIGQIWCEQLSVKQISVDDHFFLLGGNSIAATQVVARLREALGLELN
LRLLFEAPTLGAFAVAVAQQQQDGGLAQGAITPLSRTEAMPQSLAQNRLWITWQLDPQSSAYNIPGALRL
RGELDEDALRASFQQLIERHESLRTRFFERDGVALQQVDAASEFNLQLIDISDLPVAEREVRAQQIREDE
ARTQFDLEKGPLLWVTLVRLDEEDHQLLVTLHHIIADGWSLNVLIDEFSRLYAAASQGQKATLPALPTQY
ADYGSWQRQWLAQGEGERQLTYWKAQLGDEHPTLSLATDHPRSAHHLHSAARHTVRLGATLSDAIRQTAQ
AYESTSFMLLLAAFQSLLHRYSGQRDIRIGVPNANRPRLETQGLIGFFINTQVLRAELDSQLPFVELLAQ
TRQAALGAQAHQDLPFEQLLEAFPQAREQGLFQVMFNHQQRDLSALRRLPGLLAEELPWHSREAKFDLQL
HSEEDRNGRLSLSFDYSSELFDAATIERLAEHFSNLLRAVCEQPKTAIGDLQLLTPPELDQQASWSAEPC
TPASVWLPELLNQQARLTPERTALMWDGGAMDFAELHTQSNRLAHYLRDKGVGPDVCVAIAAERSPQLLI
GLLAIIKAGGAYVPLDPDYPAERLAYMLSDSGVELLLTQTQLLERLPATDGVSMIAMDALHLESWPSHAP
GLHLHGDNLAYVIYTSGSTGQPKGVGNTHAALAERLQWMQNTYRLNDTDVLMQKAPISFDVSVWECFWPL
ITGCRLLLAGPGEHRDPHRIAQLVQQFGVTTLHFVPPLLSLFIDEPLTAECTSLRRVFSGGEALPAELRN
RVLEQLPAVQLHNRYGPTETAINVTHWHCTTADGERSPIGRPLGNVLCRVLDEALNPVPAGVPGELCISG
IGLARGYLGRPGLTAERFVVDPLGEQGARLYRTGDRARWTADGVIEYLGRLDQQVKLRGFRVEPEEIEAR
LLAQDGVAQAVVLVRETAAGGQLIGYYTATDSTEDVDAQTARLNAALAAELPEYMVPAQLMRLDEMPLSP
SGKLDRRALPEPQWQVREHVEPVTALEQQIAGIWREVLGQAQIGLRDDFFALGGHSLLATQIISRTRQAC
DVELPLRALFENSELGAFAEQVQLIQASGKTNQQPPIEKVDRSQPVPLSYSQQRMWFLWQMEPDSPAYNV
GGMARLRGVLDVGRFEAALHALIMRHETLRTTFPSIDGVARQRVHPDTGMRMDWKDFSALDADNREQRVQ
```

QLADDEAHKPFDLETGPLLRACMVKTAEHEHYLVLTLHHIVTEGWAMDIFARELSALYEAFVDDRESPLQ
PLPVQYLDYSVWQRQWLESGERQRQLDYWTAQLGREHPLLELPGDRPRPPVQSHQGELFRFDLSDTLAAR
VRAFNAQNGLTLFMTMTAALAVLLYRYSGQTDLRIGAPVANRIRPESEGLIGAFLNTQVLRCQLDGQMSV
GELFEQVRHTVIEGQSHQDLPFDHLVEALQLPRSAAYNPLFQVMCNVQRWEFQQSRTLAGMTVEYLANDA
RATKFDLNLEVTDLDHRLGCCLTYSTDLFDEPRIARMAGHWRNLLEALLADPQQRLSELPLLEATEQQNL
LDSLGVEPGEHRLDQCIHHLFSEQALARKDAPALTFAGQTLSYAELDSRANRLAWMLRERGVGPQVRVGL
ALERSLDMVVGLLAILKAGGAYVPLDPEYPLDRLHYMIEDSGIGLLLSDAAMFKALGELPSTVARWCLEE
DAAELANYPATELPFISLPQHQAYLIYTSGSTGKPKGVVVSHGEIAMHCQAVIERFGMRPDDCELHFYSI
NFDAATERLLVPLLSGAQVVLRAQGQWDAEEICGLIRRHNINILGFTPSYGSQLAQWLATQGEILPVRMC
ITGGEALTGEHLARIRAAFKPSLFFNAYGPTETVVMPLASLAPEQLEEGLGSVPIGSVIGARVAYILDAD
LALVPQGATGELYVGGAGLAQGYHQRAGMTAERFVADPFAATGGRLYRTGDLVRQRADGLVEYLGRIDHQ
VKIRGFRIELGEIETRLLEHDSVREAVVLALDAPSGKQLVGYLVTEVTEQDEAQQHTLREALKAHLKSQL
PDYMVPTHLILLASMPLTANGKLDRRALPAPDPELNRQQYVAPSNALELTLAAIWCEVLNVLQVGLNDNF
FELGGDSILSIQVVSRARQQGIHFSPRDLFQHQTVQTLAAVATRTEQVTAEQGLLTGESRLTPIQHWFFD
TDIPQRQHWNQALLLEPTVALEPHRLEQALLAVIEQHDALRLRFSEIAGQWQAEHQTVSDAPVLWQVRVS
SMDKCAALFADAQRSLDLEDGPLMRALLVDGPEGQQRLFIAIHHLVVDGVSWRVLLDDLQTVYRQLDAGQ
LAKLPAKTSAFKDWAARLQAYAGSESLREELGWWQSQLAGESAELPCDRPEGGRQNRHAQTVSVRLDVER
TRQLLQQAPSAYRTQVNDLLLTALARVLCRWSGHESALIQLEGHGRETLFDEIDLTRTVGWFTSAYPLRL
MPADEQGASIKAIKEQLRAVPHKGLGYGVLRYLADDSSRQTMAALPGAPITFNYLGQFDQSFGADALFRP
LDEAVGAAHDPRAPLPNELSVDSQVYGGELVLRWTFSAERHDLQTITDLADAYLGELQSLIEHCLHDDAG
GLTPSDFPLAKLTQPQLDALPIPAAAIEDVYPLTPMQEGMLLHTLLEPGTGLYYMQDRYRINSELDPQRF
DQAWQAVIARHEALRASFCWNVGEDMLQVIHKPGSTPIEYLDWTAIAETEQEPKLQALLKSEREAGFDLL
NQAPFHLRLIKVGAARYWFMMSNHHILIDAWCRSLLMSDFFEIYTALDEGREVQLAVPPRYRDYIGWLQR
QSLAEARQWWKTNLQGFERTTPIPSDRPFLHEHAGESGGMIVGDRYTRLDARDGVQLRELAQAHQLTINT
FAQAAWALVLRRLSGDRDVLFGVTVAGRPVDMPEMQRTVGLFINSIALRVKMPEDHQRCSVRQWLSGLLD
SNMQLREYEYLPLVSIQENSELPKGQPLFDSLFVFENAPVEVSVLDRAQSLNATSDSGRTHTNFPLTAVC
YPGDDLGLHLSYDQRYFDESTVERMLGEFKRLLLALVEGFHGDMADLPLLGAEEQNFLIHGCNQSEHDYP
LEQSYVSLFEAQVAAHPQRIAASCLGQQHSYLELNERSNRLGHALIAAGVRLDQPVALLAERNLDLLGMI
IGSFKAGAGYLPLDPGLPSQRLSRIIDLSRTPLLVCSQACREQALALLDEFGCANRPKLLVWEEVQASEI
SVANPGIYSGPNNLAYVIYTSGSTGLPKGVMVEQRGMLNNQLSKVPYLNLSDADVIAQTASQSFDISVWQ
FLAAPLFGARVDIVPNTIAHDPQGLLAHVAAQGITVLESVPSLIQGMLAQERMSLDGLRWMLPTGEAMPP
ELAHQWLLRYPEIGLVNAYGPAECSDDVAFFRVDMASTRGSYLPIGTPTDNNRLYLLDGALELVPLGAVG
ELCVAGTGVGRGYISDPLRTAQVFVPNPFGKPGDRLYRTGDLARRRSDGVVEYVGRIDHQVKIRGYRIEL
GEIEARLHEQPEVREAAVGVQDGVNGKHLVGYLVAADALINPTERLERIKQRLRAELPEYMVPLHWLWLD
RMPLNANGKLDRKALPVLEIGQLQSQDYQAPRNELEQTLADIWAEVLKVDKVGIRDNFFELGGHSLLATQ
IASRVQKALQRDVPLRAMFECSTVAELAEYIDGLAASDITEEKVDRLNDLMAELEGL
>Siderophore_-_pyoverdine~pvdS~KAA0974501
MTEQVSTSRCDSPLLQAFVDNRLILVKIAARITGCRSRAEDVVQDAFFRLQSAPQITSSFKAQLSYLFQI
VRNLAIDHYRKQALEQKYSGPEEEGLNVVIQGASPETSHINFSTLEHIADALTELPSRTRYAFEMYRLHG
VPQKDIAKELGVSPTLVNFMIRDALVHCRKVSGSRVDTFARR
>Siderophore_-_pyoverdine~pvdY~KAA0974502
MPNLNHLTALALPTGNSLVADETENRLSLSLEGQPLIQLRLTREPELHVQVEERFGRPDGQAFWAACYWL
LASDPARQHLTWHLDEAPTAALLSGLLISTEVVGQYRCERTLFWQLPQPWLGTSLTSNYPQQMVLSGGKR
HPLRPMKPRGEVYRRFDARLGAWISLRTVDIEHDLARFNRWQNSPRVASFWQEEGSLEQHREYLGKLEAD
PHTLTLIGCFDDQPFAYFEAYWAKEDRIAPFYDAGDYDRGIHMLVGEENHRGPHKVASWLSALVHYLFLD
DPRTQRVVAEPRADNAKMIGHMQNQGFYCEKAFDFPHKRAALMALGRERFFDRCLLA
>Siderophore_-_pyoverdine~pvdQ~KAA0961748
MIISRQLTRLSLAGVLVGLSLAANARSQPEQASADIRRTSFGVPHIRADNERGLGYGIGYAYAQDNLCLL
ANEITTVNGERSRYFGPDQFTVEERENLVSDLFFNWLNTPQAVEGFWQTQTPAVRDLIEGYVAGYNRSLT
EHRAQGLPPQCQGDWVREITIQDLVKLTRRLLVEGGVGQFAEALAGATPPKSVTHVAGDPVSFQVAASRM
QRFALDRGSNAVAVGSERSFNGRGMLLANPHFPWVGGMRFYQMHLTIPGKLDVMGAALPGLPMINIGFNQ
HLAWTHTVDSSKHFTLYRLQLDPKDSTRYLLDGKSLPMKKQTLTVDVKQPDGQTRPVSHVVYSSQFGPIV
QWPGKLDWDNQFAYSLRDANLDNDRVLQQWYAMNRAVSLKELQASVHKIQGIPWVNTLATDDQGQTLYMN
LSVVPNVSADKLARCSDPRAGLQMIVLDGSNSACAWDIDPQAAQKGIYAADKLPQLLRKDFVQHSNDSAW
MANPAQPLTGFSPLISQDGQPLGLRSRFALDRLNTLNKAGPIAAADLQHMVMDDQVYQAAQVMPDLLQFC
AAALGSDAPALTPLCASLKVWDRSANLDSGLGFVHFQNVMVSLQQTPDLWRVAFDPKDPQHTPRGLAVDR
PEVAKSVREAMLASVAQINKMGLKADSRWGDIQVVSSGGQQTPIHGGPGTLGVYNAIQSVPRTDGKLEVV
SGTSYLQIVTFDDKGPQARGLLAFSLSSDPASQYSRDQTLAFSRKQLSVLPFTEQQITSDPQYQVQTIRE
QDEKAGKVAAQ

>Siderophore_-_pyoverdine~pvdE~KAA0959584
MTQPTRGAIGELFALLKPFRLIVAASIFLGMVGGLSVTVLLATINNALHSDTGLTQGVVALFAGLCLLAL
ASSICSDIGTNYVGQHIIAKLRKQLGEKVLSAPIEQIERYRSHRLIPVLTHDVDTISDFAFAFAPLAISV
TVTLGCMGYLAILSWPMFLIMVAAILIGTAIQAYAQSKGVQGFMAAREAEDELQKHYNAIAEGAKELRIH
RPRRQRMFVSGIKATAEFICNTQVRSINTFVIAKTFGSMLFFVVIGLALALQTFWPSADKTVMSGFVLVL
LYMKGPLEHLISTLPIVSRAQIAFRRIAELSEQFSTPEPHLLLSDKGNVKQAVHELKLADVRFSFPAAEG
AAPFQLGPVNLTIKQGDITFIVGENGCGKTTLIKLLLGLYTPQQGEIRVNGEAIDAQNRDDYRQLFTTIF
ADYYLFDDVVQGDTHIPEDANKYLQRLEIAHKVSVKDGNFTTTDLSTGQRKRLALVNAWLEERPVLVFDE
WAADQDPTFRRIFYTELLPDLKRLGKTIIVISHDDRYFDVADQLVRMENGRVVTELAPA
>Siderophore_-_pyoverdine~pvdO~KAA0959585
MNRDQLTLPLKALALSALMAGLLPTAAQAAAPAQPGKVFKDCRDCPEMVVLPAGTFTMGTPDDEVGREPD
EGPMHEVTFAKPFAMSRFQVTAGEWDSYVRESGVTIANGDTRPGRECVASKPRYPQSPRQPAVCMNFDDV
KNYVAWLSKKTGQTYRMVSEAQREYAARGGSKGPFPFPFDEGKEYSIAKHANTYGPADGYSFSSPVGSYP
PNAFGMYDMHGNVYEWVEDCEHPDYVGAPTDGSAWVEPKCEAVRIRGNDWGEAPVFSRSGNRNSQYPQER
GDWMGFRIVRDL
>Siderophore_-_pyoverdine~pvdN~KAA0959586
MTNRRSFLKQAGILAAGLPLGAAVNLPALAATPAPLPKDKWAQLRQMFNQDPDYLHFANFLVTSHPKPVR
DAIDLHRANLDRNPGRAMDWDLGETEKREQEVRVWAGRYLKAKPEQIALTGSTTEGLSLIYGGIHVRPDQ
EILTSEHEHYATNYALGYRQQKDGVKVRKLKLFENSQNISVDEVLGSIAKGIRPQTRVLGMTWVQSGSGV
KLPIGEIGKLVDEQNRNREEKDRILYVVDGVHGFGVEDLDFPDMHCDFFIAGTHKWMFGPRGTGIICARS
TELKDVTPTIPTFSEATTFSTIMTPGGYHSFEHRWALNEAFKLHLDLGKAEVQARIHALNTYAKNRLLEQ
PQIELVTPMSPDLSAGFTFFRIKDRDCEKVAAQLMENRVVCDSVDRDVGPVIRIAPGLLNTENDIDRFMA
LLNKTT
>Siderophore_-_pyoverdine~pvdM~KAA0959587
MTKPRSKKALYIGLPLALAISAGAGFAAWDYWFKDNPGYPVKVMKQADELHERILSFDSHVTVPLDFGTA
GNEADKDGTGQFDLVKTGRGRLSGAALTIFGWPEIWNGANAPHRPTAGFVDEARFEQETRYKIISTMVRD
FPNQVAIAYTPDDFRRLHGEGKFAIFMSMLNAYPLGNDLNALDKWAARGMRMFGFSYVGNNAWADSSRPL
PFFNDSRDALGGLSEIGKQAVHRLNDLGVIIDVSQMSTKALEQVAQLSRAPMVASHSAPRGLVDIPRNLS
DQEMQLIKNSGGVVQIVGFPTYIRPLSQATQDKLNALRARFDLQPLQGLEMALMPGDPVITVWSEQRFGE
YASQLYAIVDEEPKATLKDYGDAIDYAVKKIGIDHVGISSDFNDGGGLDGWKDVSEARNVTAELISRGYG
EADIAKLWGGNFLRVWDQVQKSSKPVAKN
>Siderophore_-_pyoverdine~pvdP~KAA0959588
MTISRRWFMAGLALTGAAVPAAFYGHREWTKPDPTITPGEASFDVADVAGQHLANTLRGVWQIRFEGHDA
GIEGLPRDGLEVFLDIGHKGRGLSGFLDTAERLRSSEAPRYRVLGDLAVAKPAQLSWRLLSASGTADYEF
SLLLDEVWAGFGNAGSGTLSGRVLNLDRPLMLTAQDNRFIAVKRVFPEARERSGLNPTLLAWLVSPEHRL
FHQLWHASRDKWHTLSEDKRGALRGIGWQPGPRDKERDARGARKDRNGSGVDFFFMHRHMLGKARSLQDL
PSWSHFPLPQPELARDRVGFARYFDNHDGTALPPTWLADDDAEYSQWVSDIKTAETYHSNFQVWESQYRD
PRYLSKLTLGQFGSEVELGLHDWLHMRWASVPRDPSNGQPVPFARDPADFSARWYAPENDFLGDPFSSHV
NPVFWHFHGWIDDRLEDWFRAHERFHPGAVSRLEVNGVPWFAPGRWVEIDDPWLGPSTHGCATTPGLQAG
KSVEMDPETMKLALRITFGDDEKKLADLFRKVPQRPWYARHLKAKPITS
>Siderophore_-_pyoverdine~pvdA~KAA0959593
MTQAIASPIVHDLIGVGFGPSNLALAIALQERGPSQGELDVVFLDKQADYRWHGNTLVTQSELQISFLKD
LVTLRNPTSPYSFVNYLKHHGRLVDFINLGTFYPCRMEYNDYLRWVAGQFEAQSRYGEEVLTIEPVLHNQ
QVEALRVISRDTQGQQHVRTARSVVVSAGGTPRIPEAFKALKGDSRVFHHSQYLAQMAKQPCVNNQPMSI
AIIGGGQSAAEAFIDLNDSFPSVQVDMILRGSALKPADDSPFVNEVFSPEFTDLVFQQASSERERLVNEY
HNTNYSVVDIDLIERIYGIFYRQKVSGIARHAFRTLTTIEKATATERGIELAVRNNATGEVTVRHYDAVV
LATGYERQMHRKLLAPLEQYLGDFEVDRNYKLITDERCKAGIYMQGFCQASHGLSDTLLSILPIRADEIA
GSLYDHGKARGHGRSVMDLLLATAS
>Siderophore_-_achromobactin~acsA~KAA0975552
MNFTTMAADRVMQDLVDCLLAEHFFGTEPLNLLASAADQPFTGLSAEQRIWEWHNEPQGSILVALRPGIT
QQWEKVPGTPVLVRQDQQLTELSPEVFMTQVLAGMTDRYQDNEKGFALFLDVLRTSVRQTELSLAHRVNS
ERLLEKSNADFFLTMEQWASLRDRPYHPLAKAKQGLNDAEYQHYQAEFARPVALNWVAIDRTLLQCGDGV
TDLAQQRPADYLLPFALHAGLQHEMQQRGIYESHIALPVHPWQFDHVLQAQLGDAFAKGECQRLDFTDGD
FYATSSLRSMTPCFNSADYLKLPMAIYSLGASRYLPAVKMINGGLSEKLLRQGLDKDETLQQKLHLCDET
KWWAFMPPDATLFDEAPRHLAAMVRGYPPALLEDKDTRLVPMAALGTPLPGSNQHFFDDWMAYRQLPANA
ASVMTLFRELAHSFFDINLRMFRIGMLGEIHGQNAVLVWKAGHAEGLLLRDHDSLRIFVPWLERNGLADP
AYRLKKGHANTLYHERAEDLLFWLQTLGIQVNFRSIIETLALVYALPERGLWTAMGEVLNELIDTIDFDT

```
EARAMIKQQLFEAPHWPQKLLLTPMIERAGGPGSMPFGKGQVVNPFHRLHRED
>Siderophore_-_achromobactin~acsB~KAA0975553
MLRTNLLKQKLTSGAPAYGLISSIPSPASIELIAEAGFDFVIIDMEHVLINPETVEHMIRTAESYAITPL
VRVADLNPKTMLRLLDGGAQGIVLPMIEGPDALAESIRACKYHPQGGRSLNAGRPGAFGKHSLAEYIGLA
NEQIMVVAMIESAEGVRRAAEIAAVPGLDMILEGAADLSQSMGLPWQIDRPEVQAALLSSWQAANAAGVP
YCAIPRQPGDHERWLARGVKSFVLGDERGIAFRALQAKLAATSAEGK
>Siderophore_-_achromobactin~acsC~KAA0975554
MTMATSSQATPQAQDRAPGVWLASADTQRYQQVEQRVVGQLLQTLLYENVLPYRHSPLAGSQYHFVVSGL
DARQQPVEYHCTGLLSASFELIRLDYPSLERVDADGKHSQPHLHQALAELLGELQDSPHLPRFTHELEQT
LLKDLQSRSQGYQANVPSHQLDVDALEQHFMDAHSYHPCYKSRIGFSLKDNASYGPEFAAPVAIVWLAVA
KTCGAMNISRHLSYEDFIQQEYGLQRWQELAESLTDQGKSMGDYWLMPVHPWQWENVIVPVFYPELVSGE
VIHLGTTNDRYQAQQSIRTLANVTDKQRPYVKLALSMTNTSSTRILARHTVMNGPIITDWLHQLIRSDST
ARDLGFVILGEVVGVSFDYDHLPTTRMSQAYGTMGTLWRESIHQYLEDDEQAVPFNGLSSVDNRYGDGEQ
TPFIDAWIKQYGLHAWTRQLLQVAVSPIIHMLYAEGIGMESHGQNIVLIVKQGWPQRIALKDFHDGVRYS
PQHLGRPELAPTLVPLPASHAKLNRNSFILTDDVDAVRDFSCDSFFFIALAEMAIFLHQHYQLAETEFWQ
MTAEVITGYQVEHPQHRARYAAFDVFAPFYEVEELTKRRLLGDGERRFKSVPNPLHAFRLQSC
>Siderophore_-_achromobactin~acsE~KAA0975555
MDKLPATVLAAIDEARIQNEDPLALFIYDLDALKQHVTQVMAALPKGVELYYAIKANSEPQILATVAPLV
HGFEISSGGEIDRLQTCPTPKPFIFSGPGKLDSDLRSALQHQVEAIHIESLNEIVRLQRLALEAGRVQDV
LLRINPELPSPLSSKLAMAGTATPFGIDESELAQAVSWVDNASHLRLKGFHVHAMSHQSLVERHEQLLDL
YLERWPQWKALSANPATITHLNVGGGIGVNYLGPQQFDWQRLCAHLGESLAACADAPIVRFEPGRFISAY
CGYYAIEVLDSKTSHGKHFLVCRGGTHHFRLPVAQGHDHPVIHLPRHPATSGAVQQKWTVVGQLCTPKDV
LSRDCPLTGVEVGDMLVLPLAGAYGYNISHADFLCHPRPPQLFVQDAGGASWV
>Siderophore_-_achromobactin~acsD~KAA0975556
MNHPNRNVLPNMVSELATTCALLNCLIKEFALPENCLSYSWPTQMQGIAPGSYLEGLEWKGIPLTIKLPN
QQQFFVMVDRRDGLGSHRYLSDVYARRGEGDWSCLRFAEFVEQLLAACEHMTRASNDELLDQVLQSQLLT
AAIVGHNTPKDSDPLSGYLASEQGLWFGHPNHPAPKARLWPAHLAQETYAPEFQARTALHLFEVPLEGLS
VGANGLSKAQVLAGFADQAQARPGHAVICMHPVQAQLFMQDGRVQRLLKSKDIVDLGATGFMANPTASIR
TWYVEGHEFFIKGSLNVRITNCVRKNAWYELESALMIDRIFRNLQLTQPETLGGLSVVAEPGLLSWAPHS
ASEADSHWFREQTGAILRENFCLDSGTDCSIMAGTLFARGLHLRPLVHEFLTRFNGNDIDDQQLLSWFDD
YQALLLRPVLALFFNHGIVMEPHLQNSVLVHDNGRPQRLLLRDFEGVKLTDELGASRIDADVHPRVRESL
LYSRQQGWNRIVYCLFVNNLSEAVLALSWERPHLAPLMWQRVEQQLISIRAELTRAAPELDALIAGQPIA
CKTNLKVRLAAKADRQAGYVTLESPWGKESQHG
>Siderophore_-_achromobactin~acsF~KAA0975731
MLNDGVLHSNTLQKTNADYLARQSKFESNVRSYPRKLPLAIAKAHGLWVTDVEGNTYLDCLAGAGTLALG
HNHPAVMASLDSFLASGLPMHTLDLTTAVKDAFSETLLSLLPDQGRDYCLQFCGPSGADAVEAALKLAKT
YTGRNNIISFSGAYHGMTHGALALTGNTAPKNAIASLMPGVQFMPYPHEYRCPLGIGGEAGVEALTHYFT
QFIEDVESGVSLPAAVILEAVQGEGGVNCAPASWLRAIREVTRKHGILLILDEVQTGFGRTGKMFAFEHA
DIEPDLIVMSKAVGGGLPMAVLGIRREFDAWEPGNHAGTFRGNQMAMAAGLATLQVLQQQNLAVQAERRG
QWLKDRLVELQAHYPALGQVRGRGLMLGIEIVDERQPADRHGHFPTDPALAVAIQQQCFKQGLLLERGGR
KGNVIRLLPPLIIDDEQCQQVIQRFDNAMAAALLQLRP
>Siderophore_-_vibrioferrin~pvsA~KAA0939502
MTYSTVLITHVVNDAVIEGFIPALLKSRHVPDSQLIIITDDKEAHMDALIATPLGAQISILECDVFNPLA
IIDTLNNYHILPNLVFSNSDHLQAATAVVAEFFHLPSKDWRHCLVFKDKYLMRTQLHSAHLYQAGYQLLT
AKDLHLENLSISFPLVAKPKQGVASMDVQLCMNKNELLSYCQSFWQSKTLPILVESYVAGRLITLETLGD
GERLVAVGGFEVTLTEPPHFVELDAVWNTPLCVQYRERCLAILQDIGVGLGVCHSEFIITPTGEVHLVEI
NYRSIGDGREFLLHDMAHYPWFDTIVRLHQGVSIAELEAEFGQLAISTHARIHYIVAPQSGTICIAPSPF
NKQMQQTTIIHQPLKKQHQTIHITHSNKDYLSRLLAYSDCANTMNDVLNDAIANLQWQFES
>Siderophore_-_vibrioferrin~pvsB~KAA0939503
MMDKQYITKRFINTCIRENIYGLLSNNSIIETEFDIPDLPEVLKQQKLWLKITSSNQTNWLPIVASDYMQ
YWQLASPMWLQKNSDGYLIKHDYQDFVAILKQLPDACETALDSYLLELDCATEHRALARQGFESQRCHLK
RNINGYPSWSERLLYADQVASYLDHPYYPTARAKLGLDASDIAAYSPEFAPTFSLYWVAISKAISTVSAK
LPDVWPSFTDVGLSPDLQQTHHLFPIHPLTLPLLHQHVLKDTSLQIILAPKSALSVQPTLSVRTVVCCAD
PNIHIKVPLIVRTLGNKNVRLIKPSTIYDGHWFQRTLQFIEKNDAALQECYAHCDESLGGHMGDDKTFAF
IVRHYDDINLTDKTPVPVAAFGSVMPDGRPFISHLIAQFYDGDSKLWLKEYIHLLCHVHLTLWIKYGIAL
EANQQNAVIVFYHHSRTMTLLMKDNDSARLYPPRFNASMAQVGLGDVIKSSAINDIKDIKDQRIVVEDEL
ALAQMFLTITLQLDIVAIIEMMSAYNIINRHDAYQTLIDCIDTTLHSLSKQGYDIHLAKQVLLESQHWYI
KYLLSAGSLLSKEQSGASDINKFYGKSAPNTLKLMQQHKQDS
>Siderophore_-_vibrioferrin~pvsC~KAA0939504
```

```
MSYRIMVAIVLCCHYLSAFSALGMPLFLPIMLQELNAPLQSGWVGLLFILPTLMTAIAARYWGRFADTYG
RKTSLLRAELGLTVGFLICGFSQSLWQLVIGLSVQGLFGGTMAASNAYLATSITDKNALKQVLNFTQFSA
RLALITAPIGLGFLVHHYEVLSLYRVLAILPLLALLLSFSLKKDVIAAGNIGTTANTTELPKASTRTKTN
NINSNTAKSDKKKTQHSVQAITGTAFFANPYLFISVLQFFLAFCMVATFPYFLPFAKIFGLSTSQIGQLY
AMPHVMYLICQLLYGRISQRLPAMSLSLGFTVLASAVLLHLTDYLLLIWLARMLFGVGMFMCYQSLHAQM
ADTNQSKHSGKIFSRYDALAKWGGVLAGLSVTLFVPIFGLLSPFVLSAMMAVIAIITIKIYQEKYSSIV
>Siderophore_-_vibrioferrin~pvsD~KAA0939505
MRPNYSLSNNTIPNFLPPSKTVENTPVPSLLSQYHQYKSEQHALTNIINCYLREFAIPNQDLSIDESDDD
LPLCLRDNRVCLITRLLTIRFPVANEAASAKIVLPVAYFSHLGKCQPSGAPWLKARGQSWACLNIHQLVD
YLHSYIATRYQIDINQELKYQIDNSIRVTTAFLQTSANHATDYHHDLQQDKSTVNGYIDSEQALLWGHAY
HPSPKSRQGVDMTSLLAHSPETQSQFALFWFKVDSSLVSYIGKDTKTILRNIAHTLLADADDLPESINSD
TDSLHPDSDTDWVYYPCHPWEADDILANPTIKRAVTSGYMIPLGLTGKAVSPTSSVRTLYHHQLSHFIKC
SIHVRLTNCIRKNAWYELHSAVYLNAILEKIDHSKRLTAPAFKLMLEPGAATLDMSAKFPDDDPSSTQFI
TESFGMLFRESFTTDEIKYLKPQVAAALFTEDRFGDSVIERQLQKHCVHTGQRDNEHSSYDTLACLWFSR
YANTLIDGTFDYFFNHGVVFEPHLQNTVIGFHQGLPTCVWIRDLEGTKLVDSLWSLAEATGLSERCKQSI
RYSKAAGWSRIAYCVFVNNLSEAIFYLCRGAQSAEILEARLWQHVKEILMNWQLVYGIQPEIQDLLAGGS
LPSKTNYKTRLLTLADKHSAYVMLPCPWQSSMLNANKDNIKTHKSSESKVA
>Siderophore_-_vibrioferrin~pvsE~KAA0939506
MMTNATTTHLIETSDKHSLPQKIIDCIHNYGAQPSPLCAYIYDLEALEERITTMKRCLPDNTQLFYAIKA
NAEPKILSTLAEHVDGFEAASAGELNWLQTHQPNHPIIFGGPGKLTSDLQQAIHQQIDTIHVESLVELTR
IQILSQALQLPVRIFLRMNIDIGDIGSSKLVMGGKPTPFGLDETHLKQALAIIDSSPFIEFCGFHFHLMS
HQLSVERHLLLMQRYMEMVKKWQHDYPSLPSNLTINVGGGIGIHYDQQPQHFEWEVFCQQLASILTANLQ
FTVRFEIGRFITAPCGYYVMEVLDIKKNLGETFVIGHGGTHHFRTPAAQQHNHPFFIVPRRMSHAMENKL
QANLIEQAAITGTKATLVGQLCTPKDILAKDQWIDALHIGDYLVFTLAGAYAWNISHQNFLMHQPPKMIF
L
>Siderophore_-_bacillibactin~dhbF~KAA0933946
MPDTKDLQYSLTGAQTGIWFAQQLDPDNPIYNTAEYIEINGPINIALFEEALRHVIKEAESLHVRFGENM
DGPWQMINPSPDVQLHVIDVSSEPDPEKTALNWMKADLAKPVDLGYDPLFNEALFIVGPDRFFWYQRIHH
IAIDGFGFSLIAQRVASTYTALIKGQTAKSRSFGSLQAILEEDTDYRGSEKYEKDRQFWLDRFADAPEVV
SLADRAPRTSNSFLRHTAYLPPSDVNALKEAARYFSGSWHEVMIAVSAVYVHRMTGSEDVVLGLPMMGRI
GSASLNVPAMVMNLLPLRLTVSSSMSFSELIQQISREIRSIRRHHKYRHEELRRDLKLIGENHRLFGPQI
NLMPFDYGLDFAGARGTTHNLSAGPVDDLSINVYDRTDGSGLRIDVDANPEVYSESDIKLHQQRILQLLQ
TASAGEDMLIGQMELLLPEEKEKVISKWNETAKSEKLVSLQDMFEKQAVLTPERIALMCDDIQVNYRKLN
EEANRLARLLIEKGIGPEQFVALALPRSPEMVASMLGVLKTGAAYLPLDPEFPADRISYMLEDAKPSCII
TTEEIAASLPDNLAVPELVLDQAVAQEIIKRYSPENPDVSVSLDHPAYIIYTSGSTGRPKGVIVTQKSLS
NFLLSMKEAFSLGEEDRLLAVTTVAFDISALELYLPLISGAQIVIARKETIREPHALAQMIENFDINIMQ
ATPTLWHALVTSEPEKLRGLRVLVGGEALPSGLLQALQDLHCSVTNLYGPTETTIWSAAAFLEEGLKGVP
PIGKPIWNTQVYVLDNGLQPVPPGVVGELYIAGTGLARGYFHRPDLTAERFVADPYGPPGTRMYRTGDQA
RWRTDGSLDYIGRADHQIKIRGFRIELGEIDAVLANHPHIEQAAVVVREDQPGDKRLAAYVVADAAIDTA
ELRRYMGASLPDYMVPSAFVEMDELPLTPNGKLDRKALPAPDFSTSVSDRAPRTPQEEILCDLFAEVLGL
ARVGIDDSFFELGGHSLLAARLMSRIREVMGAELGIAKLFDEPTVAGLAAHLDLAQSARPALQRAERPEK
IPLSFAQRRLWFLHCLEGPSPTYNIPVAVRLSGELDQGLLKAALYDLVRRHESLRTIFPESQGTSYQHIL
DADRACPELHVTEIAEKELSDRLAEAVRYSFDLAAEPAFRAELFVIGPDEYVLLLLVHHIVGDGWSLTPL
TRDLGTAYAARCHGRSPEWAPLAVQYADYALWQQELLGNEDDPNSLIAGQLAFWKETLKNLPDQLELPTD
YSRPAEPNHDGDTIHFRIEPEFHKRLQELARANRVSLFMVLQSGLAALLTRLGAGTDIPIGSPIAGRNDD
ALGDLVGLFINTLVLRTDTSGDPSFRELLDRVREVNLAAYDNQDLPFERLVEVLNPARSRATHPLFQIML
AFQNTPDAELHLPGMESSLRINSVGSAKFDLTLEISEDRLADGTPNGMEGLLEYSTDLFKRETAQALADR
LMRLLEAAESDPDEQIGNLDILAPEERSSMVADWQSVSEKIPHACLPEQFEKQAALRPDAIAVVYENQAL
NYAELNERANRLARMLISEGVGPEQFVALALPRSLEMAVGLLAVLKAGAAYLPLDPDYPADRIAFMLKDA
QPAFIMTNTKAANHIPPVENVPKIVLDDPELAEKLNTYPAENPKNKDRTQPLSPLNTAYVIYTSGSTGVP
KGVMIPHQNVTRLFAATEHWFRFSSDDIWTMFHSYAFDFSVWEIWGPLLHGGRLVIVPHHVSRSPEAFLR
LLVKEGVTVLNQTPSAFYQFMQAEREQPDLGQALSLRYVIFGGEALELSRLEDWYNRHPENRPQLINMYG
ITETTVHVSYIELDRSMAALRANSLIGCGIPDLGVYVLDERLQPVPPGVAGELYVSGAGLARGYLGRPGL
TSERFIADPFGPPGTRMYRTGDVARLRANGSLDYVGRADHQVKIRGFRIELGEIEAALVQHPQLEDAAVI
VREDQPGDKRLAAYVIPSEETFDTAELRKYAAERLPDYMVPAAFVTMKELPLTPNGKLDRKALPAPDFAA
AVTGRGPRTPQEEILCDLFMEVLHLPRVGIDDRFFDLGGHSLLAVQLMSRIREALGVELSIGNLFEAPTV
AGLAERLEMGSSQSALDVLLPLRTSGDKPPLFCVHPAGGLSWCYAGLMTNIGTDYPIYGLQARGIGQREE
```

LPKTLDDMAADYIKQIRTVQPKGPYHLLGWSLGGNVVQAMATQLQNQGEEVSLLVMLDAYPNHFLPIKEA
PDDEEALIALLALGGYDPDSLGDKPLDFEAAIEILRRDGSALASLDETVILNLKNTYVNSVGILGSYKPK
TFHGNVLFFRSTIIPEWFAPIEPDSWKPYISGQIEQIDIDCRHKDLCQPEPLAQIGKVLAVKLEELNK
>Siderophore_-_bacillibactin~dhbB~KAA0933947
MAIPAIQPYQMPTASDMPQNKVSWVPDPNRAVLLIHDMQNYFVDAFTAGASPVTELSANIRKLKNQCVQL
GIPVVYTAQPGSQNPDDRALLTDFWGPGLNSGPYEEKIITELAPEDDDLVLTKWRYSAFKRTNLLEMMRK
EGRDQLIITGIYAHIGCLVTACEAFMEDIKAFFVGDAVADFSLEKHQMALDYAAGRCAFTVMTDSLLDQL
QNAPADVQKTSANTGKKNVFTCENIRKQIAELLQETPEDITDQEDLLDRGLDSVRIMTLVEQWRREGAEV
TFVELAERPTVEEWQKLLTTRSQQVLPNADYL
>Siderophore_-_bacillibactin~dhbE~KAA0933948
MLKGFTPWPDELAETYRKNGCWAGETFGDLLRDRAAKYGDRIAITCGNTHWSYRELDTRADRLAAGFQKL
GIQQMDRVVVQLPNIKEFFEVIFALFRLGALPVFALPSHRSSEITYFCEFAEAAAYIIPDAYSGFDYRSL
ARQVQSKLPTLKNIIVAGEAEEFLPLEDLHAEPVKLPEVKSSDVAFLQLSGGSTGLSKLIPRTHDDYIYS
LKRSVEVCWLDHSTVYLAALPMAHNYPLSSPGVLGVLYAGGRVVLSPSPSPDDAFPLIEREKVTITALVP
PLAMVWMDAASSRRDDLSSLQVLQVGGAKFSAEAARRVKAVFGCTLQQVFGMAEGLVNYTRLDDPEEIIV
NTQGKPMSPYDEVRVWDDHDRDVKPGETGHLLTRGPYTIRGYYKAEEHNAASFTEDGFYRTGDIVRLTRD
GYIVVEGRAKDQINRGGEKVAAEEVENHLLAHPAVHDAAMVSMPDQFLGERSCVFIIPRDEAPKAAELKA
FLRERGLAAYKIPDRVEFVESFPQTGVGKVSKKALREAISEKLLAGLKK
>Siderophore_-_bacillibactin~dhbC~KAA0933949
MLDQNVITETKAEHLLHEYQPGAFFLASPHRVLLAKGICEIVPEADGQNQMETLSGRIAEALRQAKQSGQ
SRPLVVGAVPFDQVKAARLVVPEEVRWSGPLQFDHEEKEQQAGHTYHIKPIPEPEDYKNGVEQGLTRIAD
GTLSKIVLSRSLHLTSPEPIQTDELLRHLAQHNTHGYTFAADVSSQEETSPRRTLLGASPELLVSRMGTQ
VVSNPLAGSRPRSNDPVEDQRRAAELLSSAKDLHEHAVVADAVAAALRPFCRTLEVPEKPSLIKTETMWH
LSSVIKGELSDPSVTALELAAALHPTPAVCGTPTDLAREAILSIEPFDRGFFTGMVGWCDDAGDGEWIVT
IRCAEAEERSLRLYAGAGVVAGSKPEDELQETSAKFRTMLRAMGVDHI
>Siderophore_-_bacillibactin~dhbA~KAA0933950
MNAKSIEGKIAFITGAAQGIGEAVARTLASQGAHIAAVDYNPEKLEKVVSSLKAEGRHAEAFPADVRDSA
AIDEITARIEREMGPIDILVNVAGVLRPGLIHSLSDEEWEATFSVNSTGVFNASRSVSKYMMDRRSGSIV
TVGSNAAGIPRTSMAAYASSKAAAVMFTKCLGLELAEYNIRCNIVSPGSTETDMQWSLWADENGAEQVIK
GSLETFKTGIPLKKLAKPSDIADAVLFLVSGQAGHITMHNLCVDGGATLGV
>Surfactant_-_surfactin~fenE~KAA0937135
MNTIKKIKNIYPLSHMQEGMLFHSFLRKEEGAYVEQSLFTIKGSLSYDWFQRSIQAIIDRHDIFRTVFLP
HVPHLSGPRQVVMTEREFHLNSEDISHLPTNDQNEYIERFKEKDKQKGFDLQKDMLMRISLFKTAKDEHV
CIWSHHHILMDGWCLGIVMQEFMQIYQSIHAGKPLSLDPVRPYSTYISWLTNRDKEKAAAYWDTYLKNYS
APSPLPRVSDKETKEGYHREDLIFSLNKPLTDKLKETAKQHGVTLATLIQAVWGVMLQQYNRTDDVVFGA
VVSGRPSEIPGVEQMIGLFINTIPIRIKTHQDETFHELLIRCQKEMLEAEPFTCQPLFDIQANTALKQEL
IDHIIVFENYPLQQKIADSADQTDSPLQIDQVQVSEQSGYNFNLVVAPGEELVIKFSYNAFVYDAAWISC
IKRQFTQALSTAAQHPHMPIADFSFLDATEKEQIVTQFNNTKTEYPKNHTIIDLFREQAEKTPDHTALVY
GNMSISYKELDKRSNALARELIQKGFRQNETAGILAAHSPEFMISVLAVLKAGGAYLPLDAELPPERVSF
MLEETQAKMLIVQKGLEQNAAFSGTCMIADAQALMEENHFPINSSSSPDDLAYIMYTSGSTGRPKGVMIT
NRNVVSLVSNSNYTSASGDDRFIMTGSISFDAVTFEMFGALLNGASLHIIDKSTMLTPDRFGAYLLENDI
TVLFLTTALFNQLAQVRADMFRGLHTLYVGGEALSPALMNAVRHACPDLALHNIYGPTENTTFSTFFEIK
RDYAGPIPIGKPISNSTTYILDTKGRLLPIGVPGELCVGGDGVAKGYLNRDDLTNALFSPHPFLPGERIY
RTGDLARWLPDGNLEYISRIDRQMKIRGKRIEPAEIEARLLEMEGVQEAAVTLREKDGEAQLYTHYVGDH
KKTDTDFRADLARVLPDYMIPQHWVRVERMPLTGNGKIDRSALPIPENKPAKRQDIILPRNLVEEELANI
WKQVLGVNTISIDDDFFAIGGHSLKALQVIHTLKHQQNIDIPIDFLFEHPTIAQLAEKLYSKQLTAANEQ
HVIKLNQHGAQNLFCFPPISGFGIYFKDLALLLNEKAALYGFHFIEQDTRIEQYVNCMTDIQPEGPYVLL
GYSAGGNLAFEVAQAMERKGLEVSDFIIVDAYLKEQPLPIDTGNDESAAYLPEAVREKVMKKKRNYQEYW
AQLLNEGHIKASIHFIEAGIHPETSGHTGLTKWEGACGNYSEYTGFGAHKDMLEGTYAEKNADIILDILE
KITSNQVILHKR
>Surfactant_-_surfactin~fenD~KAA0937136
MTKANSIQDIYPLSYMQEGMLFHSLLQKDSQAYVEQASFTIEGKVNPQFFQNSINALVERHDIFRTIFIS
QNVSSPQQVVLRERNVIVLEENITHLNEAEQSQFIEQWKEKDRDQGFHLQKDVLMRIALIQTGESQYSCI
WTFHHIMMDGWCLSIVLKEFLHIYASYVNASPITLEPVQPYGKYIKWLMEQDKEQAVSYWDHYLSGHEQQ
TVLPKQKKTKGKSRQEHVTFSFSKEESSRLSELAAREEVTLSTIFHTIWGILLQKYNNNDDAVFGSVISG
RPAEIEGIEHMVGLFINTMPVRVQGAKTPFLQLIKDMQKDRLAAEAYSYHPLYEIQSRSAVKQGLIDHIL
VFENYPVQQEIQMLNKQEHASDLFQIHNFTVADETNYSFYLMVAPGEEIHIKMNYDAEQHDRSFVLSVKE
HLLNAVSQILNNPNLPPEEIDITTDTEKRQLIGEITDQTPVYETIHAMFEKQAEKTPDAHAVIDQACSLT
YRELNKAANRLARHLRMKGVVRQEPVAIMMERSAAFITGVLGILKAGGAIVPVDPHYPADRIRYILHDCG

CSHVVSQAHLPSSLEDNYIITHPEDIESKVDGSNIKSVNNADDLLYMIYTSGTTGKPKGVQFEHRNMANL
LKFEYTHSGIDFEADVLQFATPSFDVCYQEIFSALLKGGTLHIVPEAIKRDVPQLFAFINKHQTNIVFLP
TAFIKMIFSERELANSFPDGVKHLIAAGEQLMISDLFQDVLRKRGIHLHNHYGPSETHVVSTYTIHPGDP
IPELPPIGKPIGCTDLYILNHQKQLQPCGVPGELYISGASVARGYVNHDKLTSDKFSSDPFKPDVIMYRT
GDLARRLEDGNIEYIGRADNQVKIRGYRIEPQEIEVTLMNHPDISEAAILIWQDQNGEHELCAYYCSVQK
LNAIDLRSYMASELPEYMIPSKWIWVDSIPLTPNGKVDRAALPEPDASISGNPYTAPRNLLEAKLSQLFE
DVLKNGHIGIQDNFFDNGGHSLKATVLMSRIAKEFHVQVSLKDIFAHPTVEGLALIIREAEQNLYAAIEP
AEERDTYPVSSAQKRIYVLQQLDEGVAYNMPAVLELEGALDVAKLSAVCKELISRHEPLRTSFVSGADDE
PVQRIHTEVPFTLSKETTIEGFVRPFCLSQAPLFRAGLIEVSNEKHVLLVDMHHIISDGVSVQLLIREFT
DLYANRQLKPLRIQYKDYAVWQQKFKKGDSYQKQETYWQQQFSGDLPILELPTDKRRPAERQFIGGKVTF
QLDKEITARIKRLAHKNRSTLYMTLLALYSAFLSRLSGQDDIVIGSPIAGRPHADLEAVLGMFVNTLALR
TRPAGNKTFEEFLKEVRQTALEAYEHQDYPFEELVDKLGVQREMSRNPLFDTTLVLQNMEQQKLKMNDVQ
LQWNDLEHPISKFDISLYVTEHDSELFCQFEYSTALFEKETIQRWASLFTTLVEHTAASPETELDNIPIL
TKEEERDFIESCHLFEETGYSMNQTLHYALEQQAEKTPDQAAVIFEDGVMTYKELNEQANRIAWELIGRG
VKPETTVAIIGKRSPEMLLGIYGILKAGGAYLPIDPDYPEERISFLLEDSGTNILLLQSAGLHVPAFTGE
IVYLNQTNSGLAHRLSNPNVDVLPQSLAYVIYTSGSTGMPKGVEIEHRSAVNFLNSLQSRYQLKHSDMIM
HKTSYSFDASIWELFWWPYAGASVYLLPQGGEKEPEVIAKAIEEQKITAMHFVPSMLHAFLEHIKYRSVP
IKTNRLKRVFSGGEQLGTHLVSRFYELLPNVSITNSYGPTEATVEAAFFDCPPQEKLERIPIGKPVHHVR
LYILNQNQRMLPVGCIGELYIAGAGVARGYLNRPALTEERFLEDPFYPGERMYKTGDVARWLPDGNIEFL
GRTDDQVKIRGYRIEPGEIEAALRSIEGVREAAVTVRTDSGEPELCAYIEGLQRNEVRAQLERLLPGYMV
PAYMIEMKQWPVTPSGKLDRNALPAPGGAADAETYTAPRNVTEMKLSQLWEDVLKNGPVGIHDNFFDRGG
HSLKATALVSRITKEFDVQVPLKDVFAHPTVEGLAAVIREGTDSPYEAIKPAEKQETYPVSSAQKRIYVL
QQLEDGGTGYNMPAVLELEGKLNPERMDRAFKELIKRHESLRTSFEQDAGGDPVQRIHDEVPFTLQTTVL
GERTEQEAAAAFIKPFDLSQAPLFRAQIVKVSDERHLLLVDMHHIISDGVSVNILIREFGELYNNRNLPA
LRIQYKDYAVWQEGFKTRDAYKTQGAYWLKQLEGELPVLDLPADHVRPPVRSFAGDKVSFTLDQEVASDL
HKLARENGSTLYMVLLAAYTAFLSRLSGQEDIIVGSPIAGRPHKDLEPILGMFVNTLALRTRPEGGKPFV
QYLQEVRETALEAFEHQDYPFEELVDKLELTRDMSRNPVFDAMLVVQNNDYERLHLHDLQMKPAQVSHLV
SKFDLTLQASEGDGNIHFLFEYSTALFEKTTIERWASHLTNVLSIIGKNPEVTLNHIDILTQEERHQLLN
EFNTRQANQYGVQTISQLFEQQAARTPKATALVSGDKTLTYQELDEWSNGIARALRNRGVKPDTPVGIMM
HRSFSMIASILGVWKAGGCYVPIDPEYPKERKRYILSDSGTKLLMTINEADLGVLADFEGEILTIESVEE
DDKSPLPQMSSAHHLAYIIYTSGTTGRPKGVMVEHKGIANTLQWRRNAYAFNETDTILQLFSFSFDGFIT
SMFTPLLSGAKAVLLHEEEAKDILAIKHQLSRQRITHMIIVPVLYRALLDVVQPEDVKTLRVVTLAGEAA
DRELIARSLAICPHTELANEYGPTENSVATTVMRHMEKQAYVSIGQPIDGTQVLILNSNHQLQPIGVAGE
LCIAGTGLARGYVNLPELTERAFTQNPFKPEARMYRTGDAARWMADGTLEYLGRIDDQVKIRGYRVETKE
IESVIRCIKGVKDAAVVAHVTASGQTELSAYVVTKPGLSTNAVRSELQNKLPVFMHPAFIEKLDSLPLSP
NGKLDRGALPKPVYNHEGERPFLPPSSKMEQILADIWKEVLGAEKIGTADSFFELGGDSIKALQVSARLH
RIGKQMAVKDLFSHPTIQELAAYIRDSDTSSSQAAVEGDVQWSPVQKWFLSQDIKEKHHFNQSVMLHRST
SVQEDALRKTLKAITCHHDALRMVFTQNEQGKWDQYNRPLSHSDDALYGLQMIDLSAPDGTDGNRAYEPI
IKRHVLDIQQKMDLKNGPLLQAGLFHTIDGDFLFLSAHHLVVDGISWRVLLEDLALGYRQAAGGGKEIQL
PPKTSSFKAYTKKLSDYAESQQLMKQLKYWREAEEYQTEALPFDHIDGTRAHEGQRSTISFTLNEKETAA
LLKDANSAYNTDTQDMLLASVILALRHWTNQSAFKLSLEGHGREDVLKGIDVSRTIGWFTAIYPLLIKLN
ADLPDSEESMVHVLKTTKDTLRRVPDKGFGYGVIKYLTPPGKKDINFTGAPEISFNYLGQFESGRTAEVP
EEDAFSFSPLGAGGDISTTWNREQSLDISAIAAEGKLTVNMTYDNARFQRKTIEQLSETCRQFLLQLIEH
CQNKSETEKTISDFDDQELTEDALQEIADMLSFH
>Surfactant_-_surfactin~fenC~KAA0937137
MPQQPEIQDIYPLSFMQEGMLFHSLYDEQSRAYFEQASFTINGQLDLERFQKSMDAVFDRYDIFRTAFIY
KNVAKPRQVVLKQRHCPVHVEDISHLNEKDKEHCTEAFKEQDKSRGFDLQTDVLMRISILKWAPDHYVCI
WSHHHILMDGWCLGIVIKDFLHIYQALGKGQFPDLPPVQPYGTYIKWLMQQDREEAAEYWKKRLQHFEKA
SPLPKRTDQMSDGTLQQITFTIPEKETSELQKIAAACGATLNTVFQALWGIMLQKFNRRDDAVFGSVISG
RPSELKDVENMVGLFINTIPIRVQSDFLSFSDLVSGMQKDMTEAEAYSYFPLYDIQAQSALKQELIDHII
VFENTPTQQEIEELNQAGSFDFSVKDFEMEEVTNYRCSVKVIPGRTLYVRIHFHTGAYQPNMMSEIKDYL
QHMISDVISDPSLPVSKMTLLDEDKTRKIVSQNNRTVSVSPKAPTLHGLFERQAAVTPERPAIRFSGGLL
TYAELDMYASRLAAHLAARGVTNESIVGVLSERSPDMLIAVLAVLKAGGAYLPLDPAYPKERLSYMLKDS
GASLLLTQPGCSAPNFSGETLEVDMTSLASEKAENHLFTPADGGSLAYVIYTSGSTGQPKGVAVEHRQAV
SFLTGMQYQFPLSEDDIVMVKTSFSFDASVWQLFWWALSGASAYLLPPGWEKDSALIVQAIHQEKVTTAH
FIPAMLNSFLDQAEIERLRDSTSLKRVFAGGEPLAPRTAARFASVLPQVSLIHGYGPTEATVDAAFYVLD
PERDRDRLRIPIGKPVPGARLYVLDPHLAVQPSGVAGELYIAGAGVARGYLNRPALTEERFPEDPFYPGE

RMYKTGDVARWLPDGNIEFLGRLDDQVKIRGYRIEPGEIEAALRSVEGVREAAVTVRTGSGEPELCAYIE
GLERNEVRAQLERLLPGYMVPAYMIEMEQWPVTPSGKLDRNALPAPGVAADAETYTAPRNVTEMKLSQLW
EDVLKNGPVGIHDNFFDRGGHSLKATALVSRIAKEFDVQVPLKDVFAHPTVEGLAAVIREGTDSPYEAIK
PAEKQETYPVSSAQKRIYVLQQLEDGGTGYNMPAVLELEGKLNPERMGRAFKELIKRHESLRTAFEQDAG
GDPVQRIHDEVPFTLQTTVLGARTEEEAAAAFIKPFDLSQAPLFRAQIVKVSDERHLLLVDMHHIISDGV
SVNILIREFGELYNNRKLPALRIQYKDYAVWQEGFKTGDAYKTQEVYWLKQLEGELPVLDLPADHARPPV
RSFAGDKVSFTLDQEVASGLHKLARENGSTLYMVLLAAYTAFLSRLSGQEDIIVGSPIAGRPHKDLEPIL
GMFVNTLALRTRPEGGKPFVQYLQEVRETALEAFEHQDYPFEELVDKLELTRDMSRNPLFDVMFVLQNMD
QESLELNELCLKPAAHNGHQTSKFDLTLYTQEQPRGLLTFQMEFSTDLYKKKTIEKWLQYFINMLLSIIK
DNKAALGTINILNEDETHYLIHELNRTKIDYPRNETISRLFEMQAEQTPNAVAIVSDTQVFTYEDLNSWA
NQIASVLQIKGVGPDSVVALLTGRTPELIAGMLGILKAGGAYLPIASDLPVERIAYMLSDSGAALLLQSE
KTEKQLLDIECEQIIIEDIQKQGKTKNVDSSAGPHSLAYIIYTSGSTGKPKGVMIEQRSVIRLVKNSNYI
TFTPEDRLLMTSSIGFDVGSFEIFGPLLNGAALHLSDQQTFLDSHQLKRYIEHQGITTIWLTSSLFNHLT
EQNEQTFSQLKHLIIGGEALSPSHVNRIRNVCPEVSIWNGYGPTENTTFSTCLHIQKTYELSIPIGRPVG
NSTAYILNQWGVLQPVGAVGELCVGGDGVARGYLGRPDLTKEKFVPHPFAPGDRLYRTGDLARWLPDGTI
EYVGRIDDQVKVRGYRVELGEIETAFRQIDGVKEAAVLARTAQTGSKELFGYISVKAGTNAEQVRSLLAC
SLPNYMIPAYIIEMENLPLTSNGKLNRKALPEPDFASKQTYIPPRNELEEQLALIWQEVLGIQRIGIEDS
FFELGGDSIKALQVSARLGRHGLSLQVSDLFRRPKIKDLSPFIRKTERVIEQGPVQGDVPWTPIQKWFLS
QDIEERHHFNQSVMLFHTDRLSEDALRASLKKLAEHHDALRMVYRNDDRRWIQINQGIHESQLYSLRTFD
LSQSESGWETQIKQEVADLQQSINLQEGPLLHAALFKTLTGDYLFLAIHHLVVDGVSWRILLEDLSAGYQ
QAAAGQTIQLPPKTDSYQEYARRIQEYAQSSKLIREEAYWRTVEEQQDAELPYEIPHQVNIDFSKRDSLN
FSLTEADTAVLLQNVNHAYGTDTQDILLTAASLAICEWTGGSKLRIAMEGHGREHILPELDISRTVGWFT
SMYPALISFENHRDELGTALKTVKDTLGRIPNKGVGYGMLKYLTHPENKSITFSKTPEISFNYLGQFNDI
ERQDTFRPSSLGSGKDITHTWKREQIIEMSAMAADKKLHFNLSYPPARFHRNTMEQLINRIEHFLLDIMK
HCAGQQKTEKTLSDFSSQSLTAEDLDSISSLVEEL
>Surfactant_-_surfactin~fenB~KAA0937138
MAQSAQIQDIYPLSHMQEGMLFHSLMDFSSKAYIEQTSFTITGNLCVDSFQKSLNLLVSRYDIFRTIFIK
EVPDLTGPQQVVLSNRELTVYREDISRLADQEQQTLIDAFMTKDREKGFDLQKDPLMRLALFDRGDSQYT
CVWTHHHIIMDGWCLGIILKEFFSMYDSLKNNSPVQLGSTVPYSRYIEWLGEQDQEETAAYWSEYLKEYG
NTASIPRIKRRTADGNYKADQVSFSLAQEMVEKLTKAAQNWGVTLNTLFMSIWGVLLHRYNAADDAVFGS
VISGRPSAIDGIESMVGLFINTVPVRIRSAEGITFSSLVKAVQEDILSSEQHGYYPLYEIQNHSPLKQGL
IDHIFVFENYPVQLHHALSVESENDEGALKLSDISMSEQTNYDFNIVIVPGESFYIKFSYNADVYEREEM
LRIQGHLKQALDCILTNPDVAVSDINIVPPEEQQVIQLFNETERPYVNKTIPQLFEEQARKTPEAAALKM
GDDCWTYRQLNIRANQIAHALIEKGVRSGDIVAVMMGRSMEMPAALLGIWKAGGAYMPLDPHFPAERLSF
LLKDSQAAQLLIEEDLISLIPPSYEGNTITIEHTESYQTEAPNMPPGDLAYLIYTSGTTGRPKGVLVDHH
GIANTLQWRREEYSMTEQDISLHLFSYVFDGCVTSLFTPLLSGACVLLTTDDEAKDVLALKRKIARYKVS
HMIIVPSLYRVLLEVMTADDAKSLRIVTFAGEAVTPDLLELSQMICPYAELANEYGPTENSVATTILRHL
NEKERITIGHPIRNTKVFVLHGNQMQPIGAAGELCISGAGLARGYYKQQELTQKAFSDHPFLEGERLYRT
GDAGRFLPDGTIEYIGRFDDQVKIRGYRIELREIETVLRQAPGVKEAAVLARDVSAEEKELVAYIVPEKG
NSLPDLYQHLAGTLPSYMIPASIINISRMPLTSSGKLDRFALPEPENNTSVTYMAPRTLIEADLAHIWED
VLNKQHIGIRDDFFQLGGQSLKATALVSRIHKKLNVELPLSEVFSYPTVESMAVKLMSLKEHAFTQIEPA
DQRDVYPLSFSQKRLYALHQLADDSTGYNMPAVLELRGNLNHQRLRSVLTELVNRHEALRTVFVLDRDEP
VQIIYPEIAFDLKELEMESEQMLESAIETFIKPFDLSSGPLFRACVITMGNNRGFLLLDMHHIIADGVSM
STLVQEFTDLYCGKELPALNLHYKDFAVWQQEKYPKELYKKQEAYWLGQLGGSLPTLELPLDKTRPRLPD
FSGGTIEVNIDKDMTDELHRLMAETGTTLYMILLAVYSILLSKLSGQEDIVVGSPAAGRPHADLERVIGM
FVNTLAMRSKPEGHKTFSSYLQDIRHLALTAYEHQDYPFEELADKVDTNREVNRNPLFDAMLVLQSSEDF
RFEVPGLSISSVTPKHDISKFDLTLHAEEHPSGIRCRFEYSTALFEEETITRWASYFIELVKGVTADTEM
RISDVQLLPAAERRLLLEKMGQYAAYPRNENIVSLFEKQAAKYPEHIAVVCGHSQLTYRDLNEKAERAAA
MLIKQGVRTGDIVGLMLDRSPDMIIGILSILKAGGAYLPIDPEYPQERISFMLNDSGAKLLLTERGQNKP
ADYTGHILYIDECENDSIPADANIQEIVTDQPAYVIYTSGTTGQPKGVIVEHSNVISLLKHQNLPFEFNH
EDIWTLFHSYCFDFSVWEMFGALLNGSTLVVVSKETARDPHAFRLLLKKERVTVLNQTPTAFYGLMHEDQ
NHPDRLNVRYVIFGGEALQPGMLQSWNKKYPHTDLINMYGITETTVHVTFKKLSAADIAKNKSNIGRPLS
TLQAYVMDAHMNLQPIGVPGELYIGGEGVARGYLNRDELTSDRFVSNPYLPGDRLYRTGDLARRLSNGEL
EYLGRIDEQVKVRGHRIELGEIQAALLQYPMIKEAAVITRADEQGQTAIYAYMVIKDQQAANISDIRTYL
KNALPDFMLPARMIQIDSIPVTVNGKLDQKALPEPEKHAYTADDISPRNEIETVMAEIWEELLNVDELGV
SANFFELGGDSIKALQVCARLKQRGFETTVREMFEHQTLGELSARVRKDVRAIDQGPVEGEITWTPIQQW
FFSQSLESHHFNQSVMIYRAERFDEAALRKVLKSLVTHHDALRIVCRHEDGRQVQINRGIDLSDEELYAL
ELFDVKDSLTEARNTIEEAASRMQEHIRLETGPLLHAGLFRTENGDHLFLTIHHLVVDAVSWRILFEDFS
AAYKQAVSGESIKLPQKTDSYLTYSQRIADYSKSRQVQREAAYWDECENRHIQPIPKDNDASSNTFKDTE

VIDFELSRHHTELLLTAAHKAYSTEMNDILLTALGLALQKWTGNNQFKISMEGHGRESYLEKIDISRTVG
WFTSIYPVWLDMRDSDHKDKEERLGHLIKQTKDMLHRIPHKGAGYGVLKYISKRWGSQKNSPEISFNYLG
QFDQDIQSNAFEVSDIKPGNEISPNWERPYALDISGAVSSGCLNMHIIYNRFQFEEKTIQTFSRHFKQTL
ENIIEHCTGKENHEWSASDFTDEDLTLDELSEIMGAVNKL
>Surfactant_-_surfactin~fenA~KAA0937139
MSEHTYSLTHAQRRVWFTELLEPDTSICNLTACVKFKGNIKLDTLEGALNHSISRNDAIRFQLLEGEELE
PRLHLTEYKYYPLRIIDFSNVEMIEIEQWIQDQASIPFKLFNSPLYQFYLLRIDSHEVWLFAKFHHIIMD
GISLNVMGNQIIDLYQKMKKKDPLPDQPEPSYLSYIEKESQYLQSPRFAKDRLFWTQTFEHPLEYHSLAD
QTSLQKQSTSASRDTIILSPDLEQTIRIFCEEHKINIISLFMASFYICISRITSKKDLAIGTYYGNRGSK
AEKEMLGMFVSSLPIRITVDPDTDFLSFVRTIGREQLSVMRHQRFPYNLLVNELRNEQKDLHNLIGISMQ
YQPLQWHNADDFDYETALYFSGYTANELSVQIQERIDNGTIQLNFDYQNTLFSLEDIKRIQSHLLTILEN
ALQHPHSFIRELDMTNTREKQKLLYEFNKTEAVSPKAFTLHGLFERQAAFTPERLAIRFSGGSLTYAELD
MYASRLAAHLAARGVTNESIVGVLSERSPDMLIAVLAVLKAGGAYLPLDPAYPKERLSYMLKDSGASLLL
TQPGCSAPNFSGETLEVDMTSLASEKAENHEFTPADGGSLAYVIYTSGSTGQPKGVAVEHRQAVSFLTGM
QHQFPLSEDDIVMVKTSFSFDASVWQLFWWSLSGASAYLLPPGWEKDPALIVKAIHQENVTTAHFIPAML
NSFLDQAEIERLSDRTSLKRVFAGGEPLAPRTAARFASVLPQVSLIHGYGPTEATVDAAFYVLDPERDRD
RLRIPIGKPVPGARLYVLDPHLAVQPSGVAGELYIAGAGVARGYLNRPALTEERFLEDPFYPGERMYKTG
DVARWLPDGNVEFLGRTDDQVKIRGYRIEPGEIEAALRSIEGVREAAVTVRTDSGEPELCAYVEGLQRNE
VRAQLERLLPGYMVPAYMIEMEQWPVTPSGKLDRNALPAPGGAADAETYTAPRNVTEMKLSQLWEDVLKN
GPVGIHDNFFDRGGHSLKATALVSRIAKEFDVQVPLKDVFAHPTVEGLATVIREGTDSPYEAIKPAEKQE
TYPVSSAQKRIYVLQQLEDGGTGYNMPAVLELEGKLNPERMERAFKELIKRHESLRTSFEQDAGGDPVQR
IHDEVPFTLQTTVLGERTEQEAAAAFIKPFDLSQAPLFRAQIVKISDERHLLLVDMHHIISDGVSVNILI
REFGELYNNRNLPALRIQYKDYAVWREGFKTGDAYKTQEAYWLKQLEGELPVLDLPADHARPPVRSFAGD
KVSFTLDQEVASGLHKLARENGSTLYMVLLAAYTAFLSRLSGQEDIIVGSPIAGRPHKDLEPILGMFVNT
LALRTRPEGGKPFVQYLQEVRETALEAFEHQDYPFEELVDKLELTRDMSRNPVFDAMFILQNVEKQDIDL
REIKVRPANFAHHISLFDITLIATEISGSICCEMEFSTEVFLKATIERWADHFIEFLHEALSTPETSLAQ
INILSDKEKQKIVFEFNKTQVEFAQKDVPFHRIFEAKAEEIPEHIAVIDNETEISYRLLNERANRLARTL
QNRKGPKPTVAVLAKRSIDAIVGVLAVMKAGGVYIPIDSHYPKARIEYILRDSGADILLSQQELKHLISN
LPESEMSHICLDDESTYEESNCNLNLSPAPEEPVYIIYTSGTTGAPKGVIVTYQNFTHAALAWRQIYELD
RKPVRLLQIASFSFDVFSGDLARTLTNGGTLIVCPDETRLEPAEIYKIMNSQRITVMESTPALIIPVMEY
VYRNQFKLPDLDMLILGSDMVKAQDFKTLTDRFGQSMRIINSYGVTEATIDSSFYETSMGGECTGDNVPI
GSPLPNVHMYVLSQTDQIQPIGVAGELCIGGAGVAKGYHHKPDLTQMKFTENPFVSGERLYRTGDRACWL
PNGTIRLLGRMDYQVKINGYRIETEEIESVLLQTGLVREAAVAVQHDKNGQAGLAAYIVPSDINTNALRA
ALTKELPAYMIPAHLIPLENMPLTLNGKLDRNALPAPNNVLSRPYTAPVNDIQKTMAYIWEDVLSMSRVG
IHDSFFELGGDSIKALQVAARLAAEGWSMTIRDLFRYSTIQELCGHITPLASQADQGPAEGEAELTPIQR
RFFGQVHAFHYHYNQSVMLFSEKGFNANALHLALRKITEHHDAIRMIFQRDQNGHVIQFNRGINHKDHEL
FGLYISDWTKASLERAHLDEKLAAEETVIQSKMNVEKGPLLQAGLFKTAEGDHLLIALHHLVIDGVSWRI
LLEDLAAAYQQALEKKEIQLPPKTDSYLSYADGLTQIAESKQLLSEKTYWQTILDAHTAFLPKDIENVPD
RLQMNSDAAAFVLSGDWTEKLLFETQQAYGTDANELLLMALGMALSEWTGHDQIVISTEGHGREGHVPNI
DISRTVGWFTSIYPILLDMVIPEPFEDQLAYRIKTTKDMLRRVPNKGTGYGLLTHIGELRHKEPEVSFNY
LGQFSEEKEADTFQLSYYQPRYEIAGEREREYELDINALITDGRLQVKAVYTQAFSKHSIECFMDRFHRH
LIETIEHCSQKKAREKTLSDFSNKELTLSALSSIEDLVKDL
>Surfactant_-_fengycin~srfAD~KAA0933188
MSQLFKSFDASEKTQLICFPFAGGYSASFRPLHAFLQGECEMLAAEPPGHGTNQTSAIEDLEELTDLYKQ
ELNLRPDRPFVLFGHSMGGMITFRLAQKLEREGIFPQAVIISAIQPPHIQRKKVSHLPDDQFLDHIIQLG
GMPAELVENKEVMSFFLPSFRSDYRALEQFELYDLAQIQSPVHVFNGLDDKKCIRDAEGWKKWAKDITFH
QFDGGHMFLLSQTEEVAERIFAILNQHPIIQP
>Surfactant_-_fengycin~srfAC~KAA0933189
MSQFSKDQVQDMYYLSPMQEGMLFHAILNPGQSFYLEQITMKVKGSLNIKCLEESMNVIMDRYDVFRTVF
IHEKVKRPVQVVLKKRQFQIEEIDLTHLTGSEQTAKINEYKEQDKIRGFDLTRDIPMRAAIFKKAEESFE
WVWSYHHIILDGWCFGIVVQDLFKVYNALREQKPYSLPPVKPYKDYIKWLEKQDKQASLRYWREYLEDFE
GQTTFAEQRKKQKDGYEPKELLFSLSEAETKAFTELAKSQHTTLSTALQAVWSVLISRYQQSGDLAFGTV
VSGRPAEIKGVEHMVGLFINVVPRRVKLSEGITFNGLLKQLQEQSLQSEPHQYVPLYDIQSQADQPKLID
HIIVFENYPLQDAKNEESSENGFDMVDVHVFEKSNYDLNLMASPGDEMLIKLAYNENVFDETFILRLKSQ
LLTAIQQLIQNPDQPVSTINLVDDREREFLLTGLNPPAQAHETKPLTDWFKEAVNVNPDAPALTYSGQTL
SYRELDEEANRLARRLQKHGAGKGSVVALYTKRSLELVIGILGVLKAGAAYLPVDPKLPEDRISYMLADS
AAACLLTHQEMKEQAAELPYTGTTLFIDDQTRFEEQASDPATAIDPNDPAYIMYTSGTTGKPKGNMTTHA

NIQGLVKHVDYMAFSDQDTFLSVSNYAFDAFTFDFYASMLNAARLIIADEHTLLDTERLTDLILQENVNV
MFATTALFNLLTDAGEDWMKGLRCILFGGERASVPHVRKALRVMGPGKLINCYGPTEGTVFATAHVVHDL
PDSISSLPIGKPISNASVYILNEQSQLQPFGAIGELCISGMGVSKGYVNRADLTKEKFIKNPFKPGETLY
RTGDLARWLPDGTIEYAGRIDDQVKIRGHRIELEEIEKQLQEYPGVKDAVVVADRPESGDASINAYLVNR
TQLSAEDVKAHLKKQLPAYMVPQTFTFLDELPLTTNGKVNKRLLPKPDQDQLAEEWIGPRNEMEETIAQI
WSEVLGRKQIGIHDDFFALGGHSLKAMTAASRIKKELGIDLPVKLLFEAPTIAGISAYVKNGGPDGLQDV
TIMNQDQERIIFAFPPVLGYGLMYQNLSSRLPSYKLCAFDFIEEEDRLDRYADLIQKLQPEGPLTLFGYS
AGCSLAFEAAKKLEEQGRIVQRIIMVDSYKKQGVSDLDGRTVESDVEALMNVNRDNEALNSEAVKQGLKQ
KTHAFYSYYVNLISTGQVKADIDLLTSGADFDMPEWLASWEEATTGAYRMKRGFGTHAEMLQGETLDRNA
GILLEFLNTQTVTVS
>Surfactant_-_fengycin~srfAB~KAA0933190
MSKKSIQKVYALTPMQEGMLYHAMLDPHSSSYFTQLELGIHGAFDLEIFEKSVNELIRSYDILRTVFVHQ
QLQKPRQVVLAERKTKVHYEDISHADENRQKEHIERYKQDVQRQGFNLAKDILFKVAVFRLAADQLYLVW
SNHHIMMDGWSMGVLMKSLFQNYEALRAGRTPANGQGKPYSDYIKWLGKQDNEEAESYWSERLAGFEQPS
VLPGRLPVKKDEYVNKEYSFTWDETLVARIQKTANLHQVTGPNLFQAVWGIVLSKYNFTDDVIFGTVVSG
RPSEINGIETMAGLFINTIPVRVKVERDAAFADIFTAVQQHAVEAERYDYVPLYEIQKRSALDGNLLNHL
VAFENYPLDQELENGSMEDRLGFSIKVESAFEQTSFDFNLIVYPGKTWTVKIKYNGAAFDSAFIERTAEH
LTRMMEAAVDQPAAFVREYGLVGDEEQRQIVEVFNSTKAELPEGMAVHQVFEEQAKRTPASTAVVYEGTE
LTYRELNAAANRLARKLVEQGLQKGETAAIMNDRSVETVVGMLAVLKAGAAYVPLDPALPGDRLRFMAED
SSVRMVLTGNSYTGQAHQLQVPVLTLDIGIEDGEADNLNLPSAPSDLAYIMYTSGSTGKPKGVMIEHKSI
LRLVKNAGYVPVTEEDRMAQTGAVSFDAGTFEVFGALLNGAALYPVKKETLLDAKQFAAFLREQSITTMW
LTSPLFNQLAAKDAGMFGTLRHLIIGGDALVPHIVSKVKQASPSLSLWNGYGPTENTTFSTSFLIDREYG
GSIPIGKPIGNSTAYIMDEQQCLQPIGAPGELCVGGIGVARGYVNLPELTEKQFLEDPFRPGERIYRTGD
LARWLPDGNIEFLGRIDNQVKVRGFRIELGEIETKLNMAEHVTEAAVIIRKNKADENEICAYFTADREVA
VSELRKTLSQSLPDYMVPAHLIQMDSLPLTPNGKINKKELPAPQSEAVQPEYAAPKTESEKKLAEIWEGI
LGVRAGVTDNFFMIGGHSLKAMMMTAKIQEHFHKEVPIKVLFEKPTIQELALYLEENESKEEQTFEPIRQ
ASYQQHYPVSPAQRRMYILNQLGQANTSYNVPAVLLLEGEVDKDRLENAIQQLINRHEILRTSFDMIDGE
VVQTVHKNISFQLEAAKGREEDAEEIIKAFVQPFELNRAPLVRSKLVQLEEKRHLLLIDMHHIITDGSST
GILIGDLAKIYQGADLELPQIHYKDYAVWHKEQTNYQKDEEYWLDVFKGELPILDLPADFERPAERSFAG
ERVMFGLDKQITAQIKSLMAETDTTIYMFLLAAFNVLLSKYASQDDIIVGSPTAGRTHPDLQGVPGMFVN
TVALRTAPAGDKTFAQFLEEVKTASLQAFEHQSYPLEELIEKLPLTRDTSRSPLFSVMFNMQNMEIPSLR
LGDLKISSYSMLHHVAKFDLSLEAVEREEDIGLSFDYATALFKDETIRRWSRHFVNIIKAAAANPNVRLS
DVDLLSSAETAALLEERHMTQITEATFAALFEKQAQQTPDHPAVKAGGNLLIYRELDEQANQLAHHLRAQ
GAGNEDIVAIVMDRSAEVMVSILGVMKAGAAFLPIDPETPEERIRYSLEDSGAKFAVVNERNMTAIGQYE
GTIVSLDDGKWRNESKERPSSISGSRNLAYVIYTSGTTGKPKGVQIEHRNLTNYVSWFSEEASLTENDKT
VLLSSYAFDLGYTSMFPVLLGGGELHIVQKETYTAPDEIAHYIKEHGITYIKLTPSLFHTIVNTSSFAKD
ANFESLRLIVLGGEKIIPTDVLAFRKMYGHTEFINHYGPTEATIGAIAGRVDLSDPDTFVKRPTIGRPIA
NAGALVLNEALKLVPPGASGQLYITGQGLARGYLNRPQLTAERFVENPYSPGSLMYKTGDVVRRLSDGTL
AFIGRADDQVKIRGYRIEPKEIETVMLSLSGIQEAVVLAVSEGGLQELCAYYTSDQAIEKAELRKQLSLT
LPSHMIPAFFVQVDAIPLTANGKTDRNALPKPNAAQSGGKALAVPETALEESLCRIWQKTLGIEAIGIDD
NFFDLGGHSLKGMMLIANIQAELEKSVPLKALFEQPTVRQLAAYMEASAVSGGHQVLKPADKQDMYPLSS
AQKRMYVLNQLDRQTISYNMPSVLLMEGELDISRLRDSLNQLVNRHESLRTSFMEANGEPVQRIIEKAEV
DLHVFEAKEDEADQKIKEFIRPFDLNDAPLIRAALLRIEAKKHLLLLDMHHIIADGVSRGIFVKELALLY
KGEQLPEPALHYKDFAVWQNEAEQKERMKEHEAYWMSVLSGELPELDLPLDYARPPVQSFKGDTIRFRTG
SETAKAVEKLLAETGTTLHMVLHAVFHVFLSKISGQRDIVIGSVTAGRTNADVQDMPGMFVNTLALRMEA
KEQQTFAELLEQAKQTNLSALEHQEYPFEDLVNQLDLPRDMSRNPLFNVMVTTENPDKEQLTLQNLSISP
YEAHQGTSKFDLTLGGFTDENGIGLQLEYATDLFAKETAEKWSEYVLRLLKAVADNPNQPLSSLSLVTET
EKQALLEAWKGKALPVPTDKTVHQLFEETVQRHKDRPAVTYNGQSWTYGELNAKANRLARILMDCGISPD
DRVGILTKPSLEMSAAVLGVLKAGAAFVPIDPDYPDQRIEYILQDSGAKLLLKQEGISVPDSYTGDVILL
DGSRTILSLPLDENDEENPETAVTAENLAYMIYTSGTTGQPKGVMVEHHALVNLCFWHHDAFSMTAEDRS
AKYAGFGFDASIWEMFPTWTIGAELHVIDEAIRLDIVRLNDYFETNGVTITFLPTQLAEQFMELENTSLR
VLLIGGDKLKRAVKKPYTLVNNYGPTENTVVATSAEIHPEEGSLSIGRAIANTRVYILGEGNQVQPEGVA
GELCVAGRGLARGYLNREDETAKRFVADPFVPGARMYRTGDLVKWTGGGIEYIGRIDQQVKVRGYRIELS
EIEVQLAQLSEVQDAAVTAVKDKGGNTAIAAYVTPESADIEALKSALKETLPDYMIPAFWVTLNELPVTA
NGKVDRKALPEPDIEAGSGEYKAPTTDMEDLLAGIWQDVLGMSEVGVTDNFFSLGGDSIKGIQMASRLNQ
HGWKLEMKDLFQHPTIEELTQYVERAEGKQADQGPVEGEVILTPIQRWFFEKNFTNKHHWNQSVMLHAKK
GFDPERVEKTLQALIEHHDALRMVYREENGDIVQAYKPIGESKVSFEIVDLYGSDEEMLRSQIKILANKL
QSSLDLRTGPLLKAEQYRTEAGDHLLIAVHHLVVDGVSWRILLEDFASGYMQAEKEESLVFPQKTNSFKD
WAEELAAFSQSAHLLQQAEYWSQIAAEQVSPLPKDCETEQRIVKDTSSVLCELTEEDTKHLLTDVHQPYG

TEINDILLSALGLTMKEWTKGAKIGINLEGHGREDIIPNVNISRTVGWFTAQYPVVLDISDADASAVIKT
VKENLRRIPDKGVGYGILRYFTETAETKGFTPEISFNYLGQFDSEVKTDFFEPSAFDMGRQVSGESEALY
ALSFSGMIRNGQFVLSCSYNEKEFERATVEEQMERFKENLLMLIRHCTEKEDKEFTPSDFSAEDLEMDEM
GDIFDMLEENLK
>Surfactant_-_fengycin~srfAA~KAA0933191
MEITFYPLTDAQKRIWYTEKFYPHTSISNLAGIGKLVSADAIDYVLVEQAIQEFIRRNDAMRLRLRLDEN
GEPVQYISEYRPVDIKHTDTTEDPNAIEFISQWSREETKKPLPLYDCDLFRFSLFTIKENEVWFYANVHH
VISDGISMNILGNAIMHIYLELASGSEAKEGISHSFIDHVLSEQDYAQSKRFEKDKAFWNKQFESVPELV
SLKRNASAGGSLDAERFSKDVPEALHQQILSFCEANKVSVLSVFQSLLAAYLYRVSGQNDVVTGTFMGNR
TNAKEKQMLGMFVSTVPLRTNIDGGQAFLEFVKDRMKDLMKTLRHQKYPYNLLINDLRETKSSLTKLFTV
SLEYQVMQWQKEEDLAFLTEPIFSGSGLNDVSIHVKDRWDTGKLTIDFDYRTDLFSREEINMICERMITM
LENALTHPEHTIDELTMISDAEKEKLLARAGGKSVSYRKDMTIPELFQEKAEQLSDHPAVVFEDRTLSYR
TLHEQSARIANVLKQKGVGPDSPVAVLIERSERMITAIMGILKAGGAYVPIDPGFPAERIQYILEDCGAD
FILTESKVAAPEADAELIDLDQAIAEGADDMTEADVNARNLAYIIYTSGTTGRPKGVMIEHRQVHHLVES
LQQTIYQSGSQTLRMALLAPFHFDASVKQIFASLLLGQTLYIVPKKTVTNGTALATYYRRNSIEATDGTP
AHLQMLAAAGDFEGLKLKHMLIGGEGLSSVVADKLLKLFKEAGTAPRLTNVYGPTETCVDASVHPVIPEN
AVQSAYVPIGKALGNNRLYILDQKGRLQPEGVAGELYIAGDGVGRGYLHLPELTEEKFLQDPFVPGDRMY
RTGDVVRWLPDGTIEYLGREDDQVKVRGYRIELGEIEAVIQQAPDVAKAVVLARPDEQGNLEVCAYVVQK
PGSEFAPAGLREHAARQLPDYMVPAYFTEVTEIPLTPSGKVDRRKLFALEVKAVSGTAYTAPRNETEKAI
AAIWQDVLNVEKAGVFDNFFETGGHSLKAMTLLTKIHKEAGVEIPLQYLFEHPTIAALAEEADHRESKAF
AVIEPAEKQEHYPLSLAQQRTYIVSQFEDAGVGYNMPAAAILEGPLDIQKLERAFQGLIRRHESLRTSFV
LENSTPRQNIHDSVDFNIEMIERGGRSDEAIMASFVRKFDLAKAPLFRIGLLGLEENRHMLLFDMHHLIS
DGVSIGIMLEELARIYKGEQLPELRLQYKDYAVWQSRQAAEGYKKDQAYWKEVFAGELPVLQLLSDYPRP
PVQSFEGDRVSIKLDAGVKDRLNRLAEQNGATLYMVMLSAYYTLLSKYTGQDDIIVGTPSAGRNHSDTEG
IIGMFVNTLAIRSEVKQDETFTQLISRVRKRVLNAFSHQDYPFEWLVEDLNIPRDVSRHPLFDTMFSLQN
ATAGIPAVGDLSLSVHETNFKIAKFDLTVQARETDEGIELDLDYSTKLFKQSTANRLLTHFARLLEDAAA
DPEKPISEYKLLSEEEAASQIQQFNPGRTPYPKDKTIVQLFEEQAANTPDHTALQYEGESLTYRELNERA
NRLARGILSLGAGEGRTAAVLCERSMDMIVSILAVLKSGSAYVPIDPEHPIQRMQHFFRDSGAKVLLTQR
KLKALAEEAEFKGVIVLADEEESYHADARNLALPLDSAAMANLTYTSGTTGTPKGNIVTHANILRTVKET
NYLSITEQDTILGLSNYVFDAFMFDMFGSLLNGAKLVLIPKETVLDMARLSRVIERENISILMITTALFH
LLVDLNPACLSTLRKIMFGGERASVEHVRKALQTVGKGKLLHMYGPSESTVFATYHPVDELEEHTLSVPI
GKPVSNTEVYILDRTGHVQPAGIAGELCVSGEGLVKGYYNRPELTEEKFVPHPFTSGERMYKTGDLARWL
PNGDIEFIGRIDHQVKIRGQRIELGEIEHQLQTHDRVQESVVLAVDQGAGDKLLCAYYVGEGDISSQEMR
EHAAKDLPAYMVPAVFIQMDELPLTGNGKIDRRALPIPDANVSRGVSYVAPRNGTEQKVADIWAQVLQAE
QVGAYDHFFDIGGHSLAGMKMLALVHQELGVELSLKDLFQSPTVEGLAQVIASAEKGTAASISPAEKQDT
YPVSSPQKRMYVLQQLEDAQTSYNMPAVLRLTGELDVERLNSVMQQLMQRHEALRTTFEIKDGETVQRIW
EEAECEIAYFEAPEEETERIVSEFIKPFKIDQLPLFRIGLIKHSDTEHVLLFDMHHIISDGASVGVLIEE
LSKLYDGETLEPLRIQYKDYAVWQQQFIQSELYKKQEEHWLKELDGELPVLTLPTDYSRPAVQTFEGDRI
AFSLEAGKADALRRLAKETDSTLYMVLLASYSAFLSKISGQDDIIVGSPVAGRSQADVSRVIGMFVNTLA
LRTYPKGEKTFADYLNEVKETALSAFDAQDYPLEDLIGNVQVQRDTSRNPLFDAVFSMQNANIKDLTMKG
IQLEPHPFERKTAKFDLTLTADETDGGLTFVLEYNTALFKQETIERWKQYWMELLDAVTGNPNQPLSSLS
LVTETEKQALLEAWKGKALPVPTDKTVHQLFEETAQRHKDRPAVTYNGQSWTYGELNAKANRLARILMDC
GISPDDRVGVLTKPSLEMSAAVLGVLKAGAAFVPIDPDYPDQRIEYILQDSGAKLLLKQEGISVPDSYSG
DVILLDGSRTILSLPLDENDEENPETAVTAENLAYMIYTSGTTGQPKGVMVEHHALVNLCFWHHDAFSMT
AEDRSAKYAGFGFDASIWEMFPTWTIGAELHVIEEAIRLDIVRLNDYFETNGVTITFLPTQLAEQFMELE
NTSLRVLLTGGDKLKRAVKKPYTLVNNYGPTENTVVATSAEIHPEEGSLSIGRAIANTRVYILGEGNQVQ
PEGVAGELCVAGRGLARGYLNREDETAKRFVADPFVPGERMYRTGDLVKWTGGGIEYIGRIDQQVKVRGY
RIELSEIEVQLAQLSEVQDAAVTAVKDKGGNTAIAAYVTPESADIEALKSALKETLPDYMIPAFWVTLNE
LPVTANGKVDRKALPEPDIEAGSGEYKAPTTDMEDLLAGIWQDVLGMSEVGVTDNFFSLGGDSIKGIQMA
SRLNQHGWKLEMKDLFQHPTIEELTQYVERAEGKQADQGPVEGEVILTPIQRWFFEKNFTNKHHWNQSVM
LHAKKGFDPERVEKTLQALIEHHDALRMVYREGQEDVIQYNRGLEAASAQLEVIQIEGQAADYEDRIERE
AERLQSSIDLQEGGLLKAGLFQAEDGDHLLLAIHHLVVDGVSWRILLEDFAAVYTQLEQGNEPVLPQKTH
SFAEYAERLQDFANSKAFLKEKEYWRQLEEQAVAAKLPKDRESGDQRMKHTKTIEFSLTAEETEQLTTKV
HEAYHTEMNDILLTAFGLAMKEWTGQDRVSVHLEGHGREEIIEDLTISRTVGWFTSMYPMVLDMKHADDL
GYQLKQMKEDIRHVPNKGVGYGILRYLTAPEHKEDVAFSIQPDVSFNYLGQFDEMSDAGLFTRSELPSGQ
SLSPETEKPNALDVVGYIENGKLTMSLAYHSLEFHEKTVQTFSDSFKAHLLRIIEHCLSQDGTELTPSDL
GDDDLTLDELDKLMEIF
